# Serum RNA biomarkers for predicting survival in non-human primates following thoracic radiation

**DOI:** 10.1038/s41598-022-16316-x

**Published:** 2022-07-19

**Authors:** Jared M. May, Uma Shankavaram, Michelle A. Bylicky, Sunita Chopra, Kevin Scott, Shannon Martello, Karla Thrall, Jim Axtelle, Naresh Menon, C. Norman Coleman, Molykutty J. Aryankalayil

**Affiliations:** 1 Radiation Oncology Branch, Center for Cancer Research, National Cancer Institute, Bethesda, MD USA; 2 Altasciences Preclinical Seattle LLC, Everett, WA USA; 3 ChromoLogic LLC, Monrovia, CA USA; 4 Radiation Research Program, National Cancer Institute, National Institutes of Health, Rockville, MD USA

**Keywords:** Predictive markers, Transcriptomics, Predictive markers

## Abstract

In a mass radiation exposure, the healthcare system may rely on differential expression of miRNA to determine exposure and effectively allocate resources. To this end, miRNome analysis was performed on non-human primate serum after whole thorax photon beam irradiation of 9.8 or 10.7 Gy with dose rate 600 cGy/min. Serum was collected up to 270 days after irradiation and sequenced to determine immediate and delayed effects on miRNA expression. Elastic net based GLM methods were used to develop models that predicted the dose vs. controls at 81% accuracy at Day 15. A three-group model at Day 9 achieved 71% accuracy in determining if an animal would die in less than 90 days, between 90 and 269 days, or survive the length of the study. At Day 21, we achieved 100% accuracy in determining whether an animal would later develop pleural effusion. These results demonstrate the potential ability of miRNAs to determine thorax partial-body irradiation dose and forecast survival or complications early following whole thorax irradiation in large animal models. Future experiments incorporating additional doses and independent animal cohorts are warranted to validate these results. Development of a serum miRNA assay will facilitate the administration of medical countermeasures to increase survival and limit normal tissue damage following a mass exposure.

## Introduction

In the event of a large-scale nuclear disaster or radiological event, either intentional or accidental, having an accurate method to conduct biological dosimetry will be paramount to triaging those affected, and effectively allocating limited resources to those in most need. Biological dosimetry, or biodose, refers to the biological changes an organism undergoes when exposed to radiation that correlates with functional changes which may resolve or may progress, the latter potentially subject to mitigation^1^. Partial or whole body exposures to large doses of radiation may cause extensive damage to rapidly proliferating tissue which culminates in death via acute radiation syndrome^2^. Those that survive may then progress to the delayed effects of acute radiation exposure (DEARE), with the lungs as the most sensitive organ^3^. The onset of DEARE symptoms varies, but generally appears after 30 days post-irradiation, with MacVittie et al. reporting a latent period of the DEARE-associated radiation induced lung injury manifestations at 60–70 days^4,5^. The window of opportunity for mitigation of DEARE requires a prompt diagnosis, which emphasizes the importance of a clinically useful biomarker. Despite being quantified in the same unit as physical dose, unit gray (Gy), biodose and physical dose are not equivalent in a heterogenous population^1^. Accurately determining physical dose is complicated in a nuclear scenario by variation in exposure, potential for multiple exposures, distance from epicenter and physical obstructions, among other variables. Additionally, the possibility of inhalation of airborne radioactive particles provides another route of exposure and further complications based on prolonged exposure^6^.

To best manage medical care in what likely would be a mass casualty incident with limited resources, it is necessary to triage patients based on medical need. In a heterogenous population, clinically observed symptoms of acute radiation syndrome such as vomiting, fever, and decreased white blood cell counts vary by case. While used in initial sorting of patients into risk categories, exposure and symptom triage tool (EAST), is not a sufficiently reliable indicator of biodose^7,8^. The current “gold standard” for measuring biological dosimetry measures formation of dicentric chromosomes or micronuclei in cultured lymphocytes, which is labor intensive and time-consuming, taking approximately three days to provide results^9^. Partial body exposure further complicates interpretation of this assay, with difficulties predicting low dose and small body fraction exposures^10^. At moderate to high radiation doses in humans (> 6 Gy), symptoms of acute radiation sickness can manifest in hours^11^, decreasing the effectiveness of medical countermeasures that would not be administered until results are received. At lower doses of radiation, symptoms may not immediately manifest but the window of efficacy for medical countermeasures will rapidly decrease as victims develop chronic injury to organs. Thus, the importance of developing a rapid and accurate method to measure biodosimetry is critical to mitigating loss of life and permanent damage in a nuclear disaster scenario. Serum, and the abundant miRNA information it contains^12,13^, has potential to become that rapid and accurate method, due to the ease of sampling and the correlations between serum miRNA and pathological conditions^14,15^.

MicroRNAs are transcripts between 19 and 22 nucleotides, have regulatory roles in transcription and translation and have been shown to be regulated by radiation damage. In a murine model of whole body radiation, Jacob et al. observed several time and dose dependent serum miRNA changes at 24 and 48 h after doses between 1 and 12 Gy^16^. Most notably, they observed a dose dependent decrease in miRNA-150-5p at both 24 and 48 h. miRNA-150-5p is abundant in lymphocytes and can predict lymphocyte depletion and hematopoietic injury. Tomasik et al. determined that there are 7 distinct miRNAs (miR-30a, miR-126, miR-133a, miR-133b, miR-150, miR-215, and miR-375) which have altered expression after irradiation and are conserved between non-human primates (NHPs, *Macaca mulatta*) and mice^17^. Additionally, they were accurately able to predict radiation exposure in NHP using a model with miR-133b, miR-215, and miR-375 and accurately predict mortality by modeling with miR-30a and miR-126, showing the promise of miRNAs as a diagnostic and prognostic marker for radiation damage to healthy tissue.

Similarly, Aryankalayil et al. demonstrated the repression of miR-17 family members mmu-miR-175p, mmu-miR-106b-5p, miR-20a/b-5p in whole blood samples from a murine model and the subsequent upregulation of their targets, *Cdkn1a*, *Stat3*, *E2f1*, *E2f3* and *Pten* at 24 and 48 h^18^. These results were then verified using a nonhuman primate model exposed to a whole-body irradiation dose of 4 Gy^18^.

Menon et al. corroborated the dose dependent decrease of miR-150 in an NHP model and also showed that miR-574-5p could distinguish lethal versus sublethal doses at 24 h post irradiation but returned to baseline levels by day 3^19^. This finding showed promise for early predictors of mortality, which will become critical in a mass casualty scenario where resources are limited. Additionally, they showed an upregulation of miR-126, miR-144, miR-21, miR-1-3p and miR-206 at 3- and 7-days post irradiation, providing helpful biomarkers of radiation exposure at later time points to aid a stressed healthcare system.

The judicious choice to study upper thoracic radiation injury in nonhuman primates is based on the sensitivity to radiation of the heart and lungs in radiotherapy for cancer. For patients with upper thoracic cancers receiving radiotherapy, the lungs are the dose-limiting organ^20^. Collateral damage to healthy tissue from upper thoracic radiotherapy can cause acute pneumonitis, esophagitis, and later pulmonary fibrosis^21^. Although less common than pneumonitis or esophagitis, upper thoracic radiotherapy is also associated with acute pericarditis and late stage cardiomyopathy, autonomic dysfunction, stenosis, atherosclerosis and arrythmias, among other symptoms^21^. Several studies have addressed biomarkers of radiation damage to the lungs, such as cytokines TGF-β1, interleukins, thrombomodulin, and KL-6^22^, but the effect on transcriptomics is still unclear. Because of the radiation dose-limiting aspect of the lungs, particularly for those who survive total body irradiation (TBI) due to effective therapy, it is proposed that radiation-induced lung damage will have a major role in predicting survival in a nuclear event, prompting the necessity of diagnosing the severity of lung injury. Indeed, pulmonary damage by radiation is generally the first consequence of DEARE that results in death^4,5^. Several miRNAs have been implicated in preventing or promoting radiation-induced lung fibrosis, a main concern of thoracic irradiation. The upregulation of miR-21 has been linked to the promotion of fibrosis^23^, the downregulation of miR-155-5p by irradiation has been linked to promoting fibrosis^24^, and the extracellular vesicle expression of miR-214-3p limited fibrosis^25^. Additionally, the majority of cardiac complications arise months to years after irradiation^26^ and chronic lung damage can manifest in 1–6 months after treatment^21^, so having an assay to detect biomarkers of thoracic radiation can determine prognosis and allow medical countermeasures to mitigate cardiac and pulmonary effects.

In this study, we assess the miRNA response to upper thorax irradiation of 9.8 and 10.7 Gy (LD_20/180_ and LD_75/180_, respectively) in healthy non-human primates over a comprehensive time frame, representative of the response in a nuclear incident using RNA sequencing. A parallel study using the same animals was recently published and examined clinical manifestations and several circulating miRNAs at early time points whose expression above or below the median forecasted greater likelihood of survival^27^. Several qualities make this study unique. The judicious choice to use an NHP model allows utilization of the best reproduction of human disease with a greater than 95% genomic similarity^28^. It is the first study assessing miRNA expression in NHP serum after irradiation over a comprehensive 270-day period. Lastly, and perhaps most uniquely, samples were collected at extended time points to assess long term changes in miRNA expression through a minimally invasive procedure to model a real nuclear scenario. Long term assessment of clinical manifestations and survival have allowed us to develop models to determine dose, length of survival and future development of pleural effusion. Additionally, this study examines the mRNA targets of miRNAs and the respective pathways whose dysregulation is most significantly correlated with survival. The overarching goal is to evaluate RNA biomarkers that can predict dosage, time of exposure, and ultimately mortality, to develop a high-throughput assay to assess lung and heart injury. This study aims to elucidate miRNA expression changes in serum samples and subsequent parallel studies will focus on whole blood RNA expression changes using the same animals.

## Materials and methods

### Animal selection

Male and female healthy adult Rhesus macaques (*M. mulatta,* Chinese substrain, n = 28) weighing between 3.5 and 5 kg (at initial physical exam) and aged between 3 and 5 years (at initiation of study) were used in this study. Animals were individually housed in cages that comply with the Animal Welfare Act and recommendations set forth in the Guide for the Care and Use of Laboratory Animals (National Research Council 2011). The animals were maintained in a temperature- and relative humidity- controlled environment between 18 and 29 °C and 30 and 70%, respectively. Animals were fed a diet of PMI LabDiet® Fiber-Plus® Monkey Diet 5049 biscuits twice daily and fasted prior to irradiation and examinations. Fruits, vegetables, and other dietary supplements were also provided throughout the course of the study. Fresh drinking water was provided ad libitum. Both diet and drinking water were routinely monitored for contaminants that may affect the outcome of the study, but no such contaminants were found. This study was performed in an Association for Assessment and Accreditation of Laboratory Animal Care (AAALAC) approved laboratory with Institutional Animal Care and Use Committee approval. SNBL USA has an Animal Welfare Assurance issued by the Office of Laboratory Animal Welfare (OLAW) and is registered with the United State Department of Agriculture. The study is in accordance with the Animal Welfare Act and recommendations set forth in the Guide for the Care and Use of Laboratory Animals (National Research Council 2011). This study is reported in accordance with ARRIVE guidelines (https://arriveguidelines.org).

### Animal irradiation

Animals were assigned to treatment groups via a stratified randomization scheme using body weights and were quarantined and acclimated to the study room for 14 days prior to photon beam irradiation. Animals were irradiated under the careful supervision of a Medical Physicist. Quality assurance was performed in the morning prior to irradiation. Irradiation was performed using a Varian CLINIC 21EX linear accelerator (maximum of 6 MV). Three male and three female NHPs were randomly selected for Group 1 and four males and four females were randomly selected for Group 2. Group 1 received a total dose of 9.8 Gy, delivered to the thorax at a rate of 600 ± 10 cGy/min. Group 2 received a total dose of 10.7 Gy, delivered to the thorax at a rate of 600 ± 10 cGy/min. The doses of 9.8 Gy and 10.7 Gy were designed to mimic LD_20/180_ and LD_75/180_ respectively.

Radiation treatment was planned by acquired thorax CT scan images (200 kV potential, 110 mAs, 1.5 rotation time, 1.0 pitch level, B70s kernel, and 1.5 mm slice thickness) and Varian Eclipse TPS v13.6 treatment planning software. On the day of irradiation, food was withheld at least 12 h prior to irradiation. Between 15 and 90 min prior to irradiation, animals were administered an antiemetic (Ondansetron HCl, 1.0 mg/kg) by IM injection. Animals were then anesthetized with ketamine/xylazine prior to transportation to the irradiator. Sedated animals were placed on the irradiator couch supine and head to gantry with arms overhead. The gantry was at 180 degrees (facing the floor) and rotated 180 degrees at half dose. Imaging confirmed the animal’s position prior to irradiation. The dose was administered with approximately 50% contribution from both the anterior–posterior and posterior-anterior beams.

### Veterinary treatments

Cage-side clinical observations were performed twice daily with at least 6 h in between observations. Veterinary physical exams were performed once during acclimation and once every 30 ± 3 days while animals were sedated for CT scans, assessing for body condition, hydration, capillary refill time, rectal temperature, heart rate, respiratory rate, and organ system function. Non-sedated pulse oximetry, rectal temperature, and respiratory rates were measured twice weekly, once weekly, and every 3 days, respectively.

All animals received oral tramadol (1–4 mg/kg: approximately ¼ of a 50 mg tablet) twice daily or buprenorphine (0.01 mg/kg) by intramuscular (IM) injection twice daily beginning on post-irradiation Day 4 and continuing through Day 25. Tramadol was the preferred drug and was mixed with food or treats when necessary. Animals refusing oral administration for two consecutive treatments were switched to buprenorphine for the remainder of the treatment period. Animals in distress after tramadol or buprenorphine (0.01 mg/kg, twice daily) received 0.02 mg/kg of buprenorphine twice daily.

Animals that developed tachypnea indicative of pneumonitis (non-sedated respiratory rate ≥ 80 breaths per minute) were treated with a corticosteroid taper. At first episode, dexamethasone was administered IM using the following regimen: 1.0 mg/kg twice daily on the first day; 0.5 mg/kg twice daily for three days; 0.5 mg/kg once daily for three days; and 0.5 mg/kg every other day for three doses. At second episode (within 7 days of stopping first course of treatment), animals received dexamethasone IM using the following regimen: 1.0 mg/kg once on the first day; 0.5 mg/kg once daily for four days; and 0.5 mg/kg every other day for 10 doses. Any animal that completed two courses of corticosteroid treatment and had an additional tachypnea episode and was non-responsive to treatments met the criteria for euthanasia. Once an animal was marked for euthanasia they were sedated and then injected with an overdose of euthanasia solution.

Euthanasia was justified on veterinary exam by the presence of one of a “single criterion” or two or more “combination criteria.” Single criteria included unrelieved pain or distress following administration of two consecutive increased doses of buprenorphine (0.02 mg/kg IM twice daily), inactivity for greater than 15 min, respiratory distress (labored breathing, open mouth breathing, respiratory rate ≥ 80 breaths per minute and non-responsive to treatment), uncontrolled hemorrhage from any orifice, or signs of severe dehydration (evidenced by skin tent time > 3 s, sunken eyes, rapid and weak pulse, cold extremities, and/or comatose). Combination criteria included loss of body weight ≥ 25% of baseline for two consecutive days, severe injury, hyperthermia (rectal temperature ≥ 41 °C), hypothermia (rectal temperature ≤ 35 °C), or complete anorexia for 48 h.

Severity score for lung and heart was determined at necropsy based on the following criteria. For heart, evidence of heart lesions, fibroplasia in the interstitium, degeneration of myocardium, infiltration of mononuclear cells, hemorrhaging, fibroplasia of epicardium, and fibrin deposition in interstitium were used in scoring severity. For lung, evidence of fibroplasia in interstitium, infiltration of macrophages into alveolar bronchioles, fibroplasia in the pleura, edema in alveolar spaces, infiltration of mononuclear cells in the interstitium, hyperplasia of bronchioles, fibrin deposition in the alveolar space, hemorrhage in alveolar space, mononucleated macrophage presence, and infiltration of mixed inflammatory cells were used in scoring severity.

### Blood/serum collection

Approximately 3.5 mL of blood was drawn from a peripheral vein of conscious animals. The site was disinfected with an alcohol swab and lanced firmly by pressing the lancet against the skin. The first drop of blood was wiped with an alcohol swab and drawn into a 4 mL K_2_EDTA tube on wet ice. Within 30 min of collection, blood samples were centrifuged at room temperature at 3000*g* for 17 min and serum was transferred to Simport polypropylene 1.5 mL cryovials in 0.5 mL aliquots and stored at − 86 to − 60 °C. Samples were shipped to the National Cancer Institute (Bethesda, MD) on dry ice.

**Figure 1:**
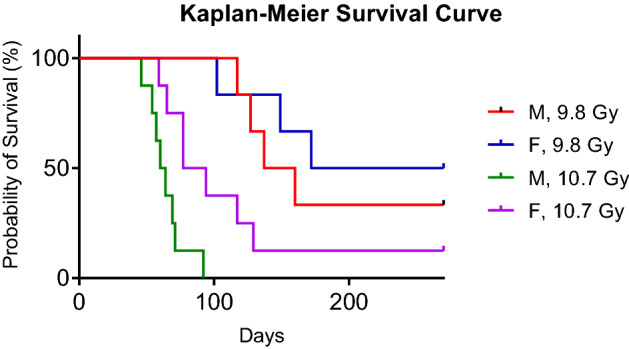
Kaplan–Meier survival curve for the NHPs involved in this 270-day study. High doses had lower survival than low doses and males had a worse prognosis than females.

Blood samples were collected from each of the animals (n = 28) two days prior to irradiation to serve as controls, every three days after irradiation for the first 30 days, at days 40, 50, and 60, and approximately every 30 days until all surviving animals were euthanized at day 270 after irradiation. Animals exhibiting signs of severe acute radiation syndrome in between formal blood collection time points were euthanized and a final sample was drawn for that animal. Animals exhibiting severe acute radiation syndrome at formal collection time points were scheduled for terminal necropsy and a final blood sample was drawn. Survival data can be found in Fig. 1.

### RNA isolation

Total small RNA, including miRNAs, were isolated from the serum samples using miRNeasy Serum/Plasma Kit (QIAGEN, Cat No./ID: 217184) according to the manufacturer’s protocols. The total small RNA was eluted in 14 µL RNase-free water by centrifuging at full speed for 1 min and stored at -80 ºC until RNA sample was shipped on dry ice for sequencing. Supplemental Table 1 lists the number of samples analyzed at each timepoint for each group, including terminal samples taken between formal timepoints (e.g. day 71).

**Table 1:** Study day of death, euthanasia criteria (if applicable), and average lung and heart severity of findings scores for each animal included in the study.

Dose	Gender	Animal number	Study day of death	Euthanasia criteria (if applicable)	Average severity of lung findings	Average severity of heart findings
9.8 Gy	Male	1001	127	Respiratory distress; non-responsive to treatment	1.08	0.43
		1002	160	Respiratory distress; non-responsive to treatment	0.67	0.43
		1003	270*	*N/A	**0.25**	**0**
		1004	270*	*N/A	**0.25**	**0**
		1005	137	Found dead	1.50	0.14
		1006	117	Respiratory distress; non-responsive to treatment	1.08	0.14
	Female	1501	149	Respiratory distress; non-responsive to treatment	0.75	0.14
		1502	270*	*N/A	**1.00**	**0**
		1503	270*	*N/A	**1.00**	**0**
		1504	102	Respiratory distress; non-responsive to treatment	0.75	1.00
		1505	270*	*N/A	**0.75**	**0**
		1506	172	Respiratory distress; non-responsive to treatment	0.91	0
10.7 Gy	Male	2001	92	Respiratory distress; non-responsive to treatment	0.75	1.57
		2002	69	Respiratory distress; non-responsive to treatment; recumbent; died prior to euthanasia	0.67	0.43
		2003	60	Recumbent ≥ 15 min	0.33	0.57
		2004	71	Recumbent ≥ 15 min	0.75	0.43
		2005	54	Found dead	0.50	0.14
		2006	64	Recumbent ≥ 15 min	0.75	0.43
		2007	46	Respiratory distress; non-responsive to treatment; recumbent	0.50	0.14
		2008	57	Found dead	0.75	0.57
	Female	2501	77	Respiratory distress; non-responsive to treatment	0.83	0.86
		2502	270*	*N/A	**0.85**	**0.14**
		2503	59	Found dead	1.08	0.57
		2504	94	Respiratory distress; non-responsive to treatment	0.75	0.71
		2505	65	Recumbent ≥ 15 min	0.50	0.57
		2506	129	Recumbent ≥ 15 min	1.08	0.29
		2507	117	Respiratory distress; non-responsive to treatment	0.75	0.43
		2508	77	Recumbent ≥ 15 min	1.00	0.43

Bold typeface indicates data for the six animals that survived the length of the study.

*Animal survived the length of the study (270 days) and was euthanized when study terminated. Criteria for lung and heart severity score are noted in Methods section.

### miRNA analysis via RNA-sequencing

Total small RNA samples were assessed for quality and quantity using Bioanalyzer 2100 Eukaryote Total RNA Nano kit (Agilent Technologies, CA, USA). Small RNA library was prepared for transcripts 18–40 bp and miRNA counts were determined by sequencing on the Illumina HiSeq platform (Novogene Co., South Plainfield, NJ).

### Data analysis

Sequences were aligned to the *Macaca mulatta* genome at the Center for Cancer Research Genomics Core (Bethesda, MD). Data was processed and subsequent analyses performed in R^29^. Raw miRNA count data was filtered to remove counts < 20 and normalized by mean variance method (limma(voom))^30^, adjusted for library size. The lmFit function was used to fit row-wise linear models. The lowess function was then used to fit a trend to the square-root-standard-deviations as a function of an average log-count measure. A trend line was used to predict the variance of each logCPM value as a function of its fitted value on the count scale, and the inverse variances become the estimated precision weights. Data quality was assessed before and after normalization. Because the samples were sequenced in three separate batches, potential batch effects were investigated with no significant differences between batches. The combined effect of dose and time on gender was tested using 2-way ANOVA. One-way ANOVA followed by Dunnett’s test was used to compare differences in male vs. female in high and low dose groups, and pairwise T-tests were used to compare differences at each time point in various analyses described in the results. For prediction of radiation sensitivity on time course or on survival, data was processed first to select optimal features for a given comparison using Boruta algorithm in R^31^ followed by elastic net analysis. Ingenuity Pathway Analysis (QIAGEN) was used for pathway analysis, based on miRNA expression profiles of samples at the given time points.

### Predicting target genes

Ingenuity Pathway Analysis (QIAGEN) was used for pathway analysis based on miRNA expression profiles of samples at the given time points. FunRich (functional enrichment) is an analysis tool used for functional enrichment and protein–protein interaction network analysis for genes or proteins. The microRNA enrichment function in FunRich could be used to perform miRNA enrichment analysis, to predict targets of microRNAs, or to find microRNAs through given target genes. Functional analysis of differentially expressed microRNA target genes for comparisons greater or less than 60 days was conducted for: (a) overall samples (taking all samples into consideration), (b) Male and (c) Female to predict target genes with FunRich^32^.

### Quantitative RT-PCR

Five female animals (time-points: D-2, D3 and D6) irradiated with 10.7 Gy were selected for qRT-PCR validation. RT-PCR validation was performed using miRCURY LNA RT Kit (QIAGEN, Cat No./ID: 339340) and miRCURY SYBR Green PCR Kit (QIAGEN, Cat No./ID: 339345) per the manufacturer protocols. miRCURY LNA PCR primer assays were purchased from Qiagen (Cat No. 339306) for miR-454-3p, let-7c-5p, miR-34a-5p, miR-26b-5p, let7g-5p and miR-17-5p. The UniSp2,4,5 spike-in controls were added during RNA isolation. Threshold Ct values for UniSp2, 4, and 5 were used to compare RNA isolation efficiencies. UniSp4 and sRNA 5S gene were used for normalization as follows.

$$\begin{aligned} & {\text{dCT}}_{{{\text{sample1}}}} = {\text{CT}}_{{\text{5S-sample1}}} - {\text{CT}}_{{\text{UniSp4-sample1}}} \\ & {\text{ddCT}}_{{{\text{sample1}}}} = {\text{CT}}_{{{\text{miRNA}}\_{\text{sample1}}}} -{\text{dCT}}_{{{\text{sample1}}}} \\ & {\text{dddCT}}_{{{\text{miRNA}}\_{\text{sample1}}}} = {\text{ddCT}}_{{{\text{miRNA}}\_{\text{sample1}}}} -{\text{ddCT}}_{{{\text{miRNA}}\_{\text{control}}}} \\ & {\text{Foldchange}} = {2}^{{ - {\text{dddCT}}}} \\ \end{aligned}$$

For calculating differences across control samples, all control sample fold changes were calculated using average control CT values using the method detailed above. Paired two-tailed t-tests were calculated between control values and D3 and D6 values.

## Results

### Dose impacted long-term survival post-irradiation, but gender was insignificant

Figure 1 displays the Kaplan–Meier Curve for the current study, with animals grouped by gender and dose (9.8 Gy vs. 10.7 Gy). High radiation dose was associated with lower survival. Additionally, male NHPs had a worse prognosis than females, with no males from the high dose group surviving the length of the study (270 days) and only 2 males (33%) surviving in the low dose group. Female NHPs displayed a 50% survival rate at 270 days in the low dose group, but only a 12.5% survival rate at 270 days in the high dose group. However, single-factor ANOVA revealed the differences between survival lengths by gender were statistically insignificant for low dose (α = 0.05, p = 0.5591) and high dose (α = 0.05, p = 0.0795). The discrepancies between low dose and high dose survival were statistically significant for males (α = 0.05, p = 0.0006) and females (α = 0.05, p = 0.0299).

### Clinical manifestations include lung and heart damage

Table 1 lists the dose, gender, length of survival, euthanasia criteria (if applicable), and necropsy findings for each individual animal in the study. All animals in the low dose group that died demonstrated respiratory distress and were non-responsive to treatment, meeting criteria for euthanasia. Respiratory distress and non-responsiveness to treatment were also prevalent in the high dose group in roughly half of the deceased animals, with the other half euthanized due to recumbency for ≥ 15 min. By comparing the necropsy lung severity scores between animals that survived and those that did not using ANOVA, there were no significant differences. However, there were significant differences (p = 0.004) between the heart severity scores of animals that survived versus those that did not. When comparing lung severity scores by gender and survival (2 genders, 2 survival statuses, 4 groups total), there were significant differences (p = 0.026) between the groups via ANOVA, but there was no correlation between higher scores and death. Instead, females had higher lung scores than males. When comparing heart severity scores by gender and survival, there were again significant differences (p = 0.048) between groups. In hearts, however, higher severity scores correlated with death before the end of the study.

Time course clinical data for each animal, available upon request, indicated early symptoms of lung and heart dysfunction prior to death. In general, all animals maintained an oxygen saturation of 95% until day 100, when surviving animals began to gradually demonstrate decreased oxygen saturation. Similarly, the average respiratory rate increased in all animals around days 40–45 post-irradiation, with a more pronounced effect in higher dose animals. Additionally, surviving low dose animals had an average rate of 60 breaths per minute compared to non-surviving low dose animals at 88 breaths per minute at Day 123 post-irradiation. There were no correlations to dose or survival based on heart rate.

All animals were managed with at least one course of dexamethasone treatment, with higher dose animals overall requiring treatment sooner. One low dose male (animal 1004) survived the length of the study after six separate courses of dexamethasone. Nine out of the 15 high dose animals that died only received one course of dexamethasone before succumbing to their injuries.

Radiation-induced pneumonitis was identified in 24 out of 28 animals with involvement at some point in every lobe of the lung by CT scan. The four animals without evidence of pneumonitis were all high dose males. Pleural effusion was present in 17 out of 28 animals, including animals from both groups. Two low dose (1002, 1502) and one high dose animal (2503) also displayed pericardial effusion. Low dose animals also had a greater normal lung volume than high dose animals (92.2 vs. 88.1%).

### Significant miRNA expression varied over the 270-day interval

**Figure 2:**
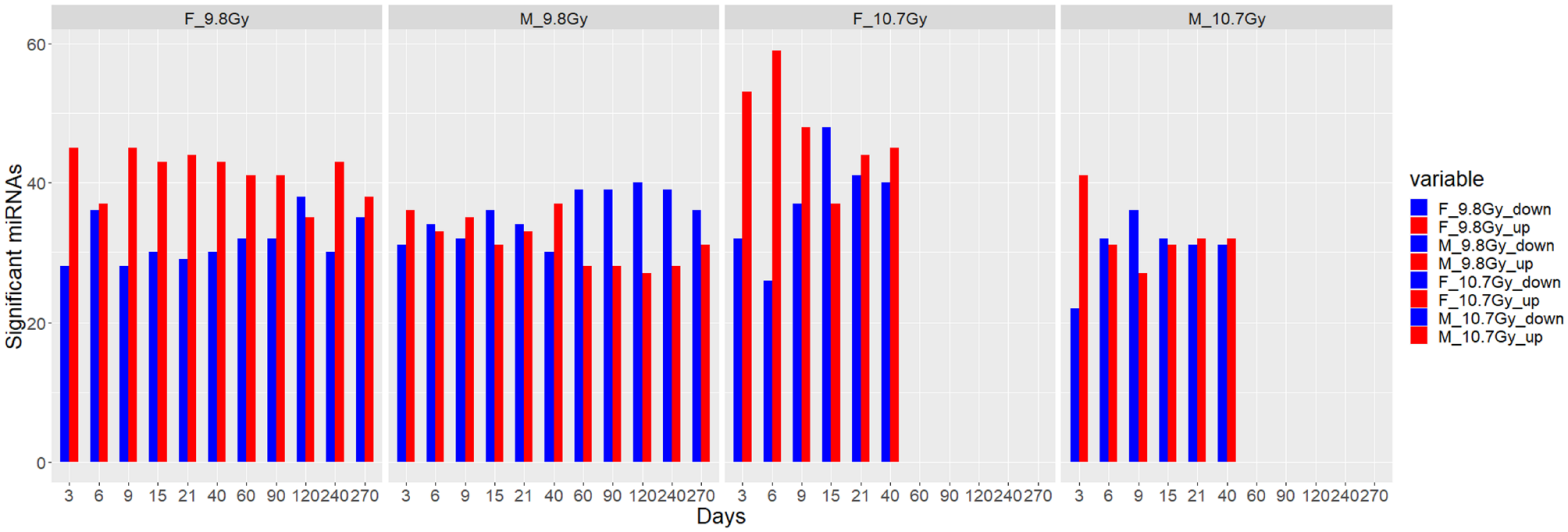
Number of significant miRNAs for each group and timepoint. Samples were separated based on sex and dose. Upregulation is indicated in red. Downregulation is indicated in blue. The significant expression of miRNAs varied over the 270-day course of the study.

One-way ANOVA revealed the number of significant miRNAs (p ≤ 0.05) relative to the control samples for each dose at each time point. Figure 2 displays the number of significant miRNAs for each group over the 15 timepoints sampled. The number of significant miRNAs varied over the course of the study, with a relatively large number expressed in each group at Day 21. There were no significant differences between the groups, confirmed by single-factor ANOVA (α = 0.05, p = 0.1386). In Supplemental Table 2, the number of miRNAs common to both doses for each gender is presented. For example, there were 21 miRNAs at Day 3 in females that were expressed in both doses. We performed qRT-PCR for a select group of differentially expressed miRNAs (miR-454-3p, let-7c-5p, miR-34a-5p, 26b-5p, let-7 g-5p and miR-17-5p) in five female animals irradiated with 10.7 Gy to validate the RNA seq data (Supplemental Fig. 1). An upregulation in the levels of all miRNAs tested was observed at D6 compared to control values mimicking the RNA seq trends. The upregulation was noticeable even when miRNAs lacked significant p-values. Ith could be attributed to smaller sample size investigated.

**Table 2:** Accuracy, no-information rate, and p-values for the five prediction models developed in this study.

Category	Model	Day	Accuracy (%)	No information rate (%)	p-value	miRNAs (non-zero coefficients)
Dose	Predict if animal received Control (0 Gy), Low Dose (9.8 Gy) or High Dose (10.7 Gy)	3	68	NA	NA	MIR214, MIR664, MIR30E, MIR34A, MIR196B, MIR219.1, MIR375, MIR454, MIR196A.1, MIR16.1, MIR372
		6	72	54	0.18	MIR34A, MIR320A, MIR26B, MIR34C, MIR181A.2, MIR454, MIR338, MIR98
		9	70	60	0.38	MIR181A.1, MIR34A, MIR200A, MIR191, MIR335, MIR376A.1, MIR30B, MIR454, MIR16.1
		15	81	45	0.01	MIR7180, MIR205, MIR34A, MIR200A, MIR106B, MIR7186, MIR30B, MIR26A.2, MIR181A.2, MIR301A, MIR338
		21	72	54	0.18	MIR197, MIR34A, MIR26B, MIR126, MIR27B, MIR301A, MIR10A, MIR17
Survival (3 groups)	Both genders together, predict how long animals survived: Died < 90 days (n = 11), died from 90–269 (n = 11), or survived 270 days (n = 6)	3	50	50	0.63	MIR30E, MIR219.1, MIR584, MIR491, MIR195, MIR372, MIR222, MIRLET7F.2, MIR19B.2
		6	12	50	0.99	MIR3122, MIR802, MIRLET7I, MIR26B, MIR450B
		9	71	47	0.04	MIR340, MIR452
		15	62.5	50	0.36	MIR28, MIR539, MIRLET7A.2, MIR873, MIR454, MIR501, MIR505
		21	50	62.5	0.86	MIR339, MIR135A.2, MIRLET7F.2
Survival (4 groups)	Both genders together, predict how long animals survivied: Died = 60 days (n = 9), Died from 61–120 (n = 8), Died > 120 (n = 5), survived (n = 6)	3	62.5	37	0.13	MIR218.2, MIR627, MIR342, MIR599, MIR324
		6	50	37	0.34	MIR1262, MIR29B.1, MIR1296, MIR374A
		9	62.5	50	0.36	MIR1260B, MIR181B.2, MIR502
		15	25	NA	1	MIR451, MIR505
		21	62.5	75	0.88	MIR215, MIR489, MIRLET7A.3
Pleural Effusion (+/−PE)	Both genders together, predict if animal develops pleural effusion (PE)	3	50	100	1	MIR218.2, MIR627, MIR342, MIR599, MIR324
		6	50	100	1	MIR1262, MIR29B.1, MIR1296, MIR374A
		9	75	62	0.36	MIR5697, MIR106B, MIR181A.2
		15	75	75	0.67	MIR543, MIR605, MIRLET7A.3, MIR502
		21	100	50	0.003	MIR665, MIR7.1, MIR324, MIR181D
Pleural Effusion (+/−PE by gender)	By gender, predict development of pleural effusion	3	50	37	0.34	MIR206, MIR874, MIR378A, MIR1240, MIR181C
		6	87	37	0.005	MIR196B, MIR654, MIR376A.1, MIR154, MIR412, MIR1296, MIR500B, MIR98
		9	75	50	0.14	MIR320B, MIR1262, MIR6827, MIR3146, MIR143, MIR323A, MIR485, MIR369, MIR10A
		15	75	37	0.03	MIR29B.2, MIR885, MIR671, MIR219.1, MIR493, MIR329.1, MIR543, MIR654, MIR154, MIR605, MIR193B
		21	62	50	0.36	MIR9.1, MIR9.2, MIR218.2, MIR495, MIR410, MIR615, MIR24.1, MIR7.3, MIR181D, MIR452

Significant results included the Control vs. LD vs. HD model at Day 15, the three-group survival model at Day 9, the pleural effusion model at Day 21, and the pleural effusion model by gender at Days 6 and 15.

### Top-level 2-way ANOVA reveals unique gender markers for both doses

**Figure 3:**
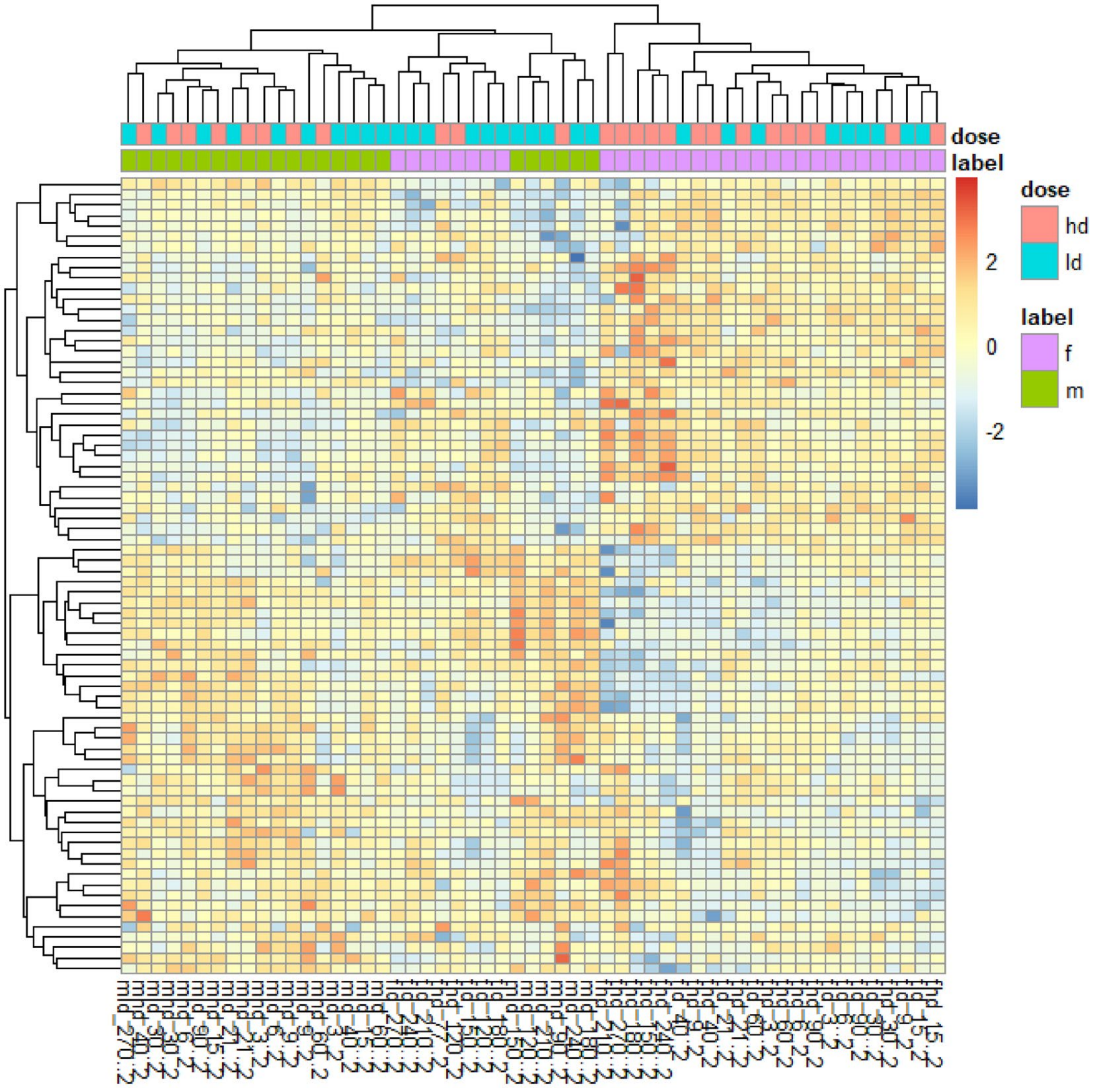
Two-way top-level ANOVA revealed the 76 miRNAs that are expressed by both doses and both genders at any time point (p ≤ 0.05). Although these miRNAs are common between males and females at any timepoint, the expression pattern by time segregates well by gender.

Two-way ANOVA was performed to determine top-level low dose and high dose gender-specific markers, controlling for the time effect. This was intended to yield major differences between genders for each dose. At high doses, 64 miRNAs exhibited significant (p ≤ 0.05) gender-specific differences, such as fold changes in opposite directions. Similarly, at low doses, 53 miRNAs exhibited significant gender-specific differences. Additionally, 76 miRNAs were differentially expressed by both genders at both doses. These 76 miRNAs are shown in Supplemental Fig. 2. The ten most statistically significant of these miRNAs included miR-122a, miR-139, miR-224, miR-769, miR-95, miR-885, miR-29c, miR-1225, let-7c, and miR-335. A heat map of the 76 miRNAs common between both genders at both doses is shown in Fig. 3. The average expression of each for the 76 miRNAs of each member sampled from the group (LD and HD, male and female) for each timepoint is shown.

### One-way ANOVA reveals further dose and gender differences in miRNA expression

**Figure 4:**
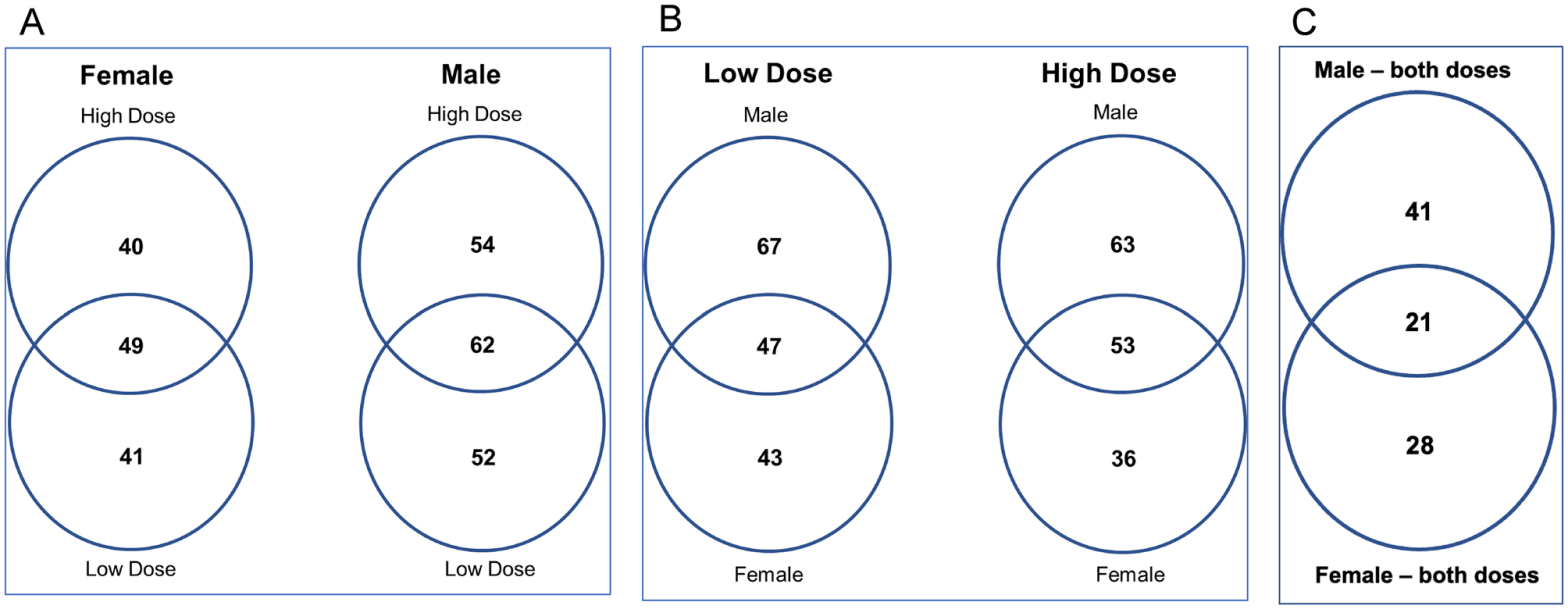
(**A**) miRNA radiation markers by dose. One-way ANOVA for each gender identified the number of significant (p ≤ 0.05) miRNAs for each condition, at any time point. (**B**) miRNA radiation markers by gender. One-way ANOVA for each gender identified the number of significant (p ≤ 0.05) miRNAs for each condition, at any time point. (**C**) The intersection of Figs. 4 and 5 by one-way ANOVA followed by Dunnett’s test (p ≤ 0.05). There are 21 miRNAs that are significantly regulated in both genders at either dose by time.

Figure 4A,B represent the number of significant miRNAs (omnibus, p ≤ 0.05) from one-way ANOVA by dose and by gender, respectively. In Fig. 4A, female NHPs had 40 miRNAs specific to high dose, 41 miRNAs specific to low dose, and 49 miRNAs in common between both doses. In males, 54 miRNAs were specific to high dose, 52 miRNAs were specific to low dose, and 62 miRNAs in common between both doses. The low dose NHPs had 67 miRNAs specific to males, 43 miRNAs specific to females, and 47 miRNAs in common between both genders (Fig. 4B). The high dose NHPs had 63 miRNAs specific to males, 36 miRNAs specific to females, and 53 miRNAs in common between both genders. Figure 4C displays the intersection between Fig. 4A,B. There are 41 miRNAs unique to males and 28 miRNAs unique to females at both doses. Additionally, there are 21 miRNAs that have shared expression between both genders and either dose by time. The detailed list of the miRNAs at the intersections of Fig. 4A–C are available in Supplemental Table 3.

### miRNA expression can differentiate between high and low doses regardless of time or gender

Student’s t-test was performed on combined data (all time points and both genders) for high dose versus low dose to determine markers that were significantly different between doses. In total, 78 markers were statistically significantly different (p ≤ 0.05) between high dose and low dose samples. Of the 78 miRNAs, 16 displayed a dose-dependent downregulation while 10 displayed a dose-dependent upregulation. An additional eight were downregulated at both doses and an additional 12 were upregulated at both doses. The identities, mean values, and fold changes of the dose-dependent miRNAs for the combined data are shown in Supplemental Table 4, with the 16 dose-dependent downregulated miRNAs highlighted in blue and the 10 dose-dependent upregulated miRNAs highlighted in red. Of the 26 dose-dependent miRNAs five, miR-26b, miR-34a, miR-30a, miR-34c, and miR-454, were also identified in Fig. 4C as significantly regulated in both genders at either dose by time.

### miRNA signatures can differentiate between control, high dose, and low dose samples in first 21 days after exposure

**Figure 5:**
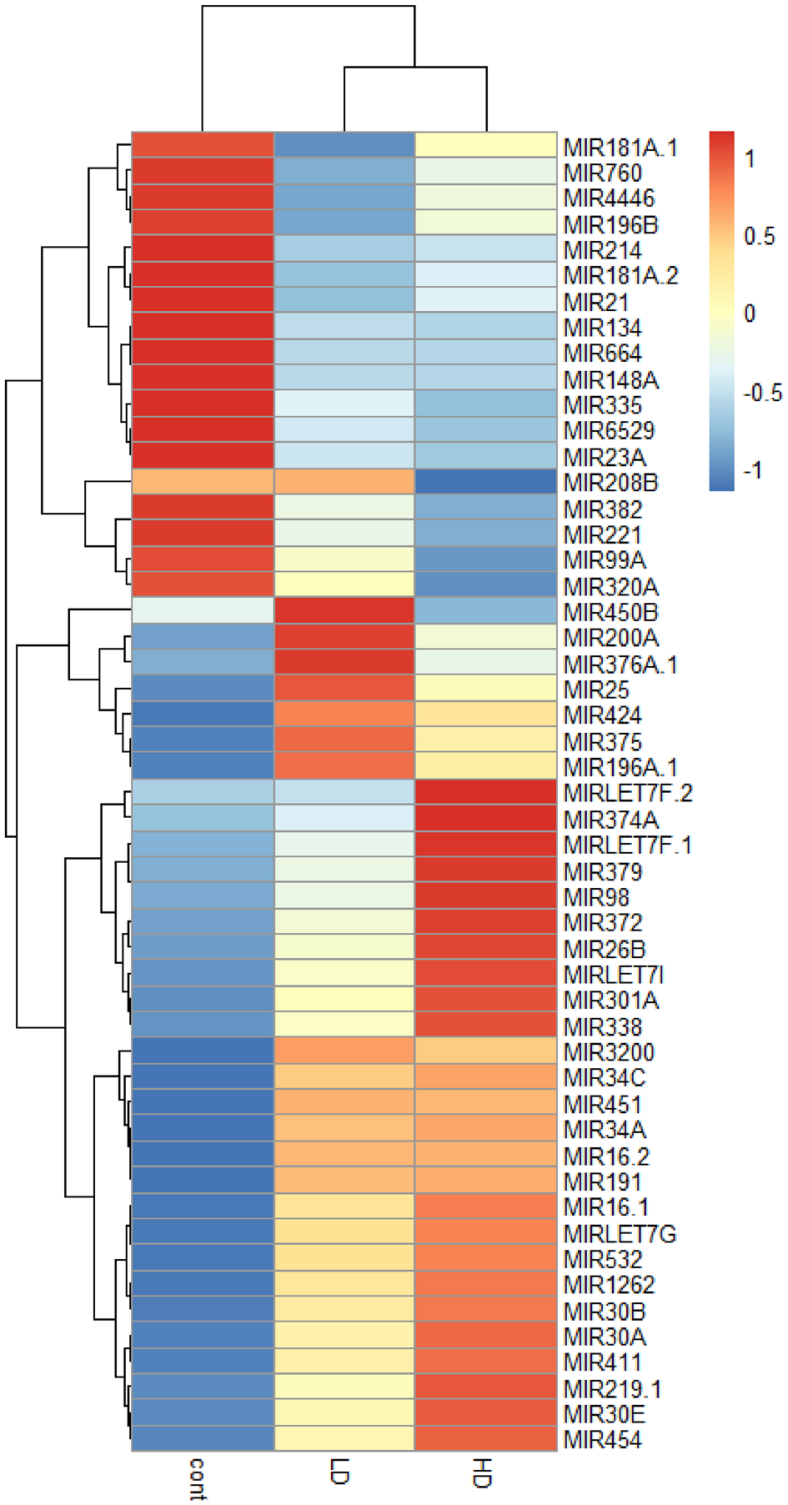
Heat map of miRNA features used to develop dose-prediction models at Days 3, 6, and 9, collapsed to show mean z-score for the three time points.

A Boruta random forest model was used to select important miRNAs, which were then narrowed down to the most important miRNAs for Days 3, 6, 9, 15, and 21. The profile plots of the top four miRNAs from all models are available upon request, which includes the miRNAs involved in each model. Days 6, 9, 15 and 21 all offered improved accuracies over the no-information rate (NIR) for differentiating between control, low dose, and high dose exposures. Table 2 displays the accuracies for the timepoints, with Day 15 offering a significant prediction accuracy of 81%, a significant improvement over the NIR of 45%. In addition, a prediction model was also built for Day 3, achieving an accuracy of 68%, however, unbalanced classifiers prevented complete statistics from being available. Figure 5 displays a heat map of the mean z-scores of miRNAs from Days 3, 6, and 9 for the dose prediction model.

### At Day 9, miRNAs predicted correct survival status with up to 71% accuracy

To develop a miRNA-based signature that could predict whether a given animal would die shortly after irradiation, at a later time point, or survive the length of the study, two sets of groupings were created. The first model included 3 groups: animals that died < 90 days (n = 11), animals that died from days 90–269 (n = 11), and animals that survived 270 days (n = 6). The second model included 4 groups: animals that died ≤ 60 days (n = 9), animals that died from 61 to 120 (n = 8), animals that died > 120 (n = 5), and animals that survived 270 days (n = 6). Using the Boruta random forest method to select predictive miRNAs, 9 markers were selected for the 3-group model and 8 markers were selected for the 4-group model. The accuracies and p-values for all days examined are shown in Table 2 and the confusion matrices and subsequent statistics are available for all days examined upon request.

### miRNA signatures predicted future onset of pleural effusion with high accuracy

To correlate miRNA expression with a manageable clinical manifestation of radiation damage, the future onset of pleural effusion was assessed at Day 3, 6, 9, 15, and 21, both by all members and then separately by gender, with the most predictive results occurring on Day 21 and Days 6 and 15 by gender. In this study, 17 out of 28 animals eventually developed pleural effusion. At Day 21, a model consisting of miR-665, miR-7–1, miR-324 and miR-181d predicted the later onset of pleural effusion with 100% accuracy (p-value = 0.003906). Additionally, Days 6 and 15 yielded accuracies of 87% and 75% by gender respectively, significant improvements over the NIRs of 37%. The accuracies and p-values for all days examined are shown in Table 2 and the confusion matrices and subsequent statistics are available for all days examined upon request.

### No significant pathway differences between animals immediately before death and survivors

**Figure 6:**
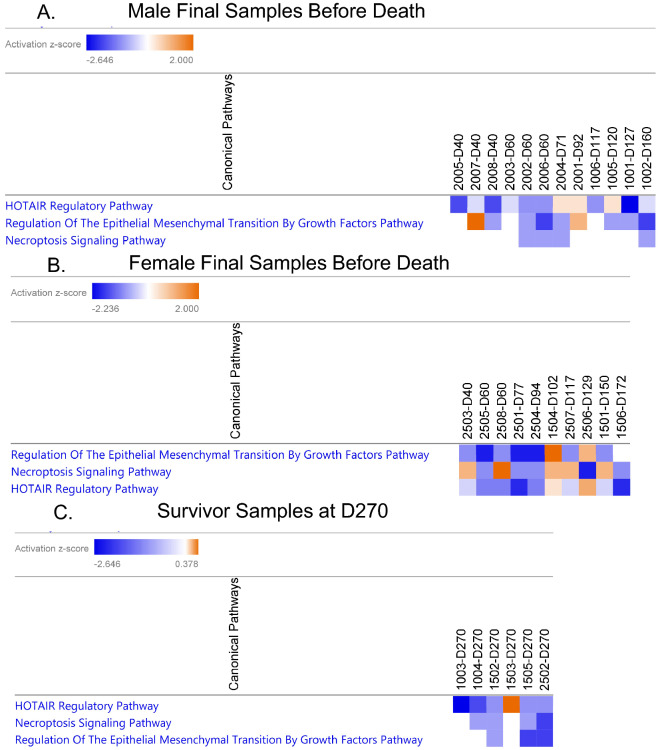
Pathway analysis via IPA for males at their latest time point sequenced before death (**A**), females at their latest time point sequenced before death (**B**) and surviving animals at Day 270 (**C**). A z-score absolute value cutoff of 0.1 was applied, with orange indicating upregulation and blue representing downregulation. Animals in 8A and 8B are listed in order of increasing length of survival.

Ingenuity Pathway Analysis (IPA) was used to elucidate pathway information from the miRNA expression data available. miRNA expression profiles from the latest sample sequenced for each non-surviving animal (n = 22) and the last sample for each surviving animal (Day 270, n = 6) were input with a z-score absolute value cutoff of 0.1. Figure 6A shows the pathways for each male sample, listed in order of increasing length of survival. Figure 6B shows the pathways for each female sample, listed in order of increasing length of survival. Figure 6C shows the pathways for the six surviving animals at Day 270.

### Pathway alteration after irradiation was time-dependent

**Figure 7:**
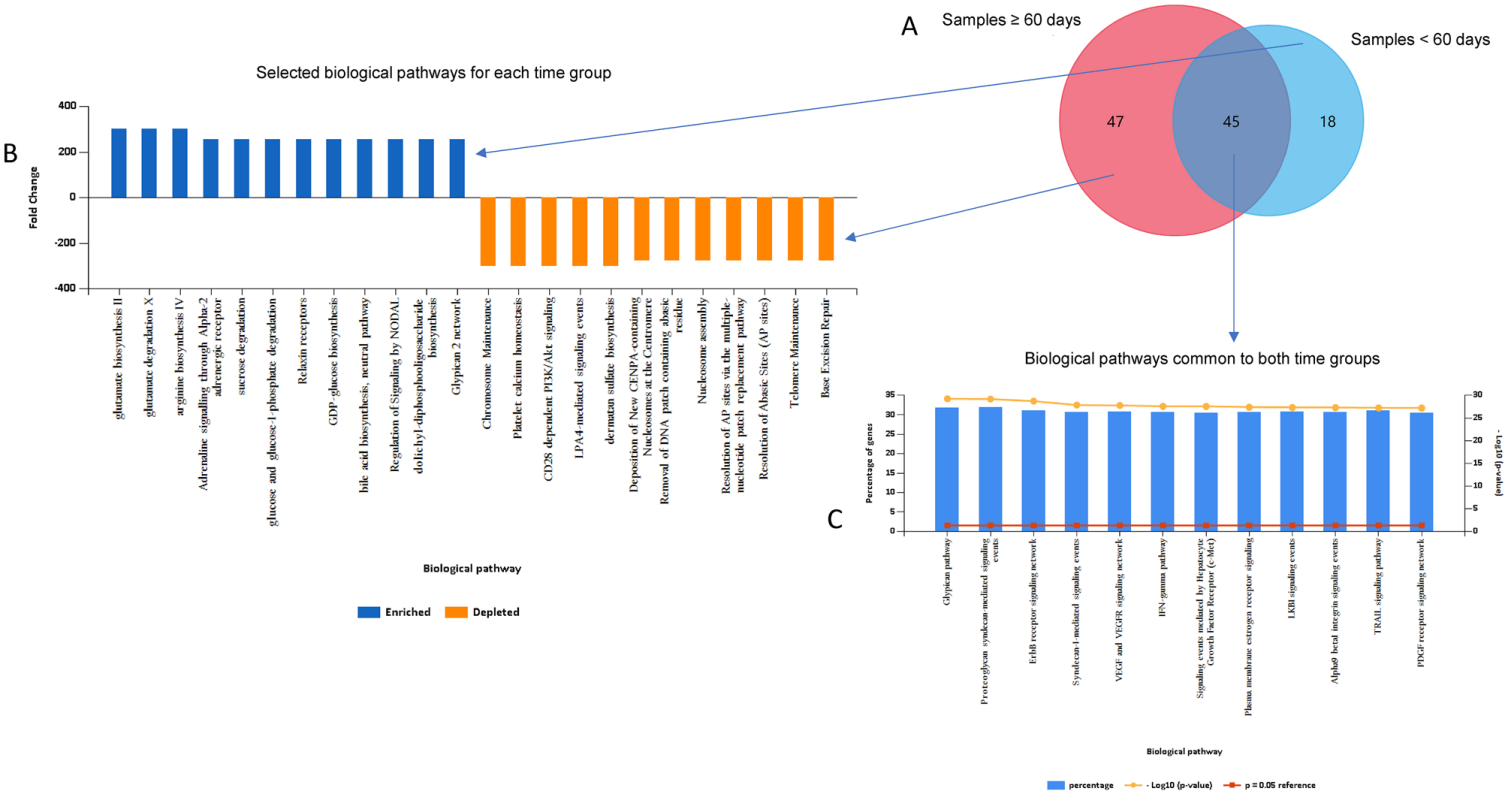
Significant miRNAs (adjusted p-value ≤ 0.05) between control samples vs. samples < 60 days and control samples vs samples  ≥ 60 days were tested for FunRich pathway enrichment analysis. Results were compared by (**A**) Venn overlaps showing the number of miRNA in each category (**B**) exclusive pathways for ≥ 60 days and < 60 days and (**C**) pathways common to both time groups.

FunRich pathway analysis of samples < 60 days and ≥ 60 days were compared to their control samples to determine which pathways were enriched or depleted in the acute or long-term phase of radiation damage. Figure 7A shows the Venn diagram of the number of significant miRNA (p-value ≤ 0.05) for each grouping and the number of miRNA altered in common. Figure 7B displays the pathways that were enriched or depleted for samples < 60 days and ≥ 60 days. Figure 7C demonstrates the pathways related to the 45 miRNAs that were common to the samples greater than and less than 60 days. Additional FunRich pathway comparisons are available in Supplemental Figs. 3–6, including comparing females before and after 60 days (Supplemental Fig. 3), comparing males before and after 60 days (Supplemental Fig. 4), comparing males and females ≥ 60 days (Supplemental Fig. 5), and comparing males and females < 60 days (Supplemental Fig. 6).

## Discussion

Although NHPs have been used for various studies on radiation damage in the past^33–41^, this is the first study to report on significant expression changes in serum miRNA up to 270 days after irradiation, giving a comprehensive understanding of long-term miRNA expression changes. The duration of this study, focus on dose-dependent survival differences, and analysis of pathways affected by radiation damage make this study unique. For the two whole thorax doses utilized in this study, 9.8 and 10.7 Gy, which corresponded to LD_20/180_ and LD_75/180_, respectively, the predicted lethal dose did not match the observed survival outcomes. At 180 days, 7 out of 12 low dose animals succumbed to their injuries and 15 out of 16 high dose animals succumbed to their injuries, bringing the lethal doses to LD_58.33/180_ and LD_93.75/180_, respectively, for 9.8 and 10.7 Gy.

Given the results of clinical examinations, CT imaging, and necropsy results, these animals likely suffered from effects of DEARE. First, an increase in respiratory rate around days 40–45 and a subsequent decrease in oxygen saturation indicate delayed lung damage, a primary concern of DEARE. Second, CT scans revealed radiation-induced pneumonitis in 86% of animals, with only four animals not showing evidence of pneumonitis. These four animals, all males in the high dose group, likely died before the pathology could appear on CT imaging. In a previous NHP study using 10 and 11 Gy partial body irradiation, the first evidence of pneumonitis had a range of 100–107 days post-irradiation^4^, so it is likely the animals in this study succumbed to other injuries prior to development. Additionally, most of the animals displayed respiratory distress, including all animals that died before the end of the study in the low dose group. Upon necropsy of lung and heart tissue of animals that died before the end of the study and animals humanely euthanized at the conclusion of the study, the average severity scores were higher for animals that died compared to animals that survived, though the difference was only significant in heart tissue (Table 1).

The number of significant miRNAs expressed by each of the four groups at each time point sampled is shown in Fig. 2. In general, there was at least one significant miRNA expressed at each time point for each group, demonstrating the long-term dysregulation of miRNA in NHPs after irradiation. Similarly, Supplemental Table 2 demonstrates that there were appreciable numbers of miRNA that were significantly different between doses for each gender, demonstrating the dose-dependent response of some miRNA. Additionally, Day 21 showed either a local maxima or global maxima for number of significant miRNAs for all four groups. While the radiation biodosimetry paradigm has long been to test in the optimal treatment window (days 1–7 post-irradiation)^42^, the results of this study may offer support for more biological perturbations at later time points. Although early testing may remain the staple to begin necessary treatment as soon as possible, retesting patients at later time points may enhance the quality of information available and provide further diagnostics. These observations indicate the damage sustained to the heart and lungs, the primary organs in the field of radiation in this study, as well as the delayed manifestations characteristic of DEARE.

Two-way ANOVA revealed that there are 76 unique miRNAs which are differentially expressed by all four groups at any time point. The heat map in Fig. 2 displays these 76 miRNAs. Despite being common to both genders, notably these miRNAs still segregated by gender and time, meaning that their time course of expression was different between males and females. Although there were no survival differences between genders, these results may point to radiation response differences between genders. In the time course expression of the 76 miRNAs of Supplemental Fig. 2, the expression levels of miRNAs fluctuate significantly over the 270-day study. For example, miR-95 begins at Day 3 with a slight upregulation for both doses, followed by a downregulation from Days 6–15, and then an upregulation at Day 21, continuing to fluctuate for the remaining time points. These results demonstrate the importance of time in determining biomarkers for radiation damage due to the complex radiation response of higher order, heterogenous species. In a real scenario, miRNA biomarker assays may need to be specific to the time of sampling, with a specific panel of markers for each possible sampling period.

Comparing the 21 miRNAs common to both genders and both doses at any time point with the 26 miRNAs that displayed dose-dependent expression, there are five miRNAs that overlap. All five miRNAs (miR-26b, miR-34a, miR-30a, miR-34c, and miR-454) are previously reported in the literature as an elevated marker of radiation damage or promoted cell survival after irradiation. In a study of workers that assisted with the Chernobyl disaster clean-up, the increased expression of hsa-miR-26b-5p (the 5’ arm transcript of miR-26b) was associated with radiation-induced breast cancer^43^.This upregulation in tissue exposed to radiation, which later became cancerous, is of interest due to the implications of having miRNA, including miR-26b, upregulated long term.

Previous reports showed miR-34a was upregulated in total-body irradiated C3H mice and was part of a signature that best forecasted 120-day survival within the first 30 days^27^. In lymphoblastoid TK6 cells, the upregulation of miR-34a was observed after treatment with genotoxic agents, including x-ray irradiation^44^. These studies demonstrate the involvement in DNA damage response by miR-34a and the utility as a marker of radiation damage and coincides with the upregulation we reported. miR-30a has been reported in several radiation biomarker studies, including a meta-analysis which found miR-30a had an average fold change of 1.18 over six studies utilizing NHPs, humans, and mice^45^, demonstrating the correlation of the miRNA with radiation response and the conservation in response between species. miR-34c was previously shown to be induced by radiation in extracellular vesicles (EVs) and involved with the generation of reactive oxygen species^46^. This study is of interest due to the presence of EVs in serum, which corroborates our results. miR-454, which targets AKT, was shown to promote proliferation and improve survival of triple negative breast cancer cells after irradiation^47^. Upregulated in triple negative breast cancer cells^47^, this study is of interest for the upregulation we also observed in previously healthy NHPs long-term after irradiation and the link between NHP survival and breast cancer cell proliferation.

Given that this study only utilized two different whole thorax doses of radiation, the application of our dose-prediction model is limited. However, several important conclusions can be drawn. Despite a small sample size given the constraints of large animal models, our prediction model at Day 15 still achieved an accuracy of 81%, greater than the no-information rate (NIR), the accuracy of picking the majority class every time when given no other information. The accuracy of 81% at Day 15 offers a significant improvement over the NIR of 45%. Additionally, the Kappa statistic values of 0.7179 for Day 15 indicates substantial agreement of categorical data, according to Landis and Koch^48^. The first 21 days were selected for testing because they represent some of the most likely timepoints for sampling mass populations after a radiological incident. While rapid tests may also be extremely valuable (within first 48 h)^1^, the potential for significant damage to healthcare systems, need for relocation of supplies, and large numbers of tests required may result in significant delays, making tests after the first few days necessary. Although only two doses were used, the accuracy of this model offers an improvement over a partial-body dose prediction model previously reported using plasma proteins in mice^49^.

As shown in the time course profiles of Supplemental Fig. 2, the expression level of miRNA over time is variable, especially in a heterogenous population, necessitating time-specific testing. From the dose-prediction models, one miRNA, miR-34a, has been previously reported as dysregulated following irradiation. In a human prostate cancer cell model, our group previously reported the upregulation of miR-34a after fractionated radiation^50^, which coincides with the upregulation observed in NHPs in this study.

The results of the death-prediction model offer the first instance of forecasting death by miRNA signature after irradiation in a large animal model over a long-term, comprehensive time frame. We have previously reported a death-prediction model in Gottingen minipigs over a 45-day interval after exposure to 1.7–2.3 Gy TBI, using gene expression from heart, lung and liver tissue separately^51^. This study builds upon the previous work by utilizing a closer genetic relative to humans, following animals over a longer period, and using easily attainable serum samples. In our 3-group model at Day 9, 71% accuracy was achieved in classifying the survival status, a significant improvement over the NIR of 47%, as evidenced by the p-value < 0.05. The Kappa statistic of 0.4444 indicates moderate categorical agreement^48^. In the 4-group model at Day 3, 62.5% accuracy was achieved in classifying survival status, a modest improvement over the NIR of 37%, with a kappa statistic classifying the model as moderate categorical agreement^48^. Similar analyses were also performed for Days 6, 15, and 21, however these yielded less accurate models than for Days 3 and 9. Due to limits in sample size, we were unable to classify animals by gender, which would have resulted in classifications too small for analysis (n ≤ 3). Understanding when an animal, or a human in a catastrophic scenario, may succumb to their injuries will allow clinicians to prioritize scarce medical resources to those most likely to survive.

While forecasting death is an important endpoint, in a mass casualty scenario it is also important to predict the onset of a clinically manageable symptom at delayed time points. Pneumonitis is a well-known clinical manifestation of DEARE, typically occurring 60-plus days after exposure to ionizing radiation to the thorax. In this study, all but four animals developed pneumonitis evidence on CT scan, with the four not developing pneumonitis succumbing to their injuries in less than 60 days, before most other animals developed the condition. Because of this, the onset of pleural effusion, a previously reported effect of radiation in NHPs^52^ was instead chosen as a clinical manifestation to examine. In this study, 61% of animals developed pleural effusion. At Day 21, the 100% accurate prediction of future onset of pleural effusion was significant (p-value = 0.003906) with a Kappa statistic of 1, indicating almost perfect categorical agreement. Additionally, the accuracies of 87% and 75% for developing pleural effusion by gender provide alternate days for testing, with the ability to also correctly predict the gender of the animal sampled. Knowledge of future onset of pleural effusion will allow clinicians to proactively, rather than reactively, monitor patients for related symptoms, which may prolong life.

Pathway analysis using IPA was performed to determine if the exposed NHPs expressed any similar pathways immediately prior to radiation-induced death, and if these differed from pathways expressed by NHPs that survived the length of the study. Despite differences in gender and survival, all three groupings in Fig. 6 (male decedents, female decedents, and survivors) dysregulated the same three pathways: HOTAIR regulatory pathway, necroptosis signaling pathway, and regulation of epithelial mesenchymal transition (EMT) by growth factors pathway. In general, these pathways expressed mild to moderate downregulation, however some NHPs expressed an induction of these pathways, such as animal 2001 at Day 92 (Fig. 6A) and animal 1504 at Day 102 (Fig. 6B). Additionally, Fig. 6B suggests that longer-surviving females tended to be more likely to express upregulation of these three pathways than their shorter-surviving counterparts. In surviving animals (Fig. 6C), five out of six displayed a downregulation of HOTAIR pathway, with only one animal, 1503, expressing a slight, but significant, upregulation in the pathway (z-score = 0.378, p-value = 0.000148). *HOTAIR*, the HOX antisense intergenic lncRNA, is known to function in normal cell development, and the overexpression of *HOTAIR* leads to breast cancer development^53^. Likewise, the knockdown of *HOTAIR* in HeLa cells has been shown to inhibit autophagy via suppressing the Wnt signaling pathway^54,55^. This points to the hypothesis that the downregulation of the HOTAIR pathway by the NHPs in this study may be an attempt to prolong survival. Similarly, HOTAIR pathway and Wnt signaling are linked to EMT^55^ explaining the congruent downregulation of these two pathways. Although necroptosis and autophagy are both methods of programmed cell death, the link between the two is not well-defined. Goodall et al. have proposed that autophagy machinery scaffolds for necrosome complex formation^56^, connecting the two pathways.

FunRich analysis in Fig. 7 demonstrated that there are both unique and conserved pathways between the acute reaction to radiation damage and the long-term dysfunction of the animals. In Fig. 7B, it is evident that within the first 60 days after irradiation, the primary pathways altered are metabolic. This is evident by the enrichment in glutamate biosynthesis and degradation, arginine biosynthesis, glucose and sucrose degradation, and bile acid biosynthesis. This contrasts with the pathways altered after day 60, which tended to be depletion of repair and maintenance, such as chromosome maintenance, nucleosome assembly, resolution of apurinic/apyrimidinic sites (AP) sites, telomere maintenance, and base excision repair. Distinct acute and chronic phases post-irradiation were previously demonstrated in non-small cell lung cancer patients undergoing radiotherapy^57^. Alternatively, many signaling pathways were significantly altered between both groups and have been linked to radiation damage and/or cellular response to cancer. Interferon-gamma has been shown to protect against tumor development^58^. ErbB receptor signaling and overexpression has been linked to poor cancer prognosis and radiation resistance through activation of MAPK^59,60^ and PDGF receptor signaling has been shown to promote radioresistance in human prostate cancer cells^61^.

In this study, we have identified key radiation-induced miRNA in NHPs up to 270 days after whole thorax irradiation. We found no significant survival differences between male and female NHPs, though future studies should corroborate this finding due to the limited sample size. We have highlighted the variability of miRNAs in NHPs over long periods of time, both in the number of significantly expressed markers and the expression profiles of individual markers. This study also revealed several key differences and similarities between the miRNA expression patterns of male and female NHPs. A total of 26 miRNAs were shown to be dose-dependent at all time points combined, including 16 downregulated and 10 upregulated. Through pathway analysis comparing survivors and decedents, we have shown that little differences exist in dysregulated pathways, with HOTAIR regulatory pathway, necroptosis signaling, and EMT by growth factors being commonly dysregulated. Notably, the miRNA signature models developed could be used to test dose, onset of pleural effusion, and forecast survival/death within the first nine days. We acknowledge the limitation of the study that validation using an independent cohort of irradiated animals is warranted; the results presented here shine light on possible mechanisms and markers and provide valuable insights which could be investigated further in the future. In a clinical setting following a mass radiation exposure, the ability to determine dose received and forecast death at early timepoints can inform medical providers who should receive medical countermeasures and of which kind, which will mitigate normal tissue injury and conserve valuable resources.

## Supplementary Information

Supplementary Information 1.

Supplementary Information 2.

Supplementary Information 3.

Supplementary Information 4.

Supplementary Information 5.

Supplementary Information 6.

Supplementary Information 7.

Supplementary Information 8.

Supplementary Information 9.

Supplementary Information 10.

Supplementary Information 11.

## References

[1] Coleman C, Koerner JF (2016). Biodosimetry: Medicine, science, and systems to support the medical decision-maker following a large scale nuclear or radiation incident. Radiat. Prot. Dosim..

[2] López M, Martín M (2011). Medical management of the acute radiation syndrome. Rep. Pract. Oncol. Radiother..

[3] Giuranno L, Ient J, De RD, Vooijs MA (2019). Radiation-induced lung injury (RILI). Front. Oncol..

[4] MacVittie TJ, Farese AM, Parker GA, Jackson W (2019). The time course of radiation-induced lung injury in a nonhuman primate model of partial-body irradiation with minimal bone marrow sparing: Clinical and radiographic evidence and the effect of neupogen administration. Health Phys..

[5] Parker GA, Li N, Takayama K, Farese AM, MacVittie TJ (2019). Lung and heart injury in a nonhuman primate model of partial-body irradiation with minimal bone marrow sparing: Histopathological evidence of lung and heart injury. Health Phys..

[6] Sproull M, Camphausen K (2016). State-of-the-art advances in radiation biodosimetry for mass casualty events involving radiation exposure. Radiat Res..

[7] Dörr H, Abend M, Blakely WF, Bolduc DL, Boozer D, Costeira T (2017). Using clinical signs and symptoms for medical management of radiation casualties-2015 NATO exercise. Radiat. Res..

[8] Demidenko E, Williams BB, Swartz HM (2009). Radiation dose prediction using data on time to emesis in the case of nuclear terrorism. Radiat. Res..

[9] De Lemos Pinto MMP, Santos NFG, Amaral A (2010). Current status of biodosimetry based on standard cytogenetic methods. Radiat. Environ. Biophys..

[10] Prasanna PGS, Moroni M, Pellmar TC (2010). Triage dose assessment for partial-body exposure: Dicentric analysis. Health Phys..

[11] Waselenko JK, MacVittie TJ, Blakely WF, Pesik N, Wiley AL, Dickerson WE (2004). Medical management of the acute radiation syndrome: Recommendations of the Strategic National Stockpile Radiation Working Group. Ann. Intern. Med..

[12] Mendell JT, Olson EN (2012). MicroRNAs in stress signaling and human disease. Cell.

[13] Cortez MA, Bueso-Ramos C, Ferdin J, Lopez-Berestein G, Sood AK, Calin GA (2011). MicroRNAs in body fluids-the mix of hormones and biomarkers. Nat. Rev. Clin. Oncol..

[14] Templin T, Amundson SA, Brenner DJ, Smilenov LB (2011). Whole mouse blood microRNA as biomarkers for exposure to γ-rays and 56Fe ions. Int. J. Radiat. Biol..

[15] Cui W, Ma J, Wang Y, Biswal S (2011). Plasma miRNA as biomarkers for assessment of total-body radiation exposure dosimetry. PLoS ONE.

[16] Jacob NK, Cooley JV, Yee TN, Jacob J, Alder H, Wickramasinghe P (2013). Identification of sensitive serum microRNA biomarkers for radiation biodosimetry. PLoS ONE.

[17] Tomasik B, Fendler W, Chowdhury D (2018). Serum microRNAs: Potent biomarkers for radiation biodosimetry. Oncotarget.

[18] Aryankalayil MJ, Chopra S, Makinde A, Eke I, Levin J, Shankavaram U (2018). Microarray analysis of miRNA expression profiles following whole body irradiation in a mouse model. Biomarkers.

[19] Menon N, Rogers CJ, Lukaszewicz AI, Axtelle J, Yadav M, Song F (2016). Detection of acute radiation sickness: A feasibility study in non-human primates circulating miRNAs for triage in radiological events. PLoS ONE.

[20] Fleckenstein K, Gauter-Fleckenstein B, Jackson IL, Rabbani Z, Anscher M, Vujaskovic Z (2007). Using biological markers to predict risk of radiation injury. Semin. Radiat. Oncol..

[21] Simone CB (2017). Thoracic radiation normal tissue injury. Semin. Radiat. Oncol..

[22] Kong FM, Ao X, Wang L, Lawrence TS (2008). The use of blood biomarkers to predict radiation lung toxicity: A potential strategy to individualize thoracic radiation therapy. Cancer Control.

[23] Liu Z, Liang X, Li X, Liu X, Zhu M, Gu Y (2019). MiRNA-21 functions in ionizing radiation-induced epithelium-to-mesenchymal transition (EMT) by downregulating PTEN. Toxicol. Res. (Camb.)..

[24] Wang D, Liu Z, Yan Z, Liang X, Liu X, Liu Y (2019). MiRNA-155–5p inhibits epithelium-to-mesenchymal transition (EMT) by targeting GSK-3β during radiation-induced pulmonary fibrosis. Arch. Biochem. Biophys..

[25] Lei X, He N, Zhu L, Zhou M, Zhang K, Wang C (2020). Mesenchymal stem cell-derived extracellular vesicles attenuate radiation-induced lung injury via miRNA-214-3p. Antioxid. Redox Signal..

[26] Stewart JR, Fajardo LF, Gillette SM, Constine LS (1995). Radiation injury to the heart. Int. J. Radiat. Oncol. Biol. Phys..

[27] Rogers CJ, Lukaszewicz AI, Yamada-Hanff J, Micewicz ED, Ratikan JA, Starbird MA (2020). Identification of miRNA signatures associated with radiation-induced late lung injury in mice. PLoS ONE.

[28] Singh VK, Olabisi AO (2017). Nonhuman primates as models for the discovery and development of radiation countermeasures. Expert Opin. Drug Discov..

[29] R Core Team R (2021). A language and environment for statistical computing.

[30] Ritchie ME, Phipson B, Wu D, Hu Y, Law CW, Shi W (2015). Limma powers differential expression analyses for RNA-sequencing and microarray studies. Nucleic Acids Res..

[31] Kursa MB, Rudnicki WR (2010). Feature selection with the Boruta package. J. Stat. Softw..

[32] Pathan M, Keerthikumar S, Chisanga D, Alessandro R, Ang C-S, Askenase P (2017). A novel community driven software for functional enrichment analysis of extracellular vesicles data. J. Extracell Vesicles.

[33] Li Y, Singh J, Varghese R, Zhang Y, Fatanmi OO, Cheema AK (2021). Transcriptome of rhesus macaque (*Macaca mulatta*) exposed to total-body irradiation. Sci. Rep..

[34] Thakur P, DeBo R, Dugan GO, Bourland JD, Michalson KT, Olson JD, Register TC, Kock ND, Cline JM (2021). Clinicopathologic and Transcriptomic Analysis of Radiation-Induced Lung Injury in Nonhuman Primates. Int. J. Radiat. Oncol. Biol. Phys.

[35] Cui W, Hankey KG, Zhang P, Bolduc DL, Bünger R, Xiao M (2020). Identifying circulating and lung tissue cytokines associated with thoracic irradiation and AEOL 10150 treatment in a nonhuman primate model. Radiat. Res..

[36] Xiao M, Bolduc DL, Li X, Cui W, Hieber KP, Bünger R (2017). Urine interleukin-18 (IL-18) as a biomarker of total-body irradiation: A preliminary study in nonhuman primates. Radiat. Res..

[37] Balog RP, Bacher R, Chang P, Greenstein M, Jammalamadaka S, Javitz H (2020). Development of a biodosimeter for radiation triage using novel blood protein biomarker panels in humans and non-human primates. Int. J. Radiat. Biol..

[38] Balog RP, Chang P, Javitz HS, Lee S, Lin H, Shaler T (2020). Development of a point-of-care radiation biodosimeter: Studies using novel protein biomarker panels in non-human primates. Int. J. Radiat. Biol..

[39] Iversen ES, McCarthy JM, Burdett KB, Lipton G, Phillips G, Dressman H (2020). Bridging the gaps: Using an NHP model to predict single dose radiation absorption in humans. Int. J. Radiat. Biol..

[40] Pannkuk EL, Laiakis EC, Mak TD, Astarita G, Authier S, Wong K (2016). A lipidomic and metabolomic serum signature from nonhuman primates exposed to ionizing radiation. Metabolomics.

[41] Ha CT, Li XH, Fu D, Moroni M, Fisher C, Arnott R (2014). Circulating interleukin-18 as a biomarker of total-body radiation exposure in mice, minipigs, and nonhuman primates (NHP). PLoS ONE.

[42] Amundson SA. Transcriptomics for radiation biodosimetry: Progress and challenges. *Int. J. Radiat. Biol*. 2021. Online ahead of print 10.1080/09553002.2021.1928784 PMC10026363 33970766

[43] Wilke CM, Hess J, Klymenko SV, Chumak VV, Zakhartseva LM, Bakhanova EV (2018). Expression of miRNA-26b-5p and its target TRPS1 is associated with radiation exposure in post-Chernobyl breast cancer. Int. J. Cancer.

[44] Chen T, Yan J, Li Z (2020). Expression of miR-34a is a sensitive biomarker for exposure to genotoxic agents in human lymphoblastoid TK6 cells. Mutat. Res. Genet. Toxicol. Environ. Mutagen..

[45] Małachowska B, Tomasik B, Stawiski K, Kulkarni S, Guha C, Chowdhury D (2020). Circulating microRNAs as biomarkers of radiation exposure: A systematic review and meta-analysis. Int. J. Radiat. Oncol. Biol. Phys..

[46] Rastogi S, Hwang A, Chan J, Wang JYJ (2018). Extracellular vesicles transfer nuclear Abl-dependent and radiation-induced miR-34c into unirradiated cells to cause bystander effects. Mol. Biol. Cell.

[47] Li Q, Liu J, Meng X, Pang R, Li J (2017). MicroRNA-454 may function as an oncogene via targeting AKT in triple negative breast cancer. J. Biol. Res..

[48] Landis JR, Koch GG (1977). The measurement of observer agreement for categorical data. Biometrics.

[49] Sproull M, Kramp T, Tandle A, Shankavaram U, Camphausen K (2017). Multivariate analysis of radiation responsive proteins to predict radiation exposure in total-body irradiation and partial-body irradiation models. Radiat. Res..

[50] John-Aryankalayil M, Palayoor ST, Makinde AY, Cerna D, Simone CB (2012). Fractionated radiation alters oncomir and tumor suppressor miRNAs in human prostate cancer cells. Radiat. Res..

[51] Chopra S, Moroni M, Martello S, Bylicky M, May J, Hritzo B (2020). Gene expression profiles from heart, lung and liver samples of total-body-irradiated minipigs: Implications for predicting radiation-induced tissue toxicity. Radiat. Res.

[52] Macvittie TJ, Gibbs A, Farese AM, Barrow K, Bennett A, Taylor-Howell C (2017). AEOL 10150 mitigates radiation-induced lung injury in the nonhuman primate: Morbidity and mortality are administration schedule-dependent. Radiat. Res..

[53] Mozdarani H, Ezzatizadeh V, Rahbar PR (2020). The emerging role of the long non-coding RNA HOTAIR in breast cancer development and treatment. J. Transl. Med..

[54] Podralska M, Ciesielska S, Kluiver J, van den Berg A, Dzikiewicz-Krawczyk A, Slezak-Prochazka I (2020). Non-coding RNAs in cancer radiosensitivity: MicroRNAs and lncrnas as regulators of radiation-induced signaling pathways. Cancers (Basel).

[55] Guo X, Xiao H, Guo S, Li J, Wang Y, Chen J (2019). Long noncoding RNA HOTAIR knockdown inhibits autophagy and epithelial–mesenchymal transition through the Wnt signaling pathway in radioresistant human cervical cancer HeLa cells. J. Cell Physiol..

[56] Goodall ML, Fitzwalter BE, Zahedi S, Wu M, Rodriguez D, Mulcahy-Levy JM (2016). The autophagy machinery controls cell death switching between apoptosis and necroptosis. Dev. Cell..

[57] Śliwińska-Mossoń M, Wadowska K, Trembecki L, Bil-Lula I (2020). Markers useful in monitoring radiation-induced lung injury in lung cancer patients: A review. J. Pers. Med..

[58] Ikeda H, Old L, Schreiber R (2002). The roles of IFN gamma in protection against tumor development and cancer immunoediting. Cytokine Growth Factor Rev..

[59] Wang Z (2017). ErbB receptors and cancer. Methods Mol. Biol..

[60] Schmidt-Ullrich RK, Contessa JN, Lammering G, Amorino G, Lin P-S (2003). ERBB receptor tyrosine kinases and cellular radiation responses. Oncogene.

[61] Paximadis P, Najy A, Snyder M, Kim H (2016). The interaction between androgen receptor and PDGF-D in the radiation response of prostate carcinoma. Prostate.

