# Supplementary material for: Serum RNA biomarkers for predicting survival in non-human primates following thoracic radiation
Source: Sci Rep. 2022 Jul 19;12:12333. doi: 10.1038/s41598-022-16316-x (PMC9296457; doi:10.1038/s41598-022-16316-x)
Supplement: Supplementary file 1 — Supplementary Information 1. [file 41598_2022_16316_MOESM1_ESM.docx]

**Supplemental Legends**

*Supplemental Table 1:* Number of serum samples analyzed at each timepoint for each group. Table includes terminal samples taken between formal timepoints due to early euthanasia.

*Supplemental Table 2:* Number of miRNAs common to both doses for each gender at listed time points

Supplemental Table 3: List of miRNA identities which are at the intersections of Figure 3, including miRNAs expressed in either both genders or both doses based on results from one-way ANOVA (omnibus, p ≤ 0.05), by dose and by gender.

*Supplemental Table 4:* List of dose-dependent miRNA for all timepoints and both genders. The 16 dose-dependent downregulated miRNAs are highlighted in blue, and the 10 dose-dependent upregulated miRNAs are highlighted in red. Mean values, p-values, and fold changes of the dose-dependent miRNAs are included.

*Supplemental Figure 1:* qRT-PCR plots for few differentially expressed miRNAs chosen for validation. qRT-PCR was performed using miRCURY LNA RT and SYBR Green PCR reagents for miR-454-3p, let-7c-5p, 34a-5p, miR-26b-5p, let-7g-5p and miR-17-5p. Y-axis represents fold change in D3 and D6 compared to control values. Control fold change values were calculated from the average CT values across all controls. Outlier samples were excluded from some graphs based on Grubbs test (when G-statistical value exceeded G critical value).

*Supplemental Figure 2*: Profile plots of the 76 miRNAs expressed by both genders and both doses at any time point (p ≤ 0.05) by two-way top-level ANOVA.

*Supplemental Figure 3:* FunRich pathway enrichment analysis based on significant miRNAs (adjusted p-value ≤ 0.05) between female samples ≥ 60 days vs female samples < 60 days. Results were compared by A) Venn overlaps showing the number of miRNA in each category B) exclusive pathways for ≥ 60 days and < 60 days and C) pathways common to both female groups.

*Supplemental Figure 4:* FunRich pathway enrichment analysis based on significant miRNAs (adjusted p-value ≤ 0.05) between male samples ≥ 60 days vs male samples < 60 days. Results were compared by A) Venn overlaps showing the number of miRNA in each category B) exclusive pathways for ≥ 60 days and < 60 days and C) pathways common to both male groups.

*Supplemental Figure 5:* FunRich pathway enrichment analysis based on significant miRNAs (adjusted p-value ≤ 0.05) between male samples ≥ 60 days vs female samples ≥60 days. Results were compared by A) Venn overlaps showing the number of miRNA in each category B) exclusive pathways for male samples ≥ 60 days and female samples ≥ 60 days and C) pathways common to both time points.

*Supplemental Figure 6:* FunRich pathway enrichment analysis based on significant miRNAs (adjusted p-value ≤ 0.05) between male samples < 60 days vs female samples < 60 days. Results were compared by A) Venn overlaps showing the number of miRNA in each category B) exclusive pathways for male samples < 60 days and female < 60 days and C) pathways common to both time points.
