# Supplementary figures and images for: Serum RNA biomarkers for predicting survival in non-human primates following thoracic radiation
Source: Sci Rep. 2022 Jul 19;12:12333. doi: 10.1038/s41598-022-16316-x (PMC9296457; doi:10.1038/s41598-022-16316-x)

## Slide 1
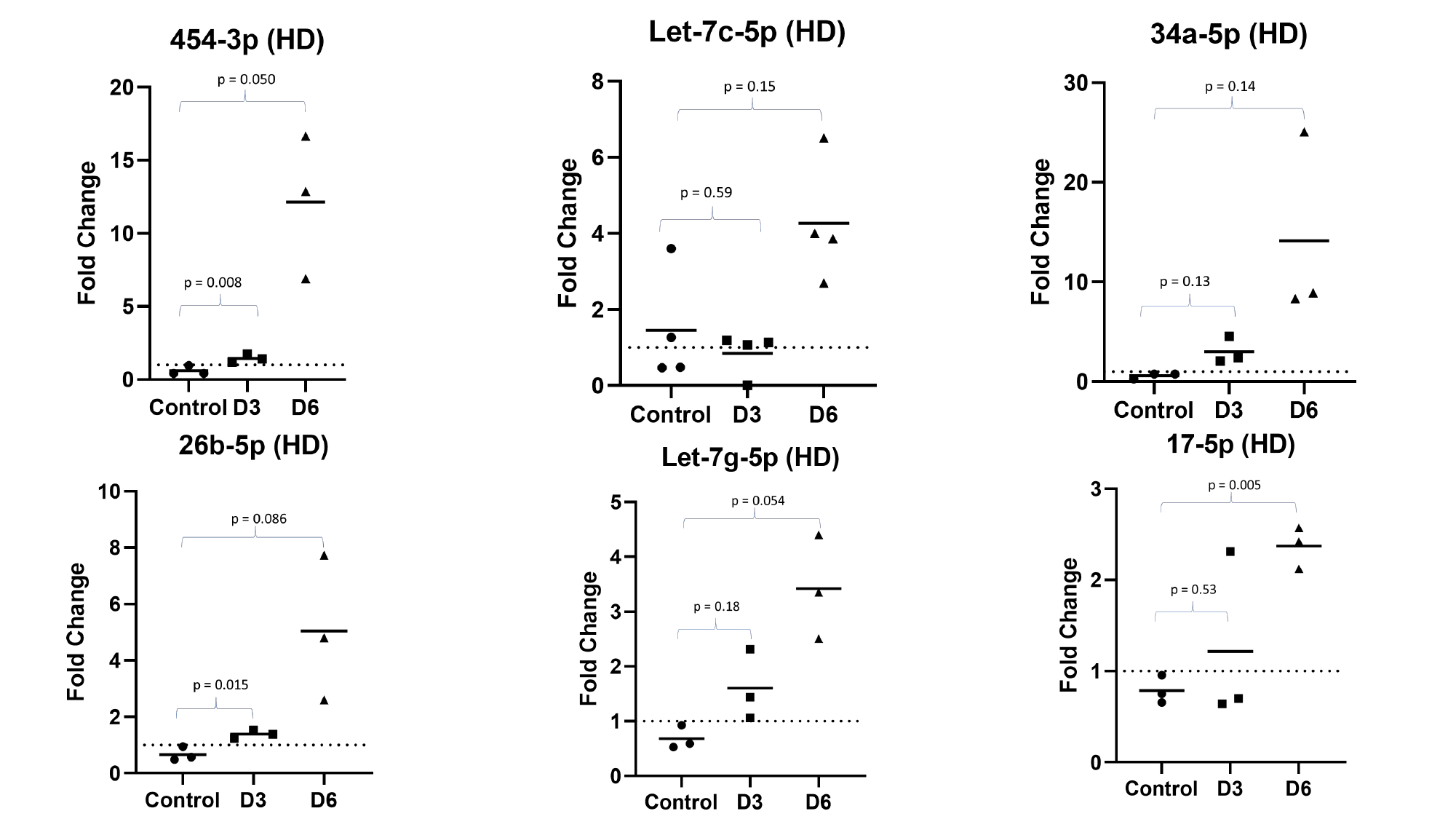

Supplement: Supplementary file 2 — Supplementary Information 2. [file 41598_2022_16316_MOESM2_ESM.pptx]

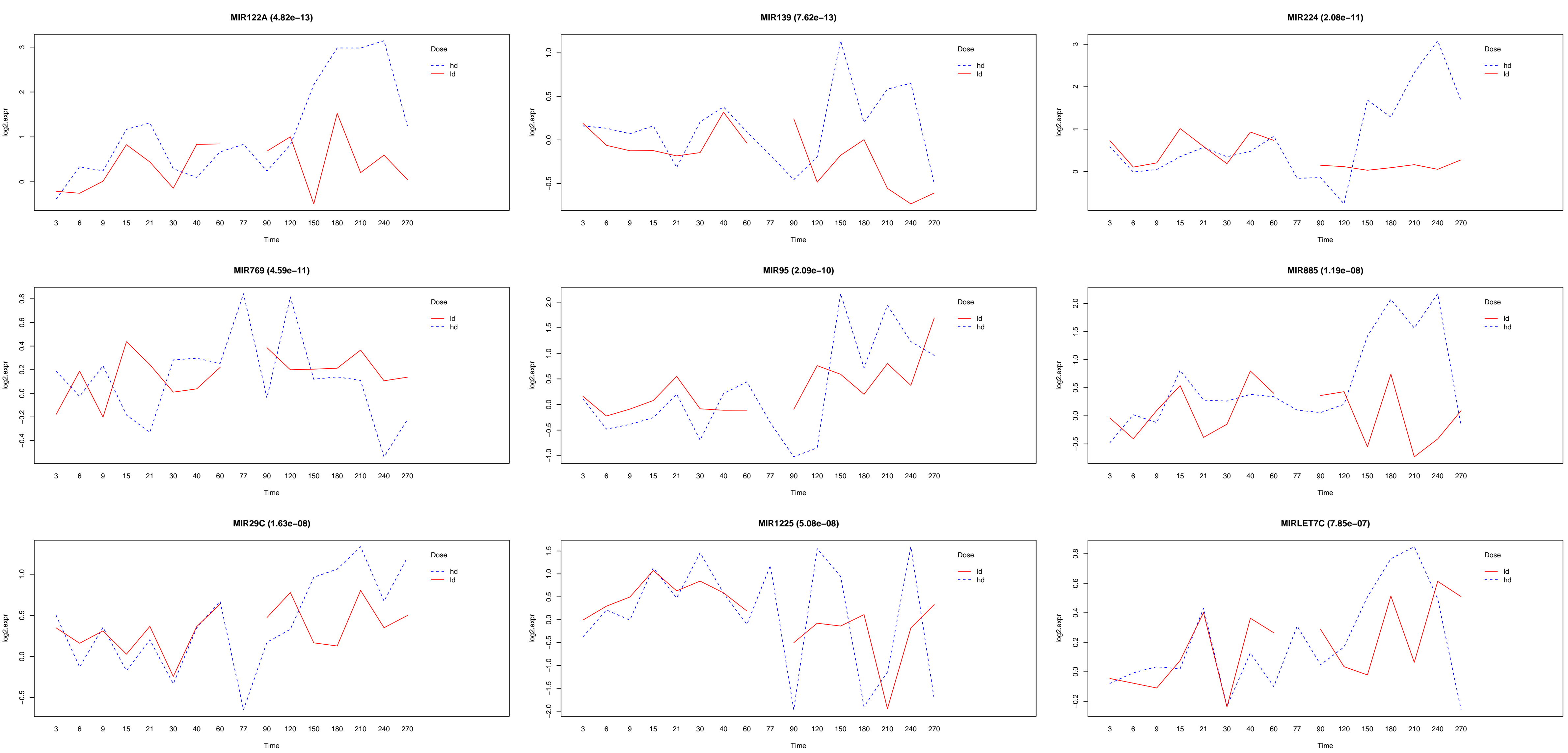

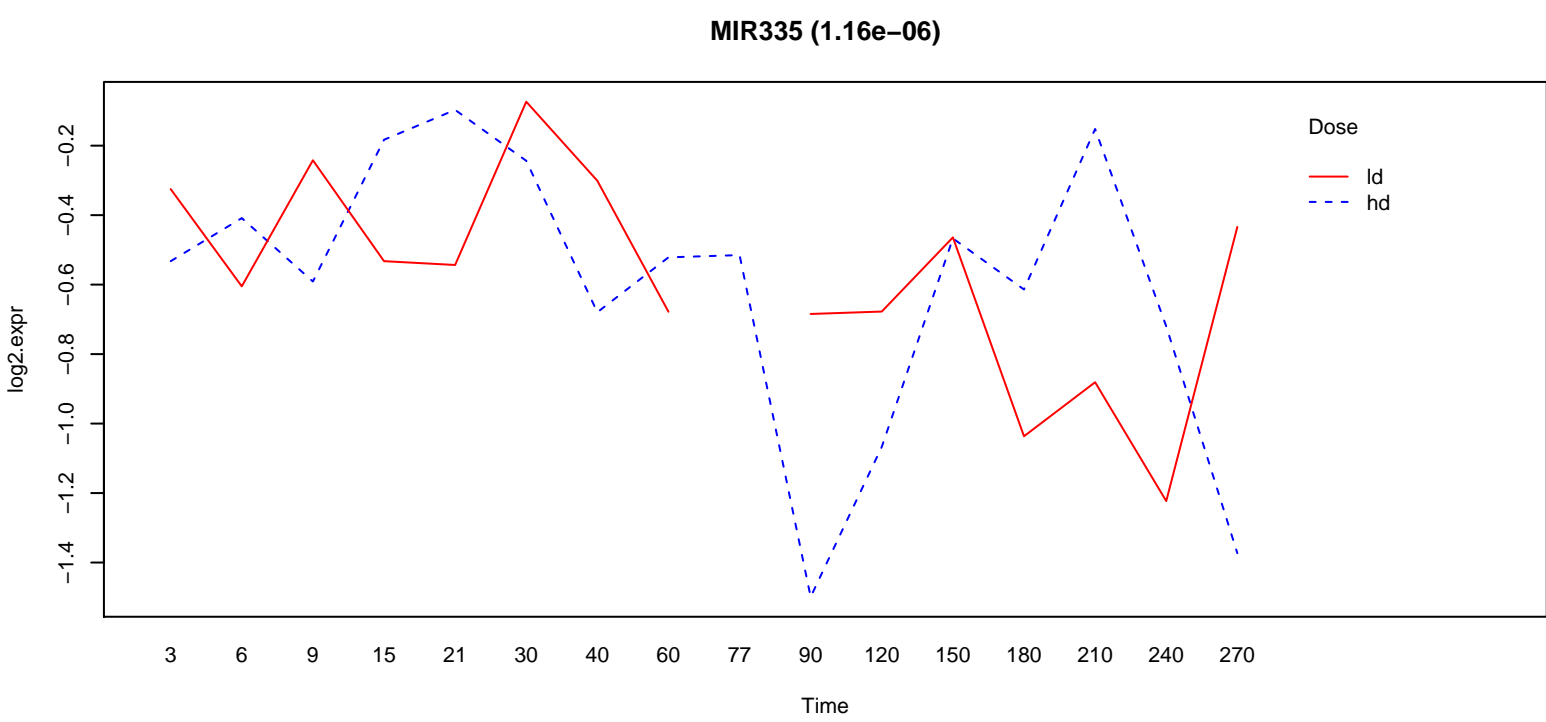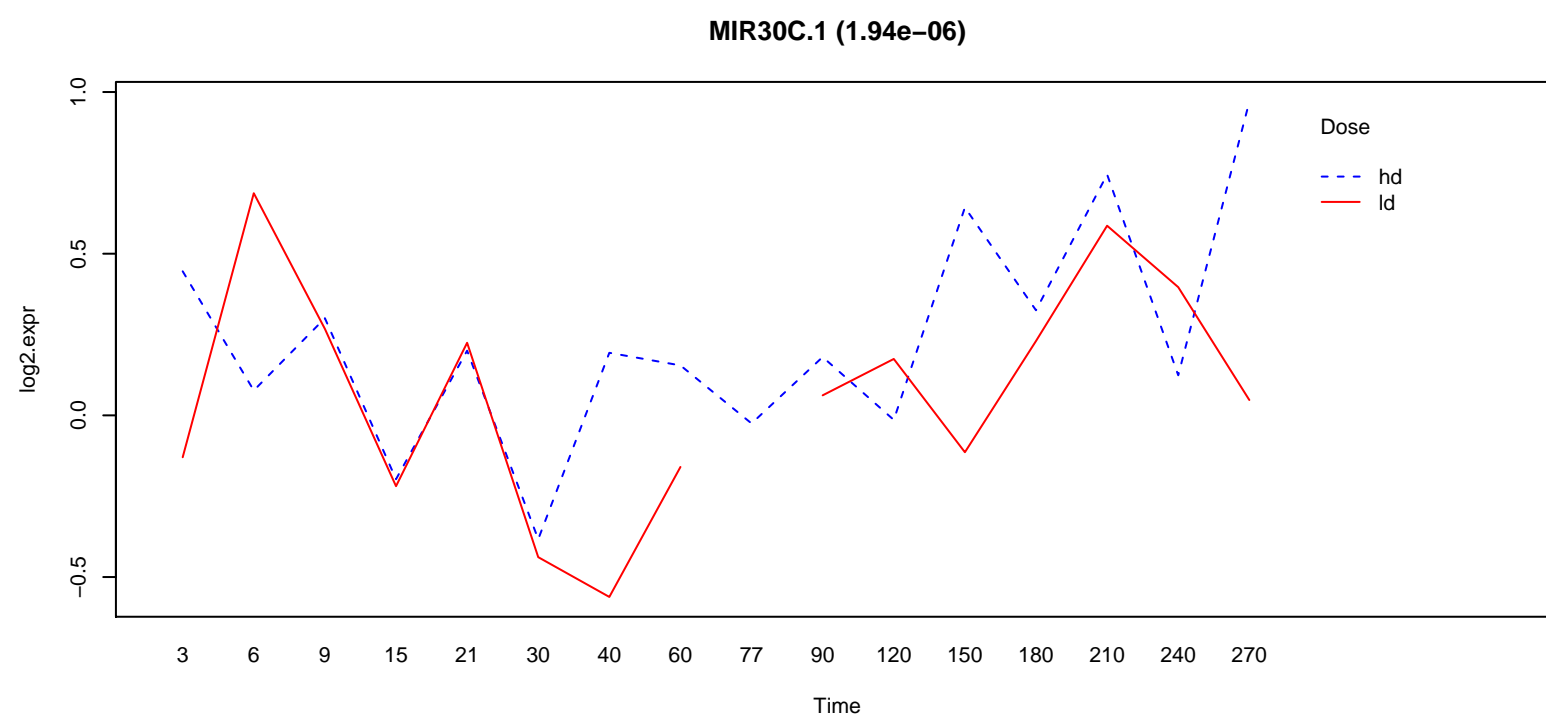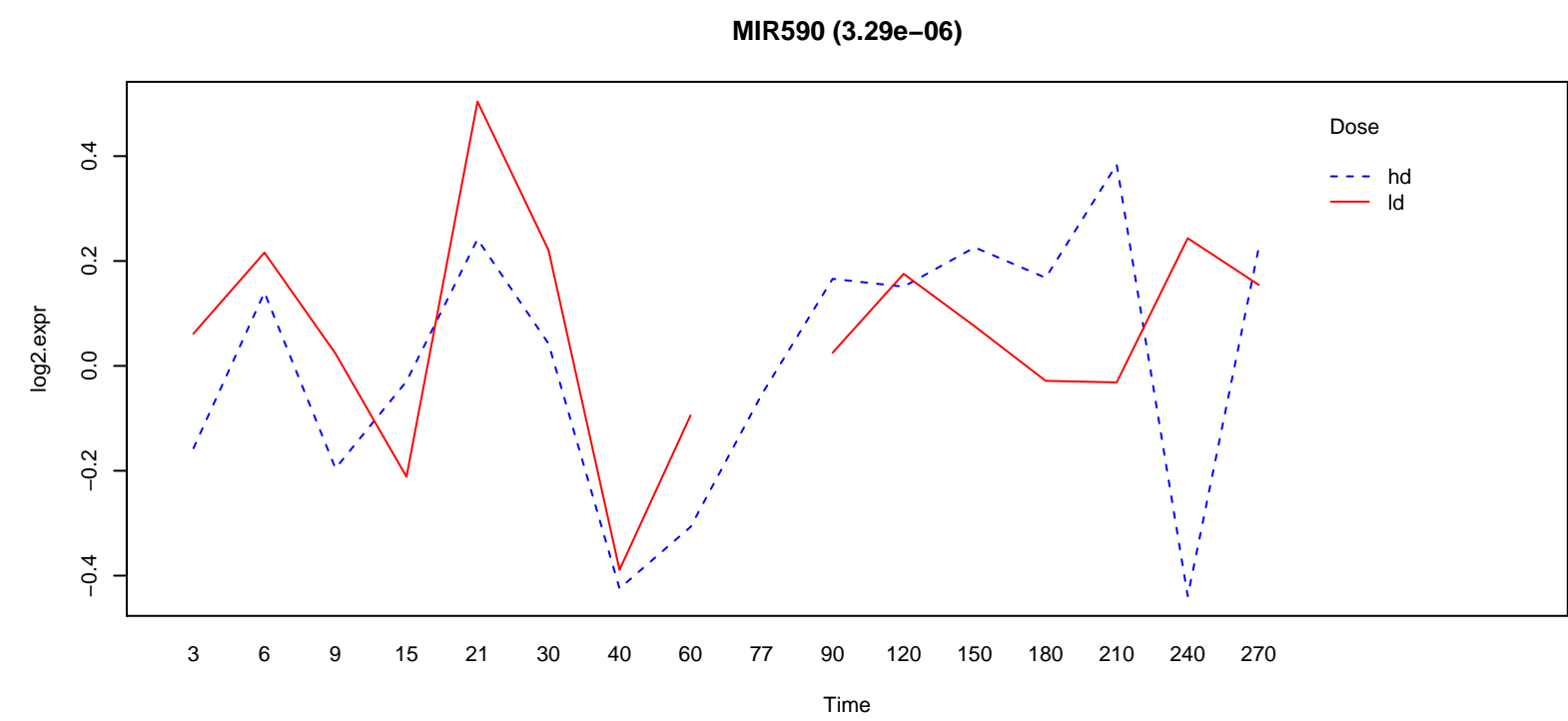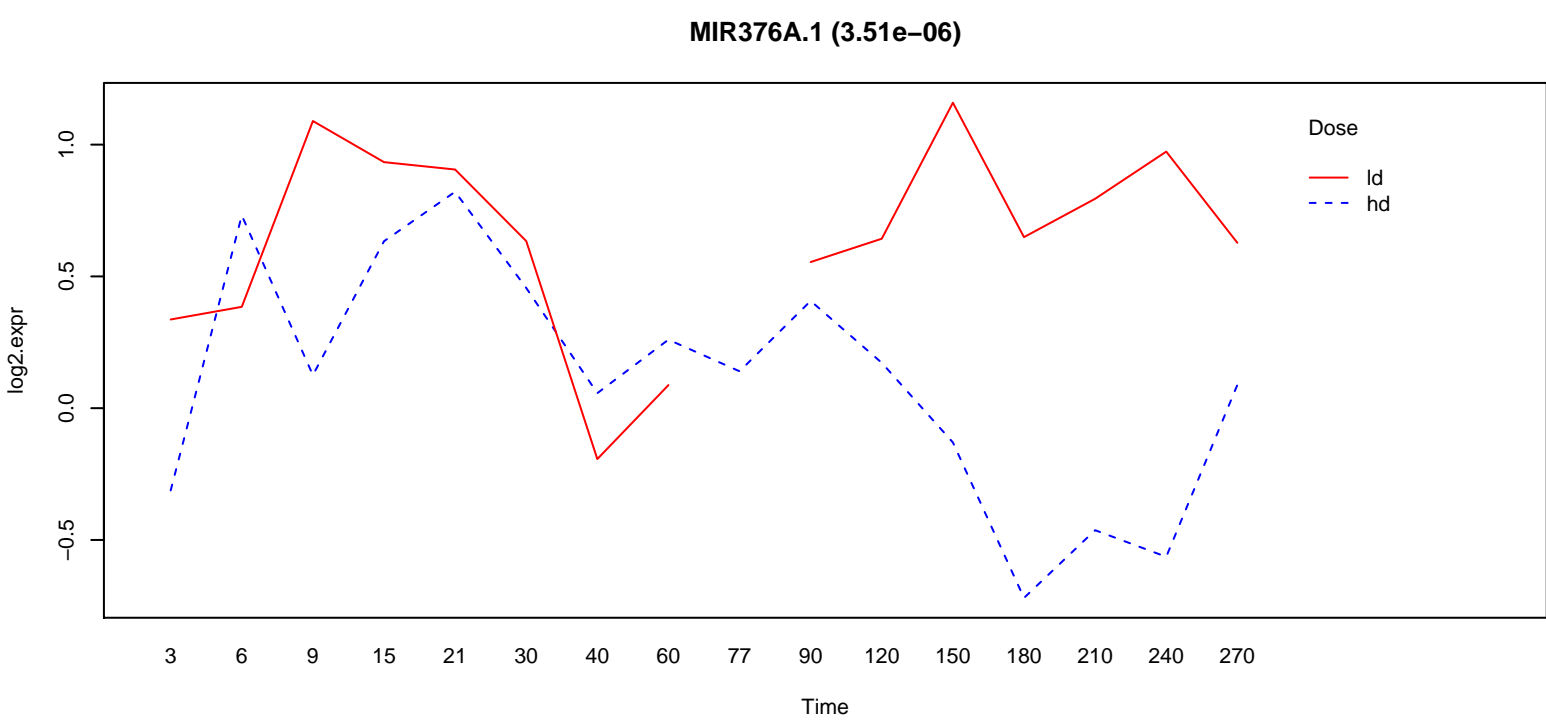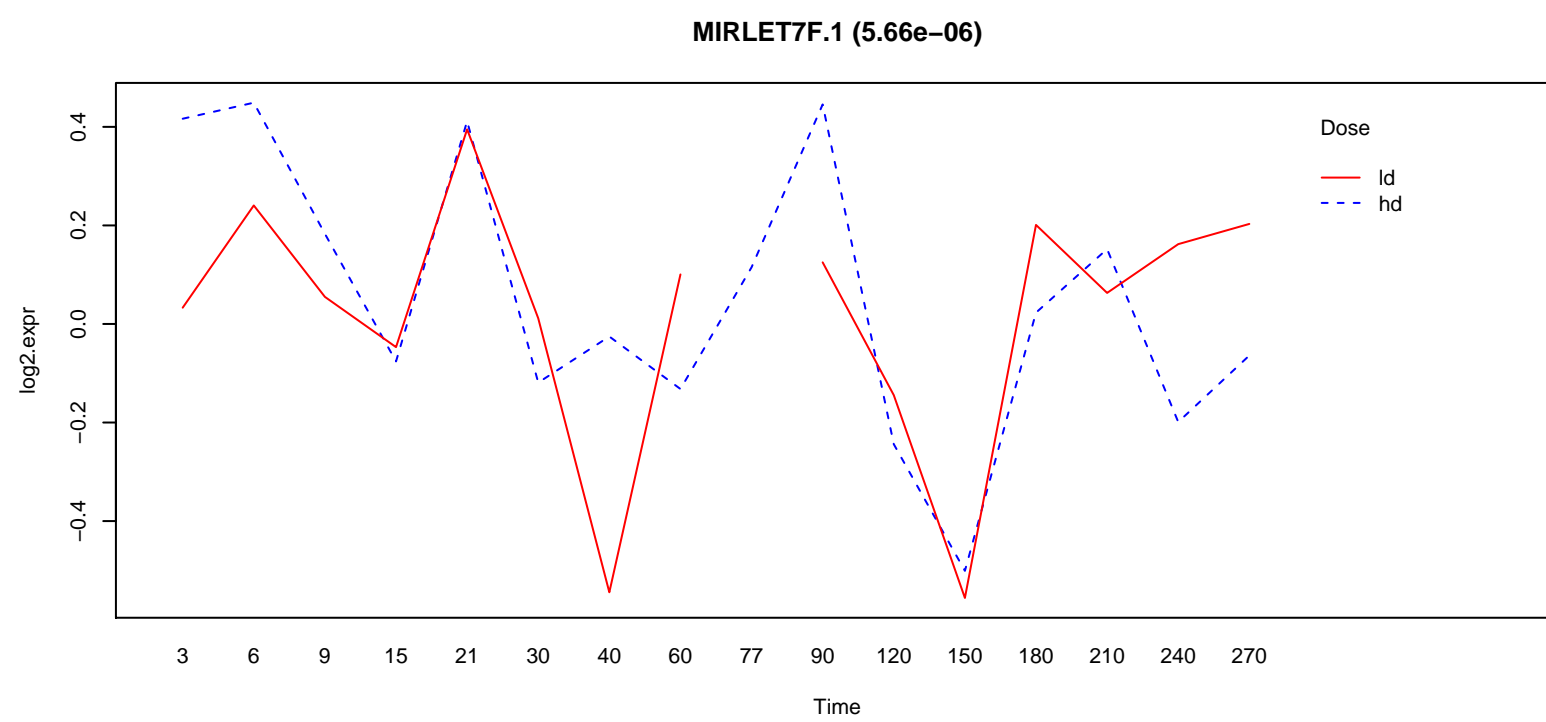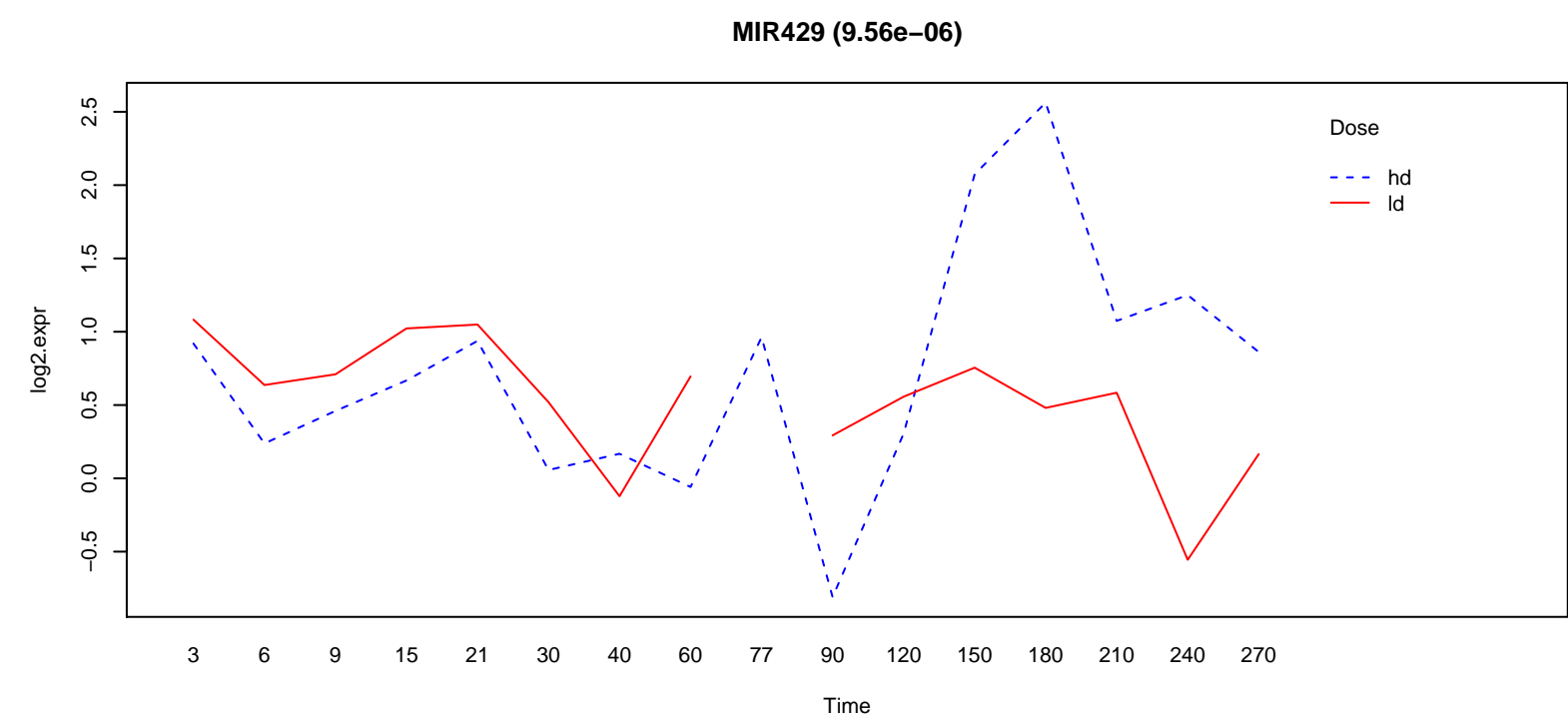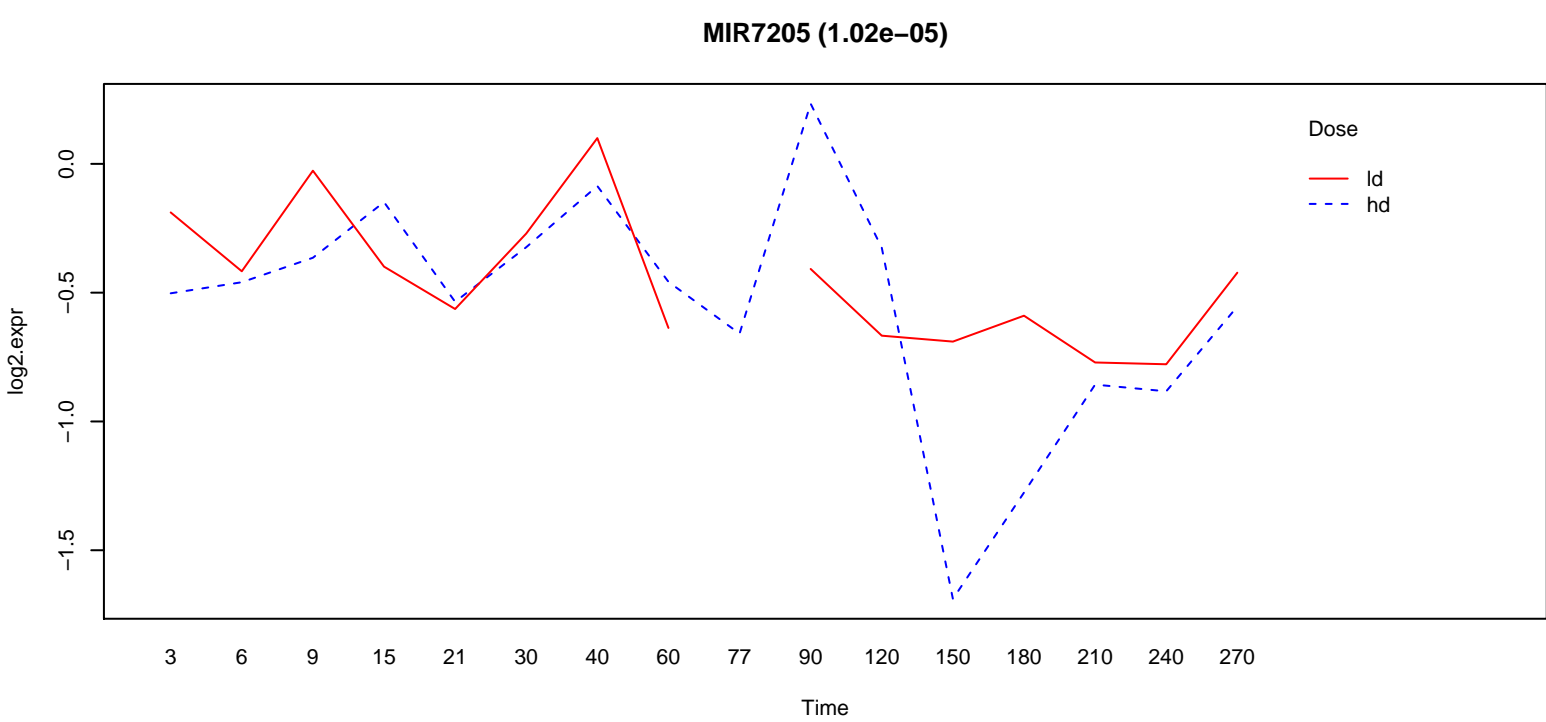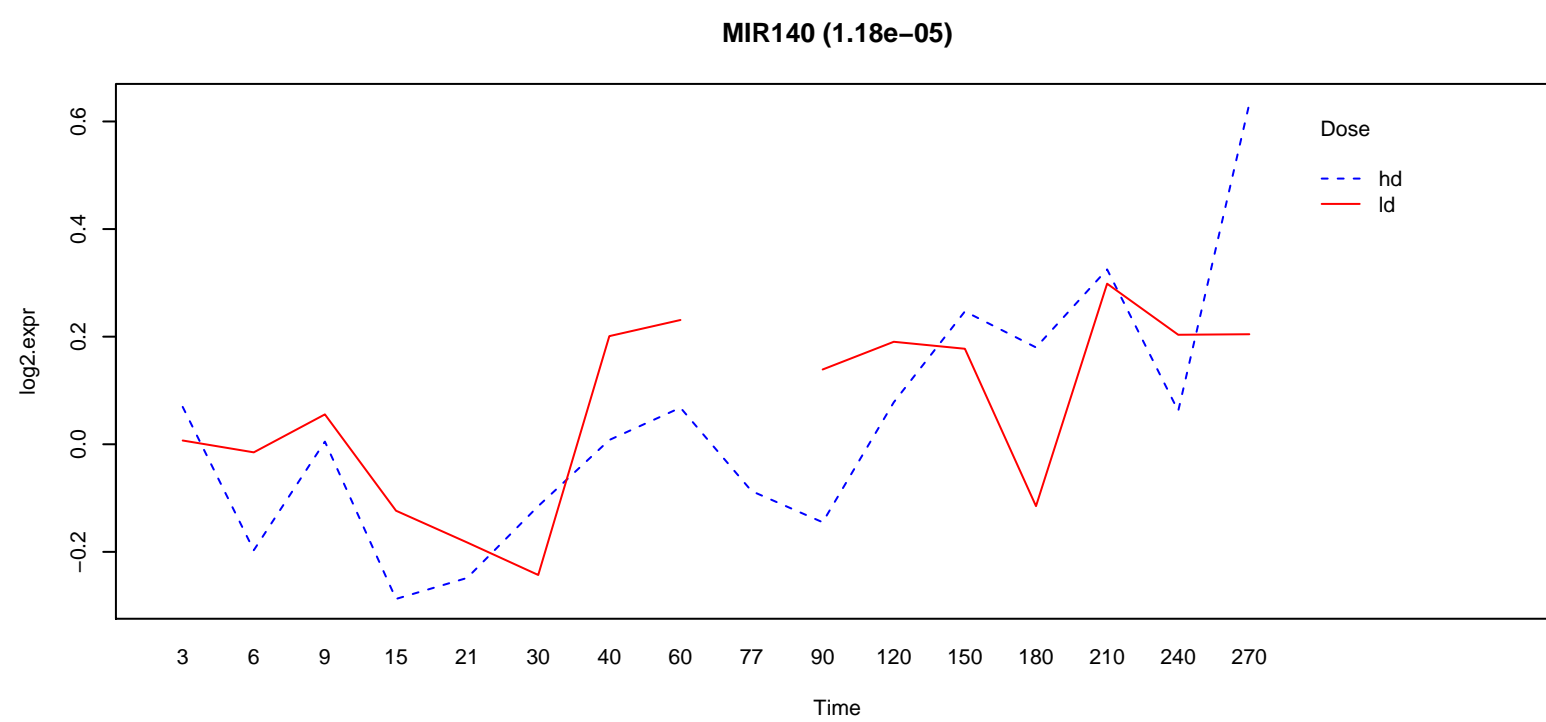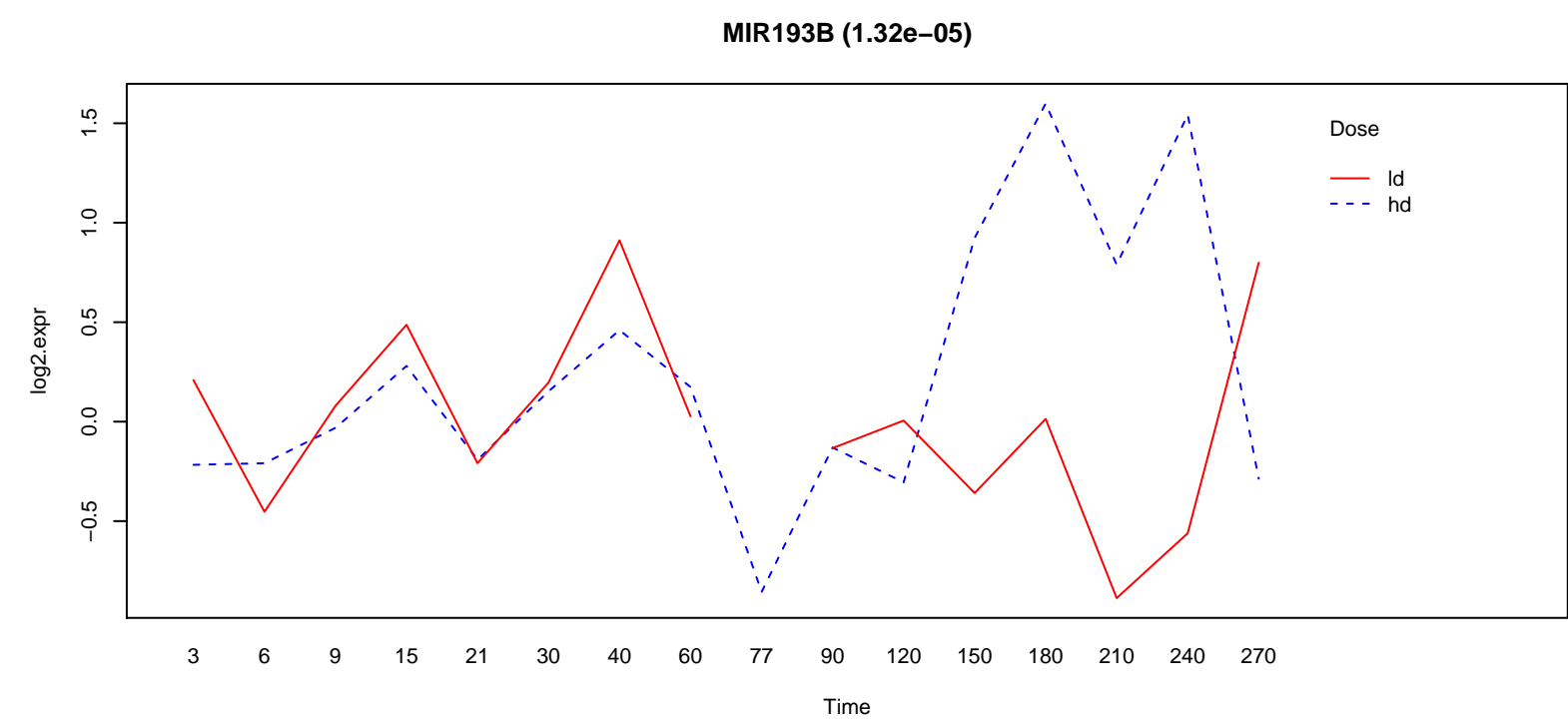

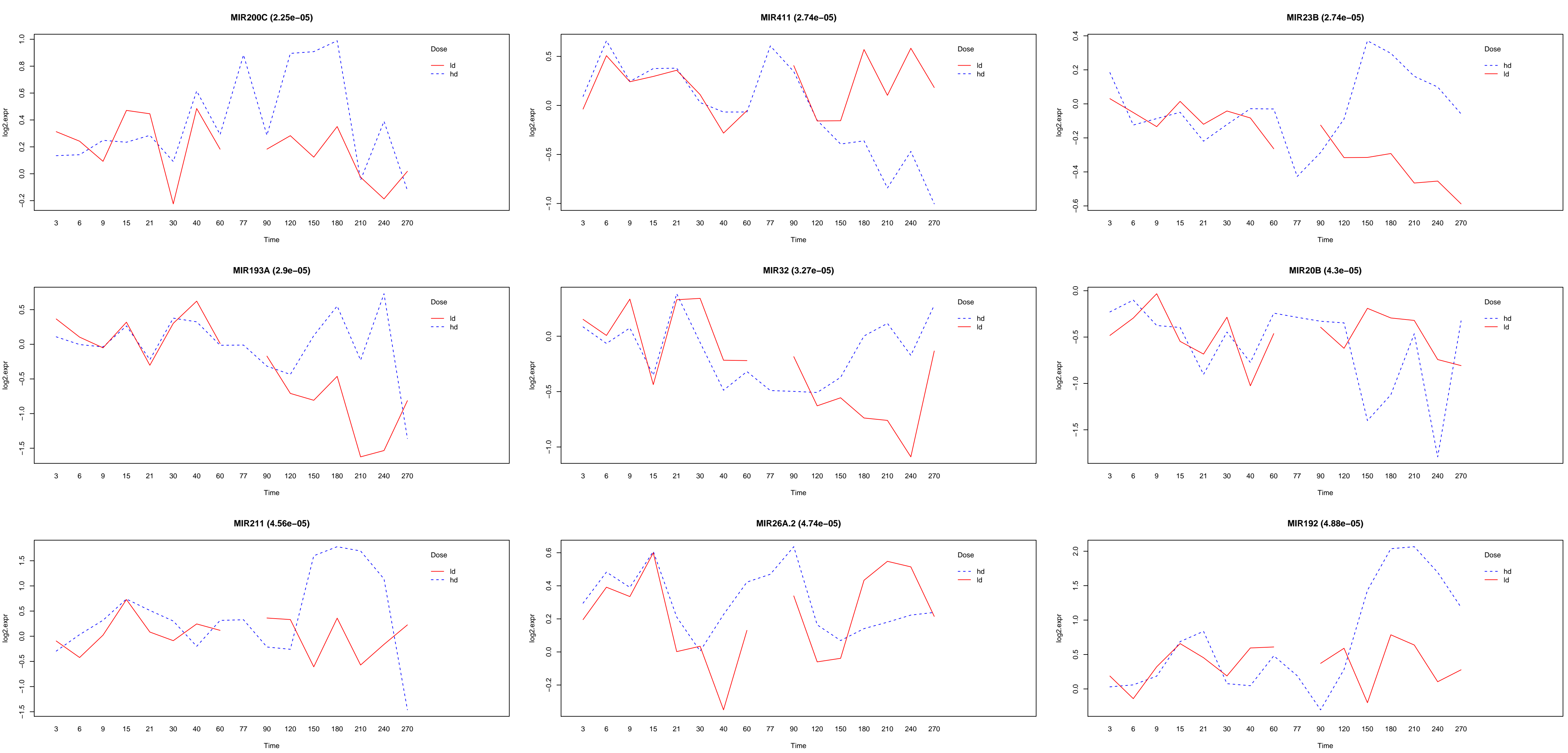

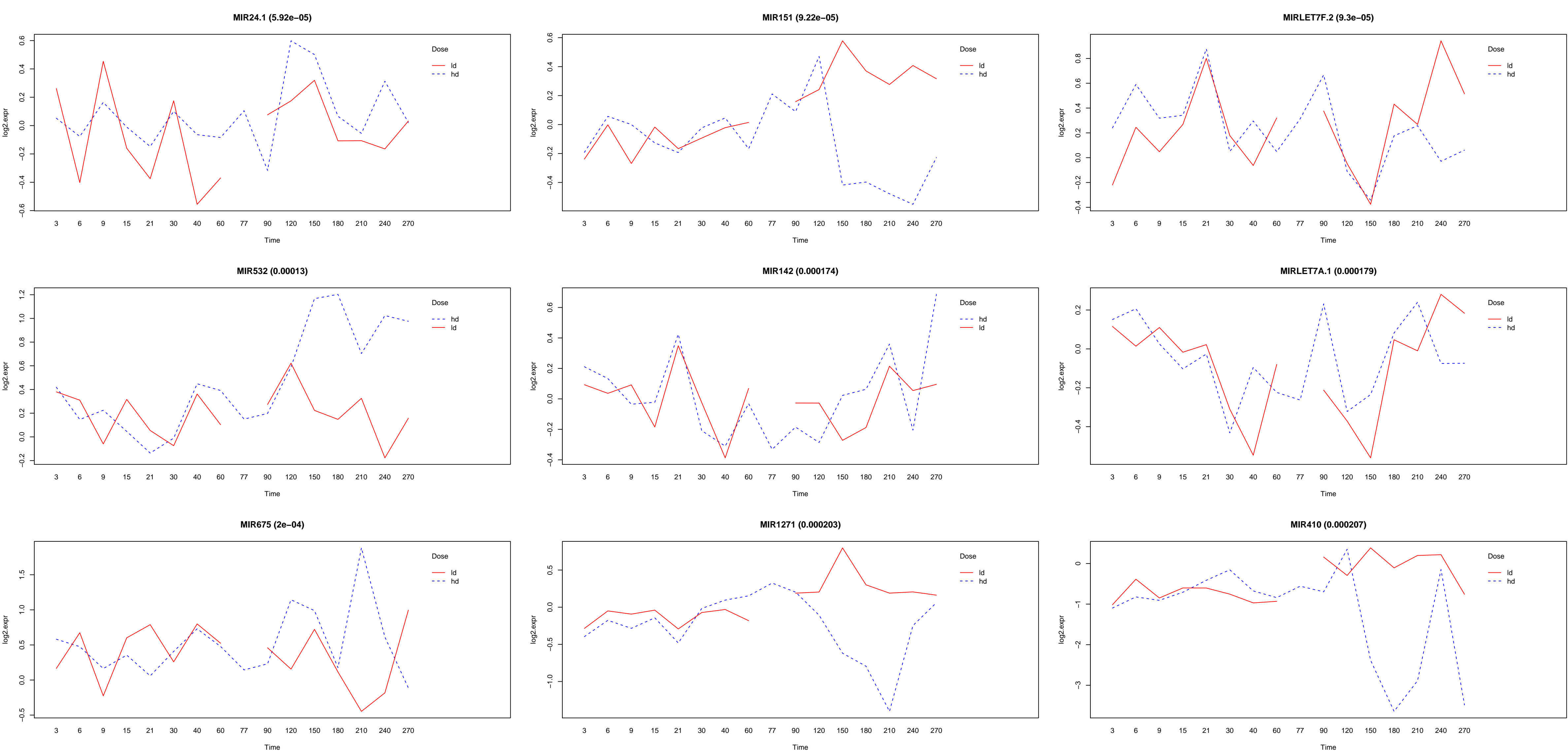

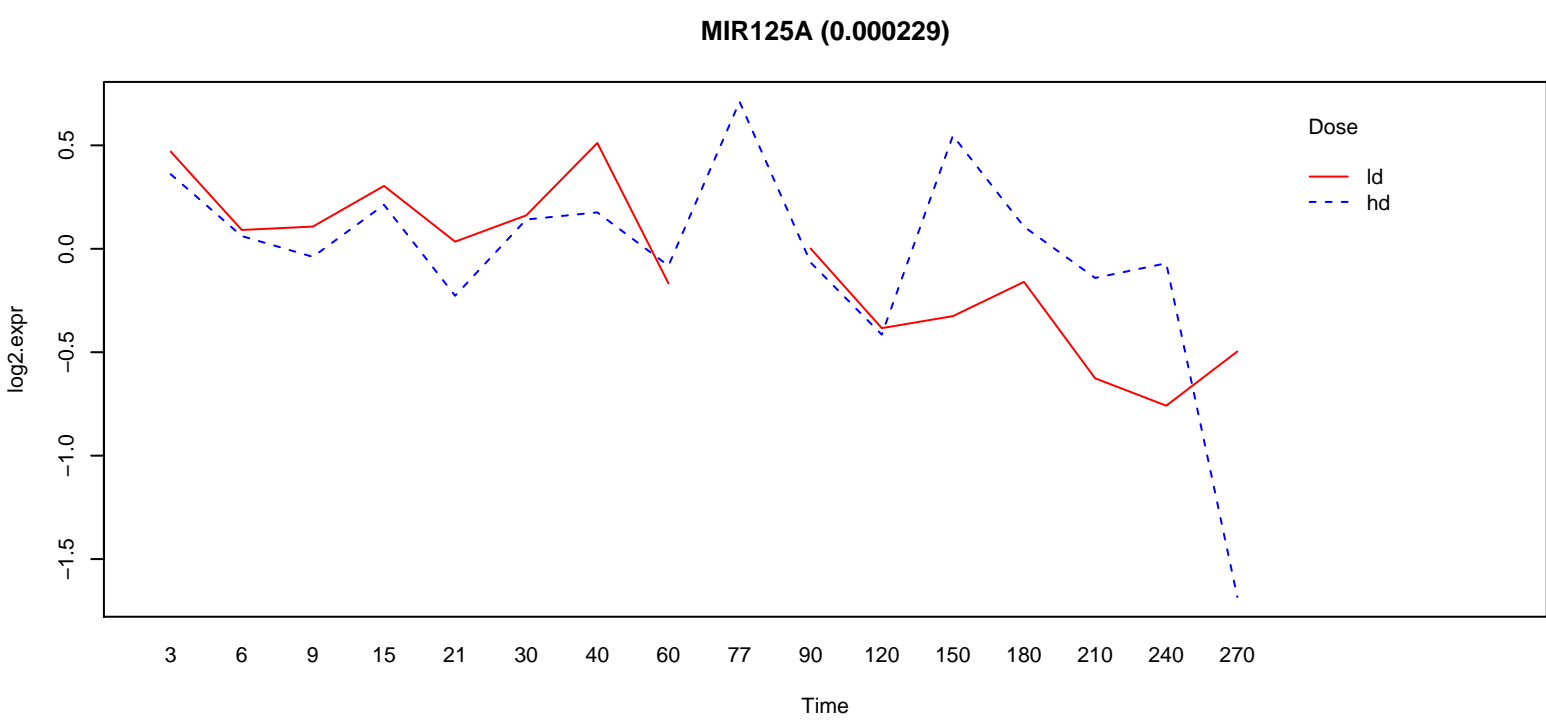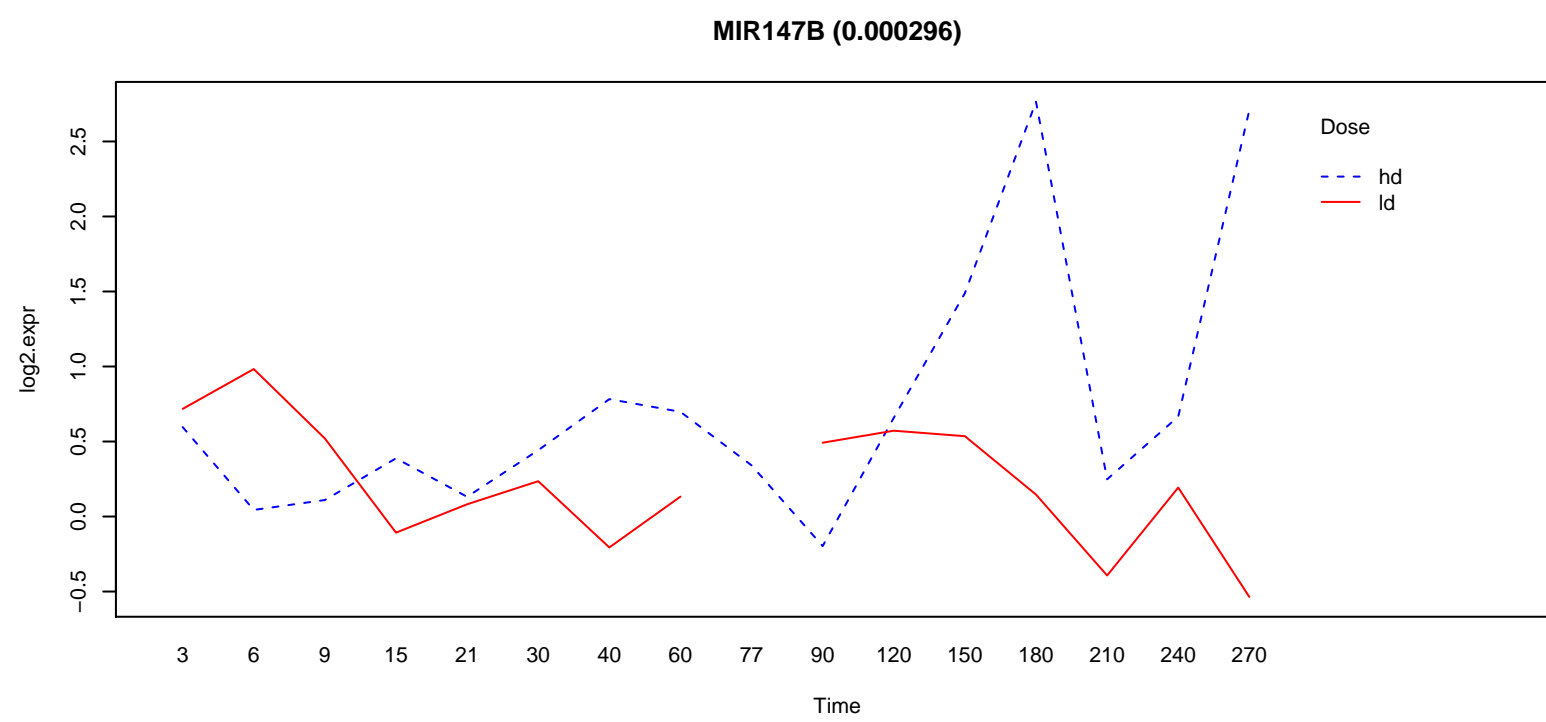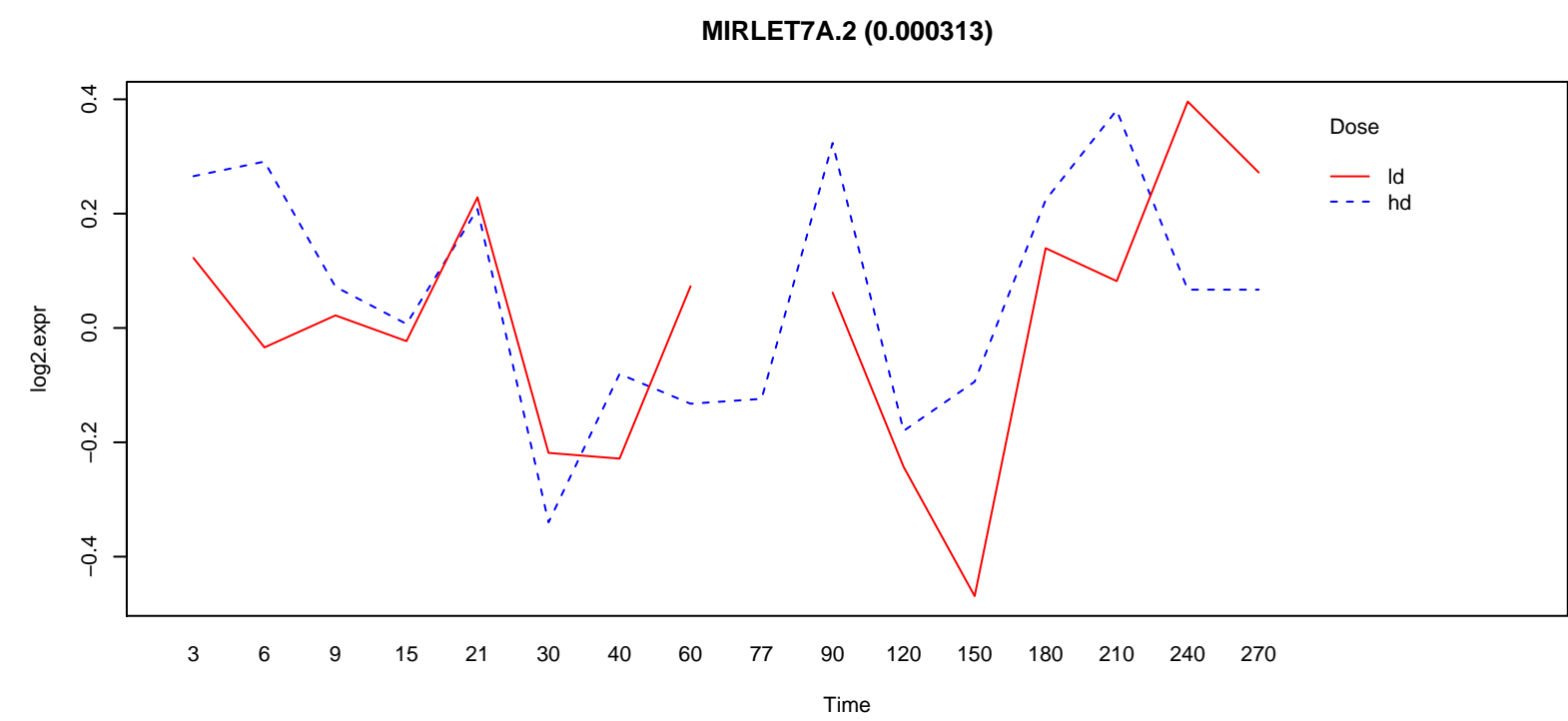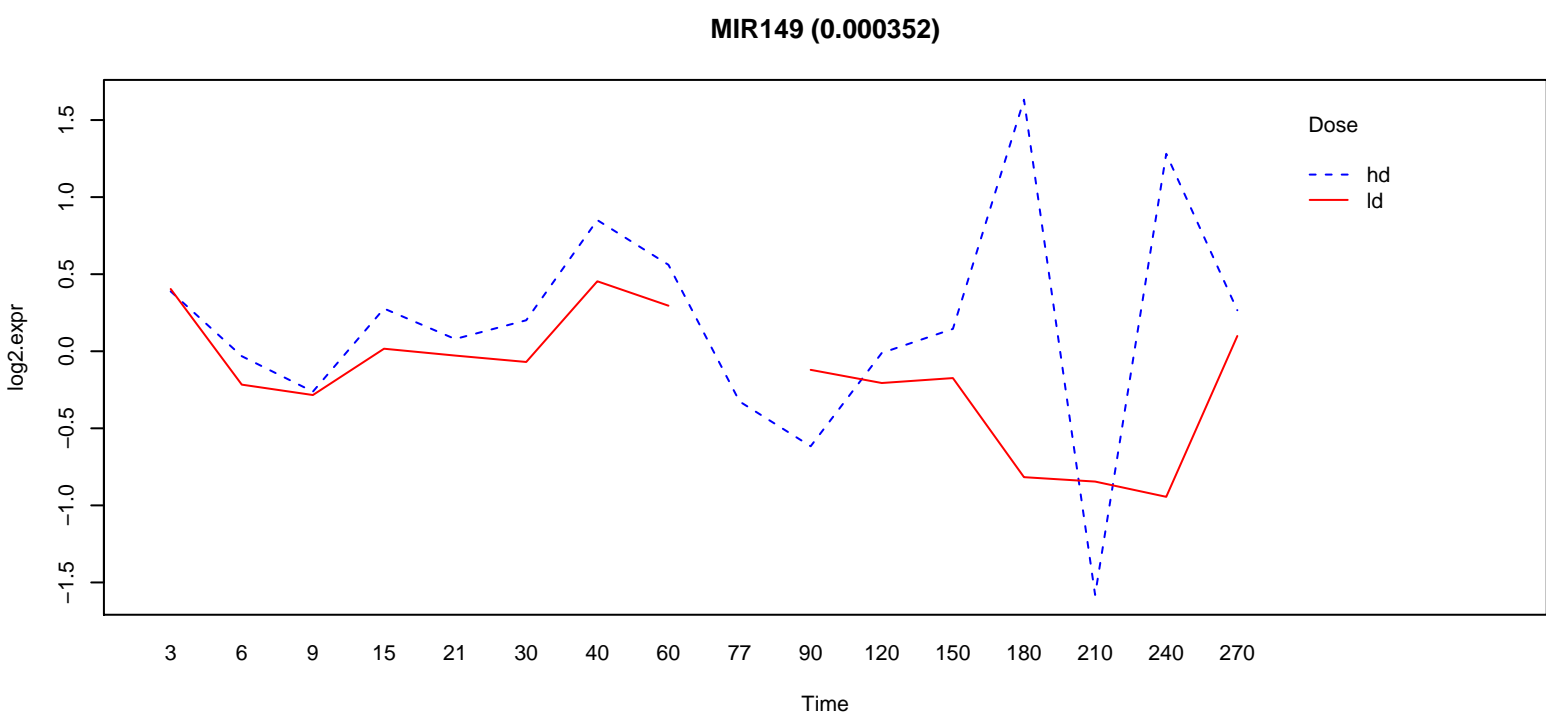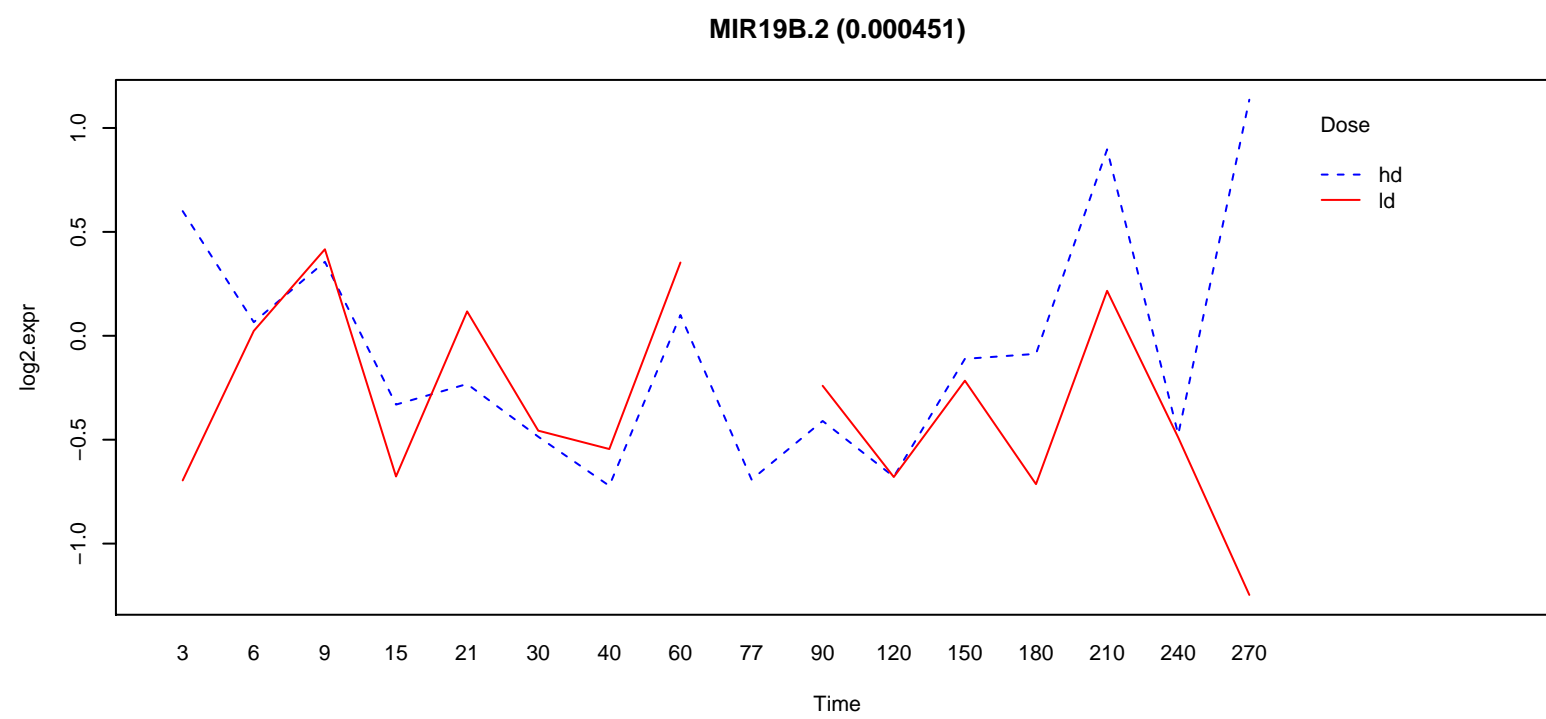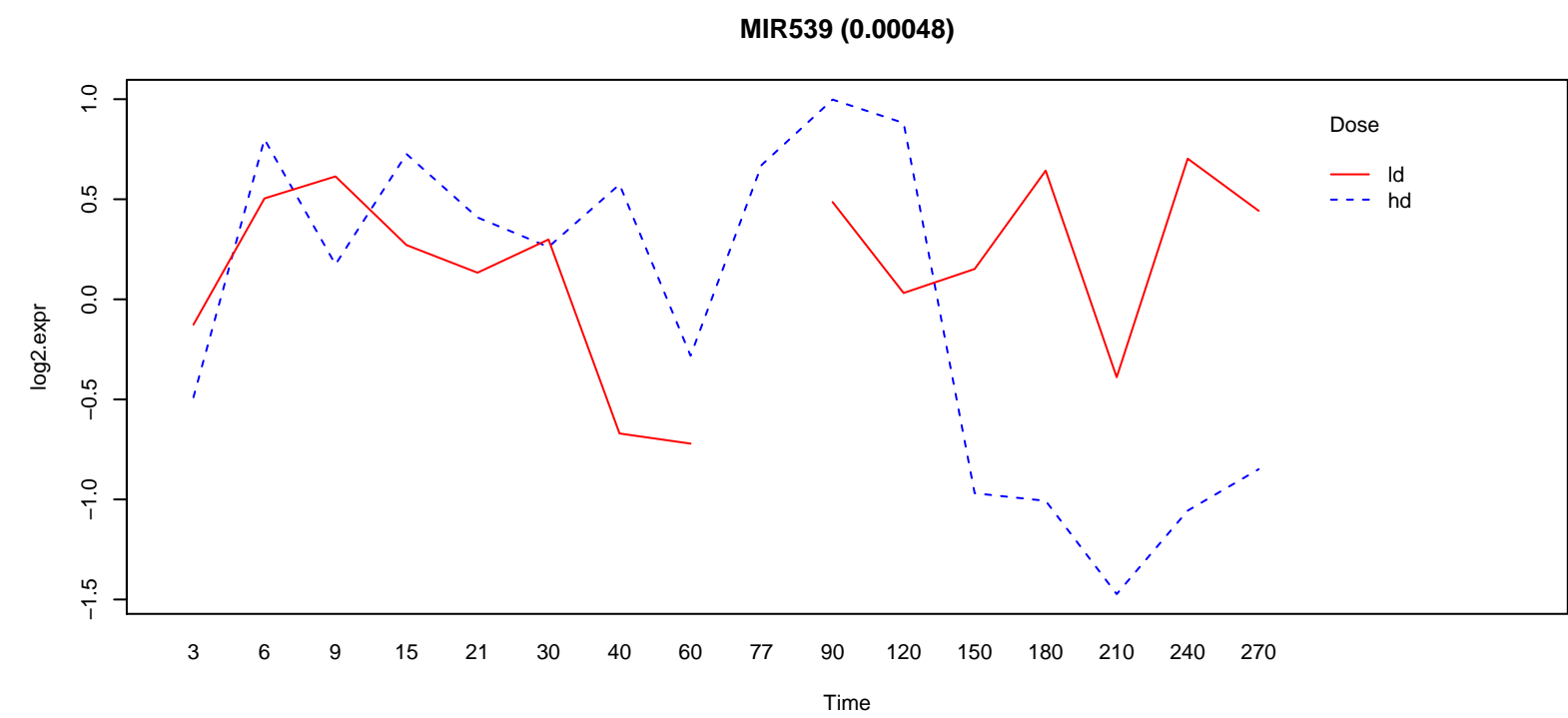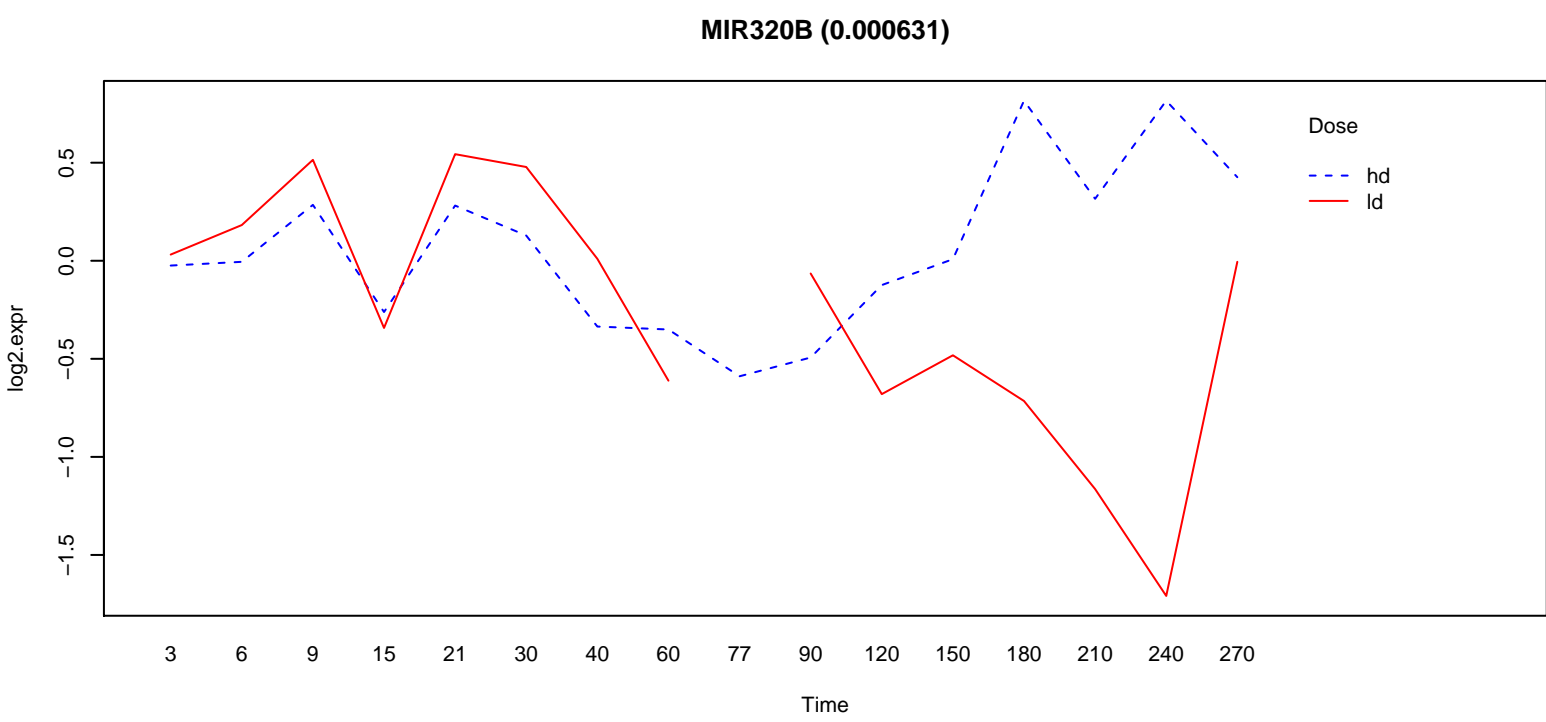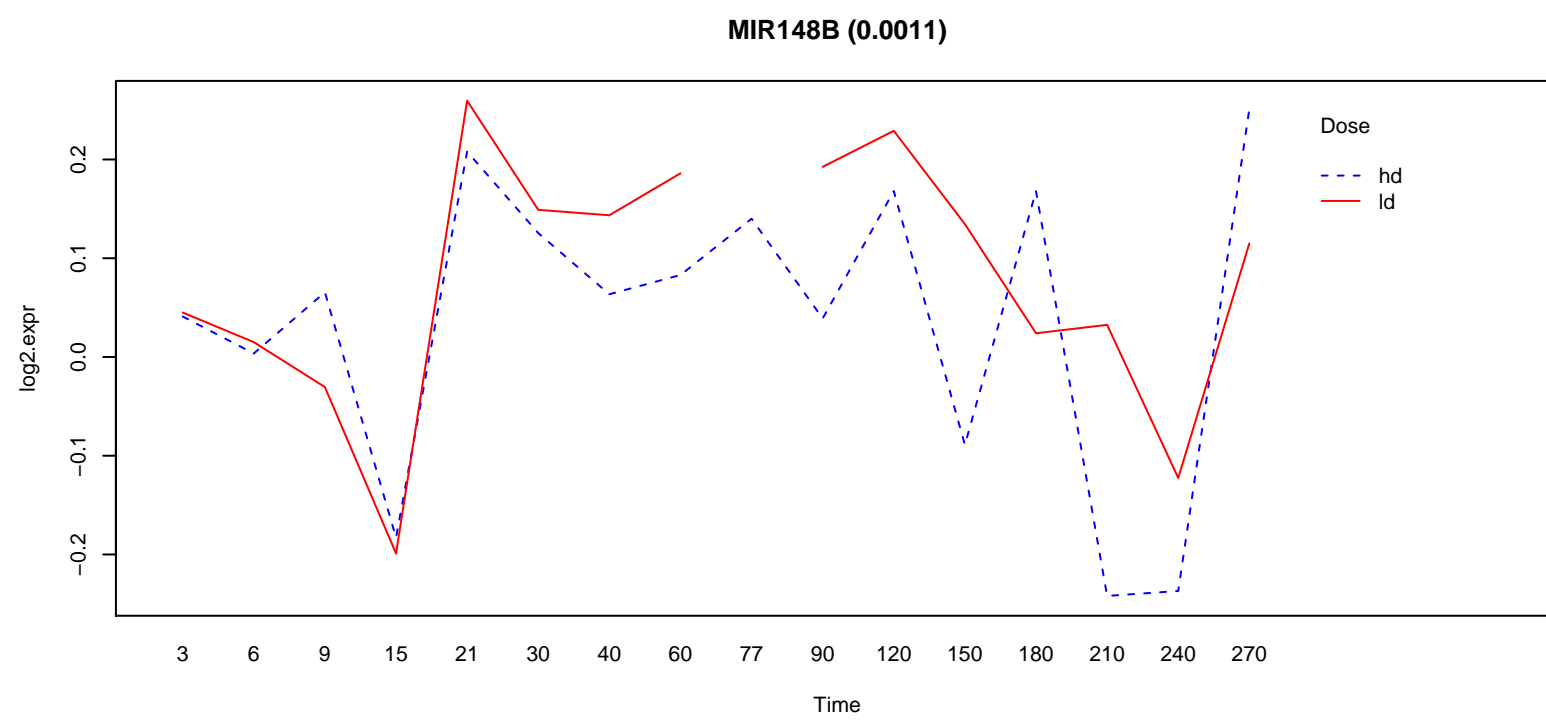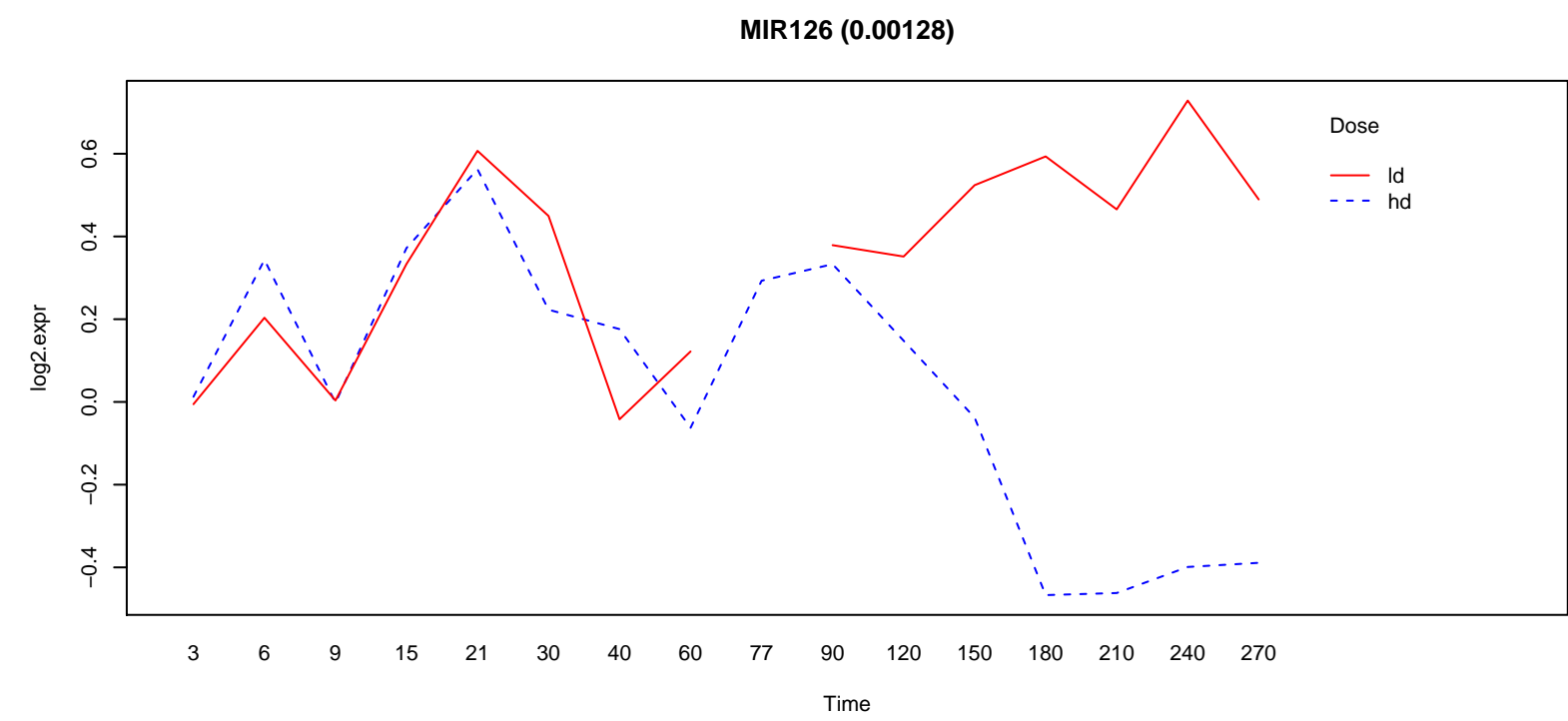

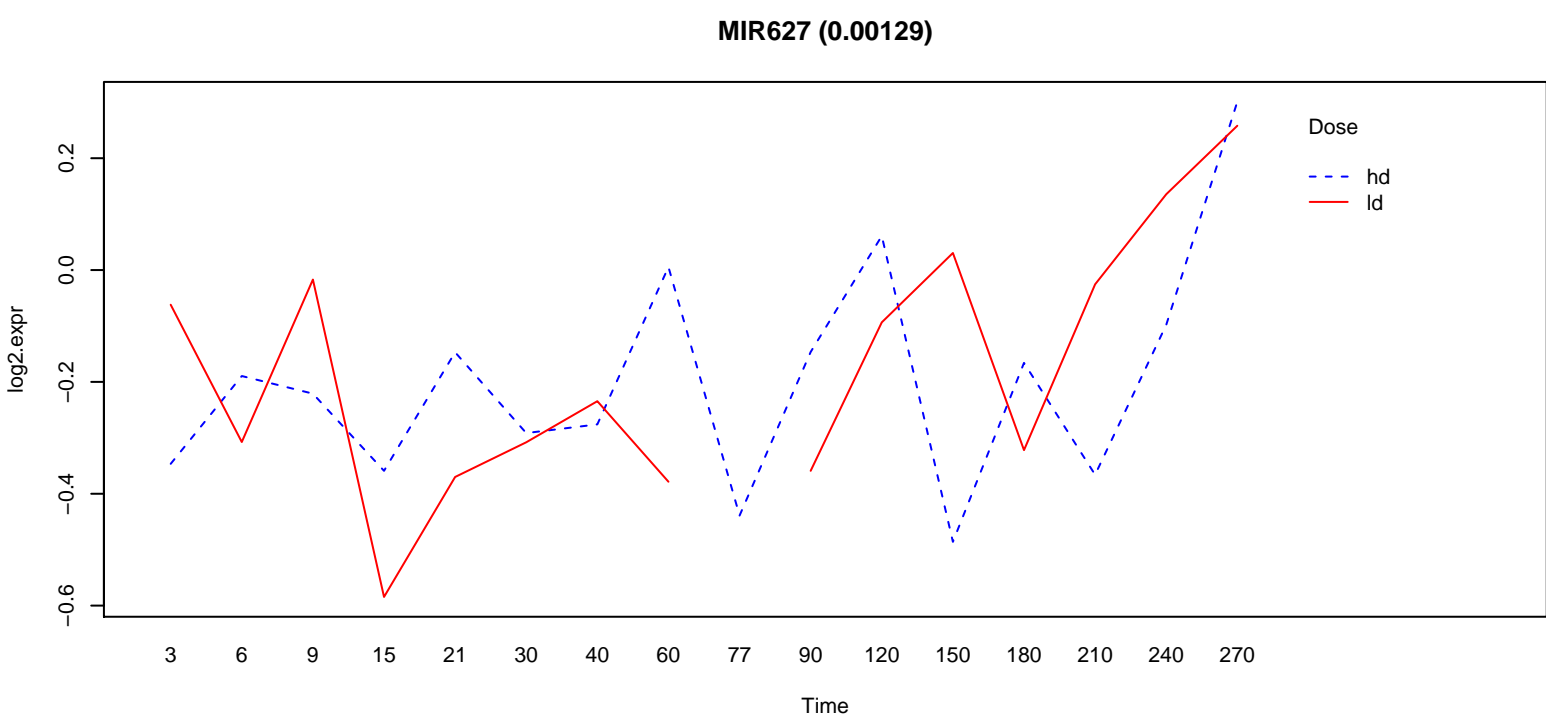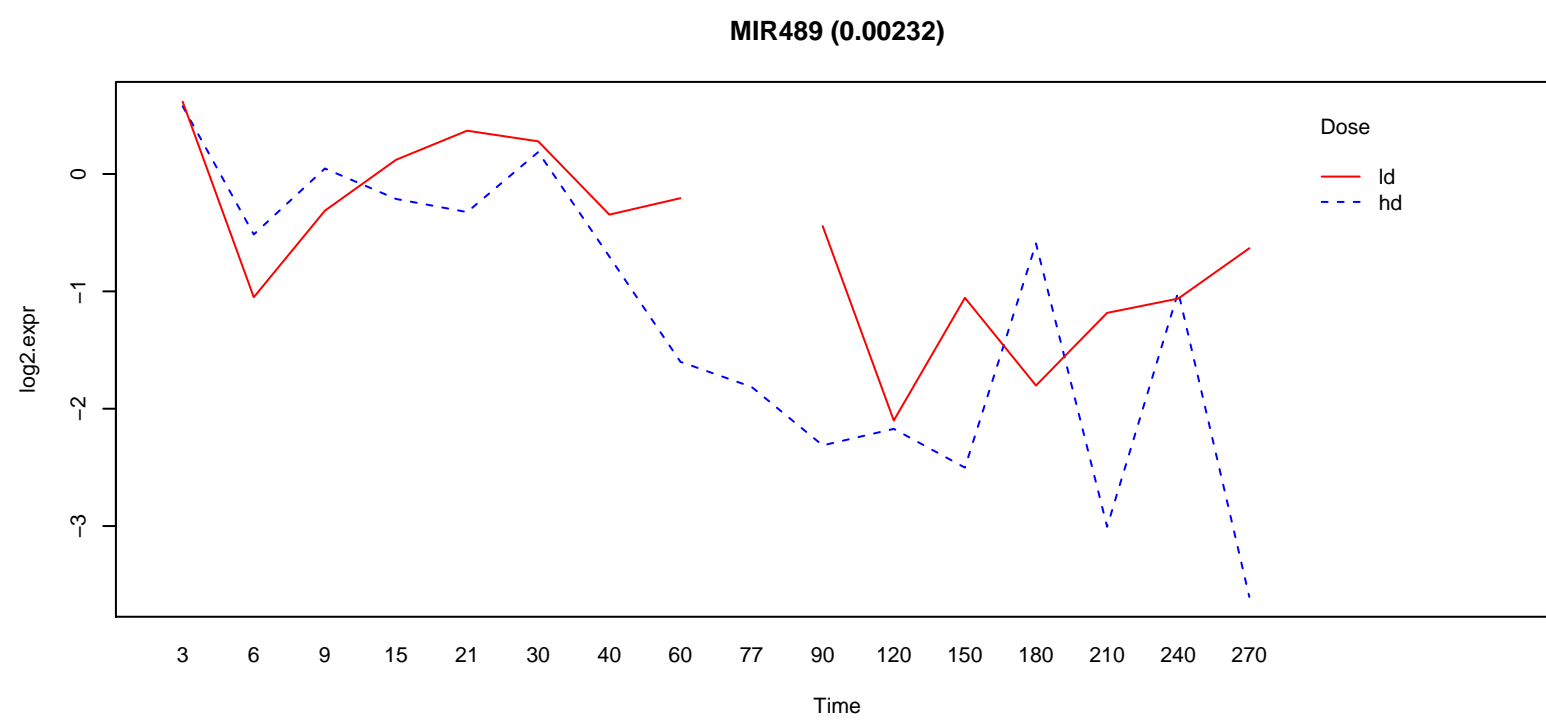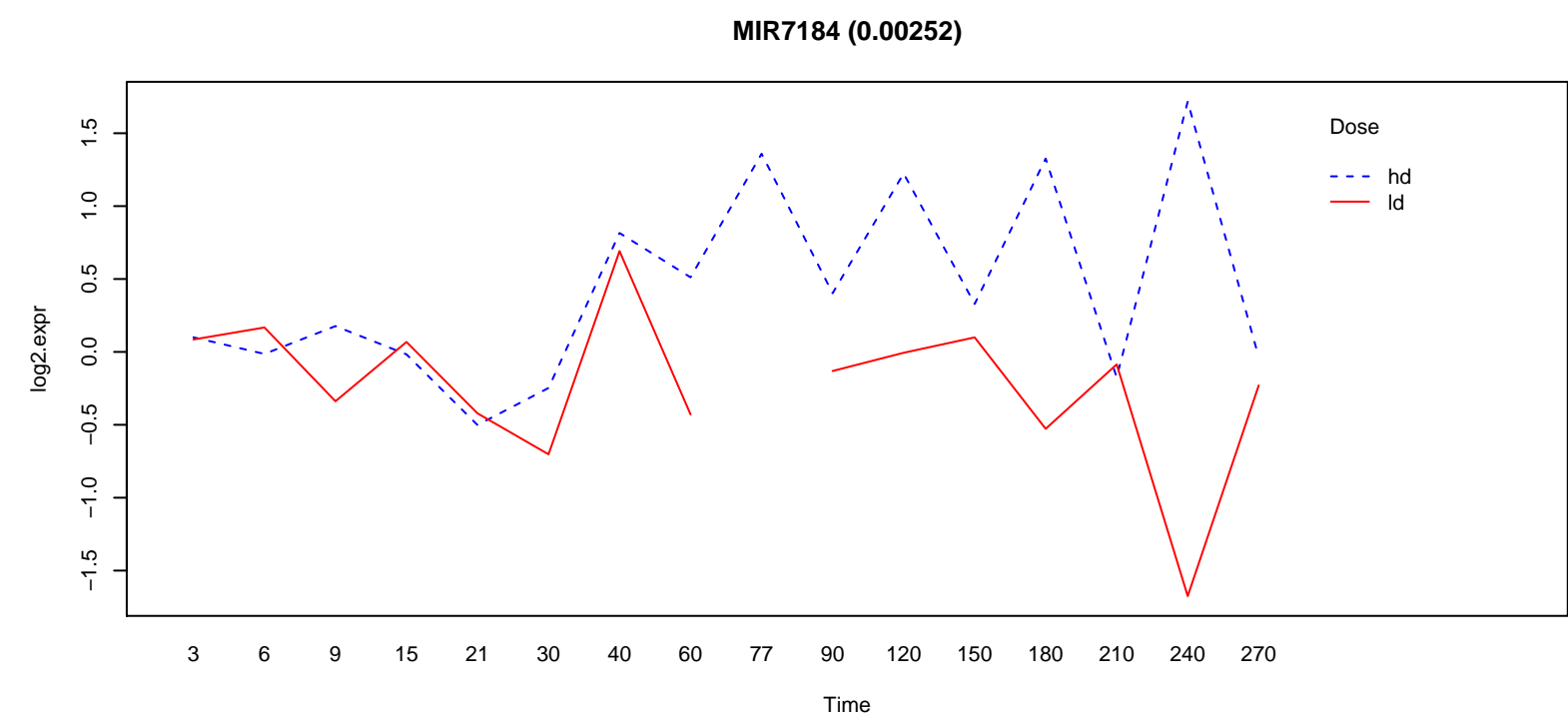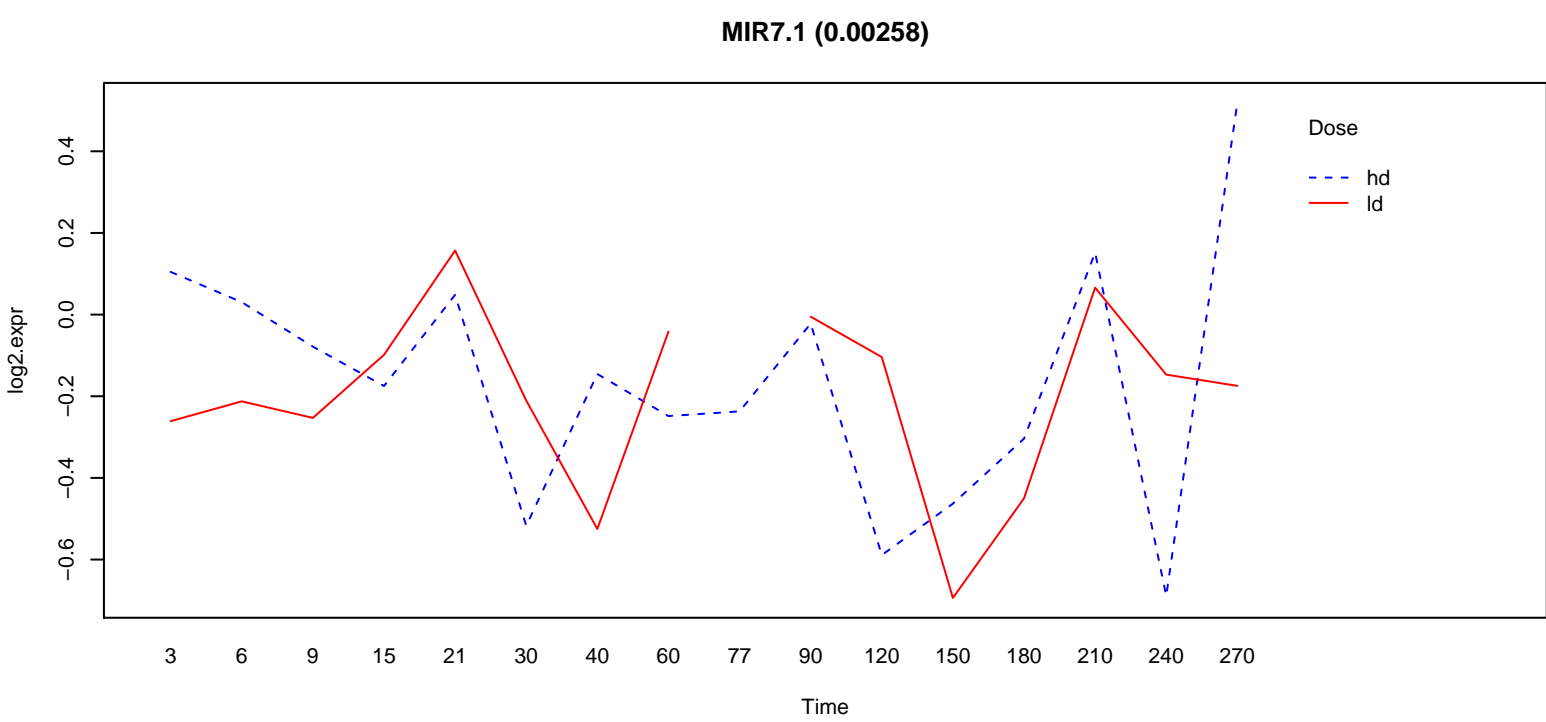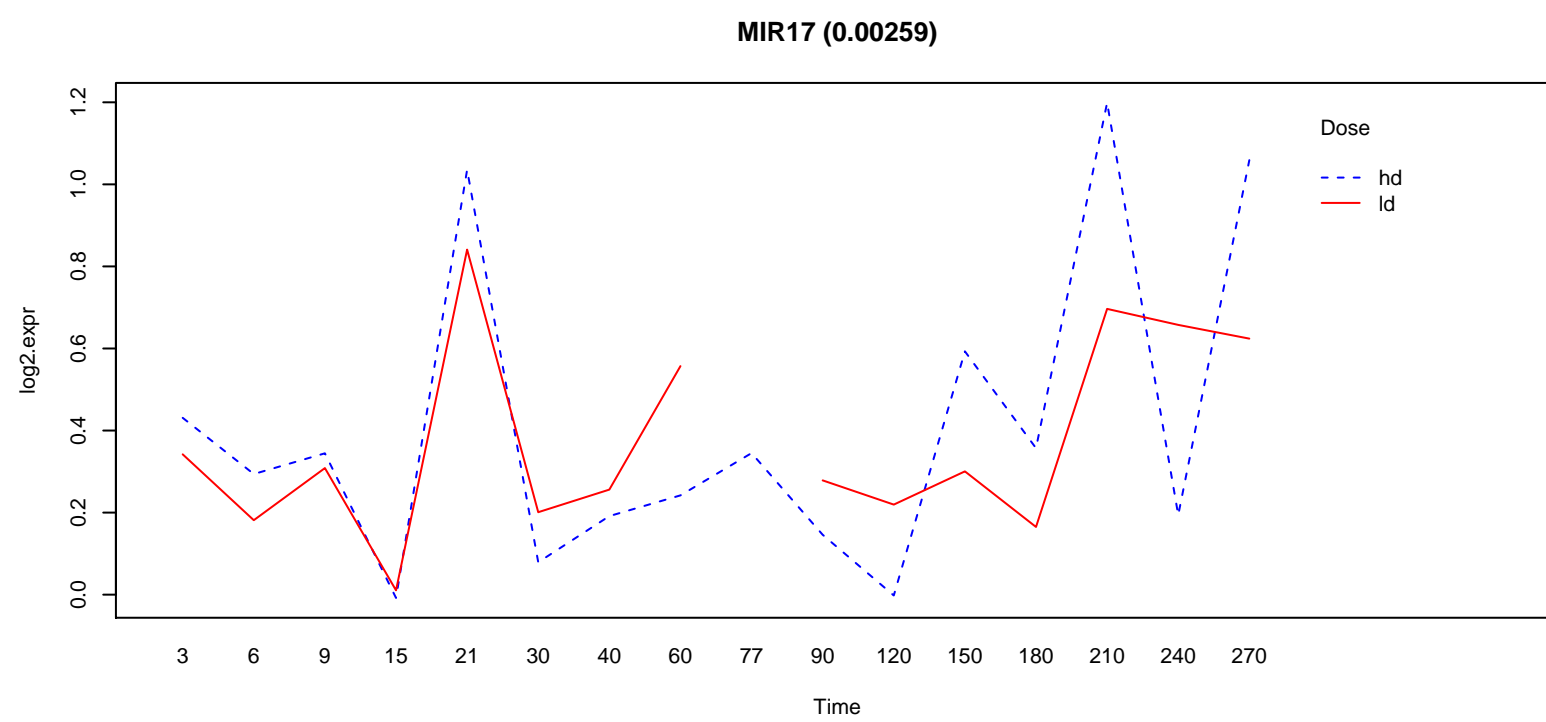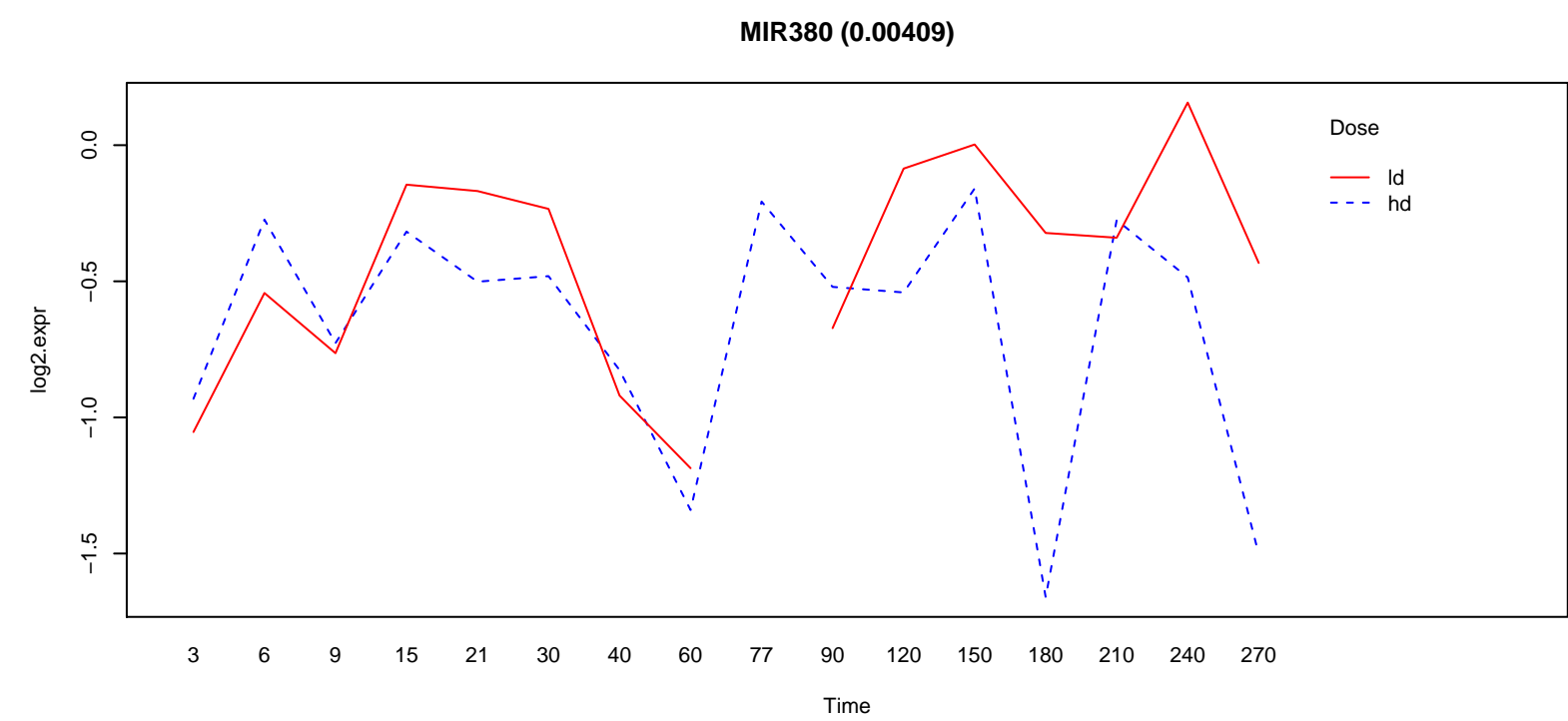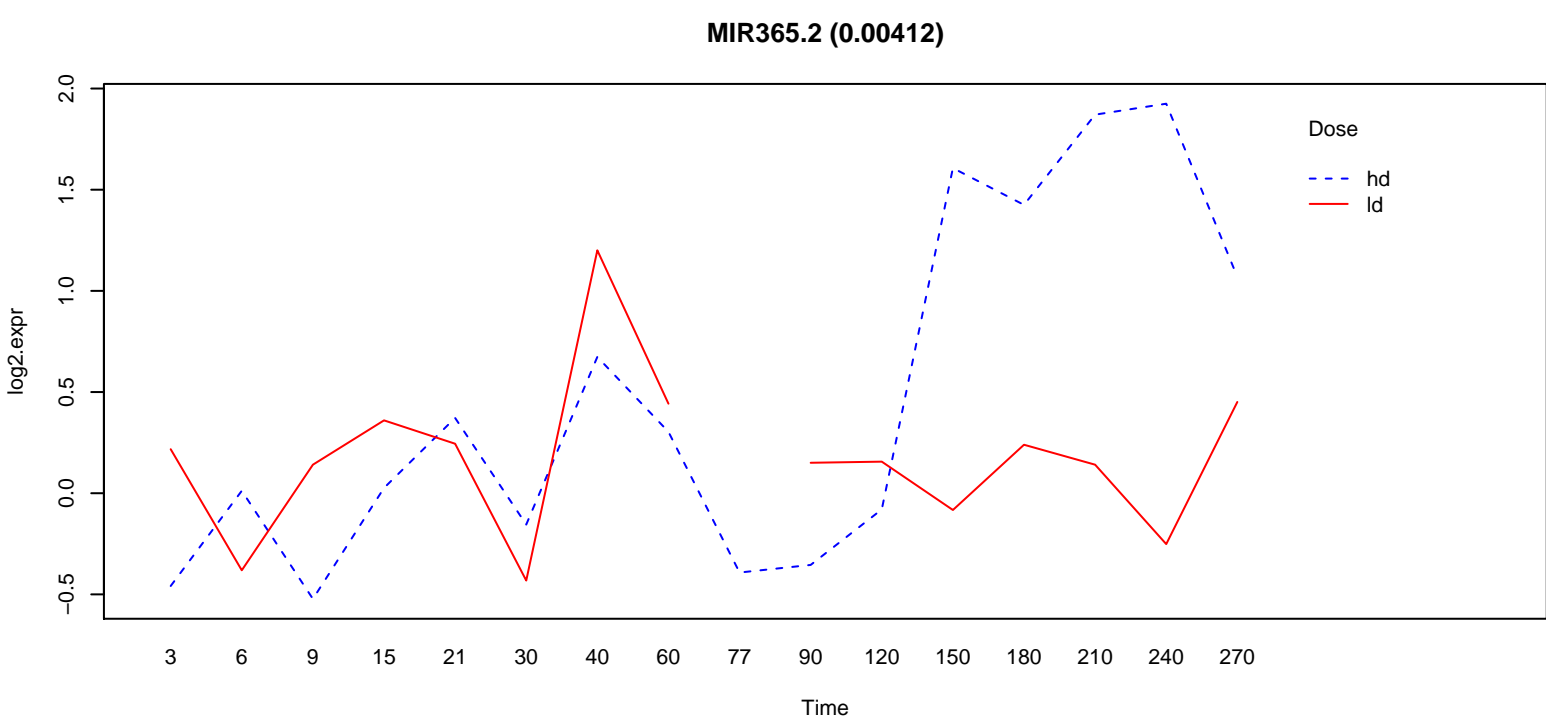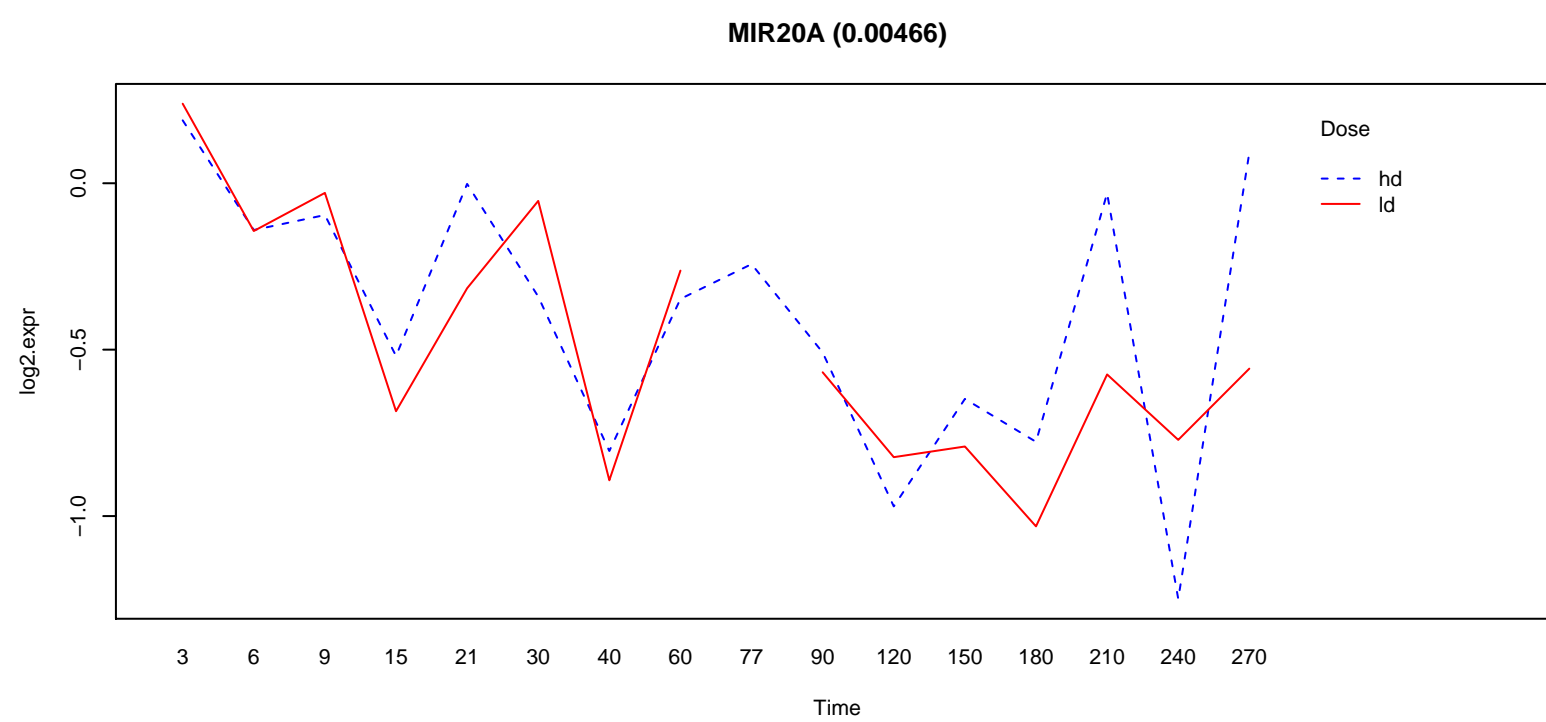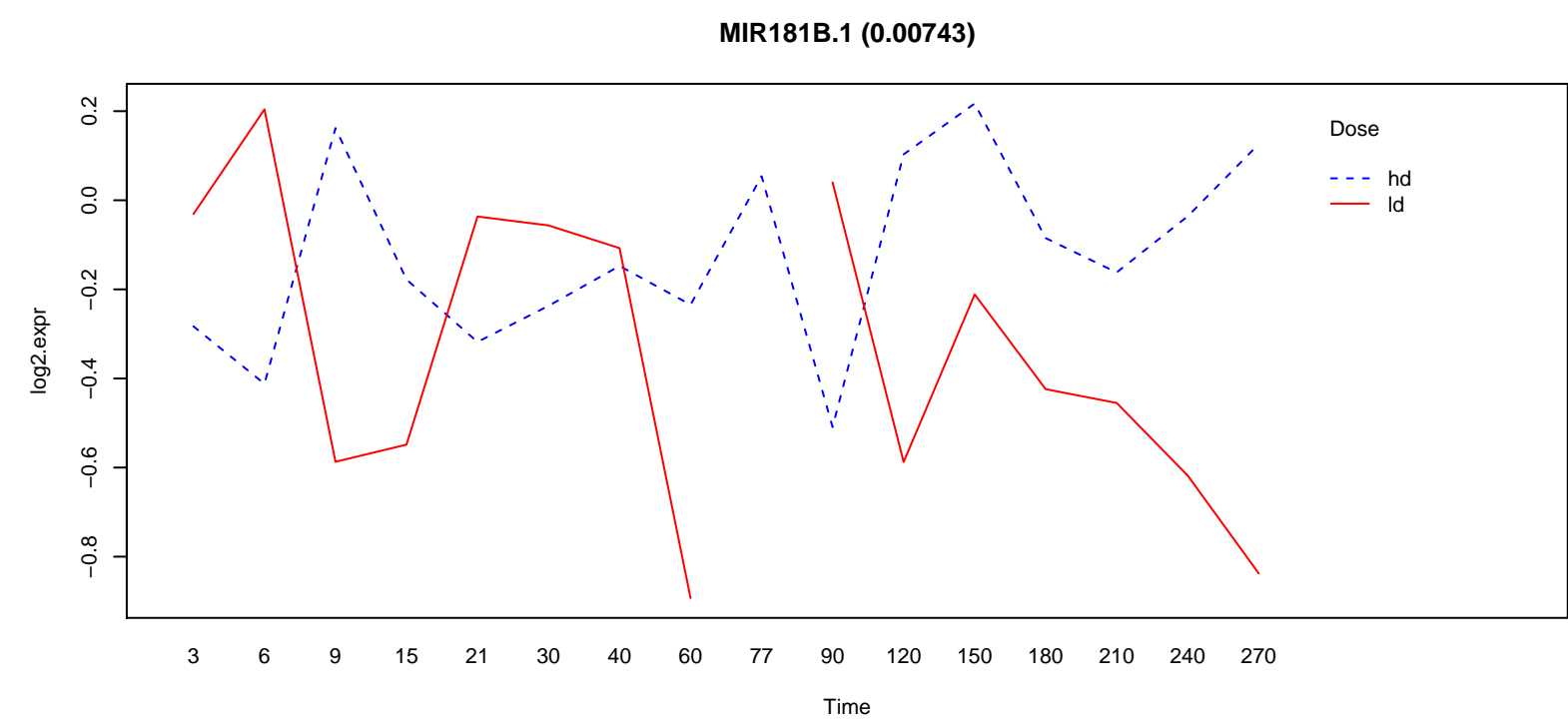

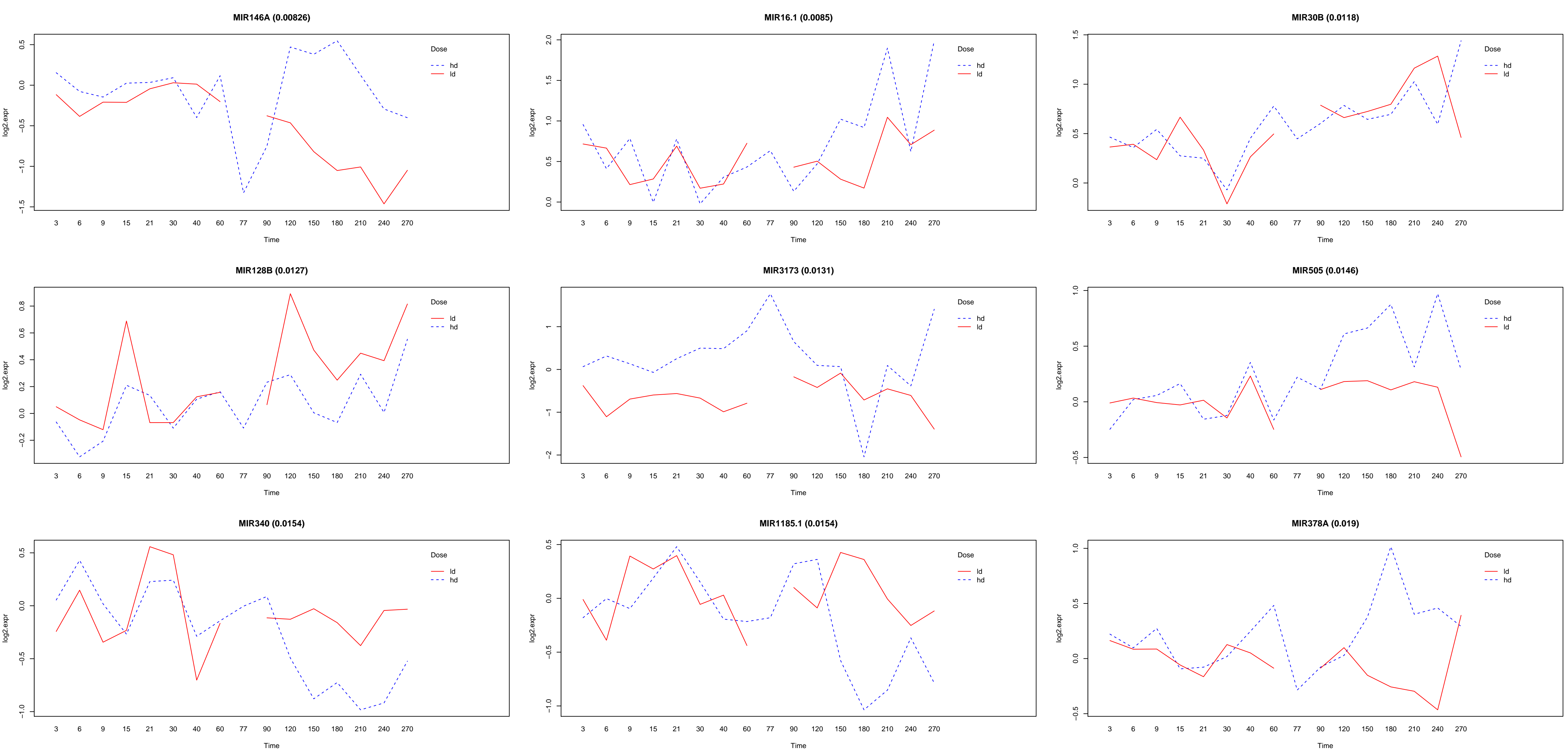

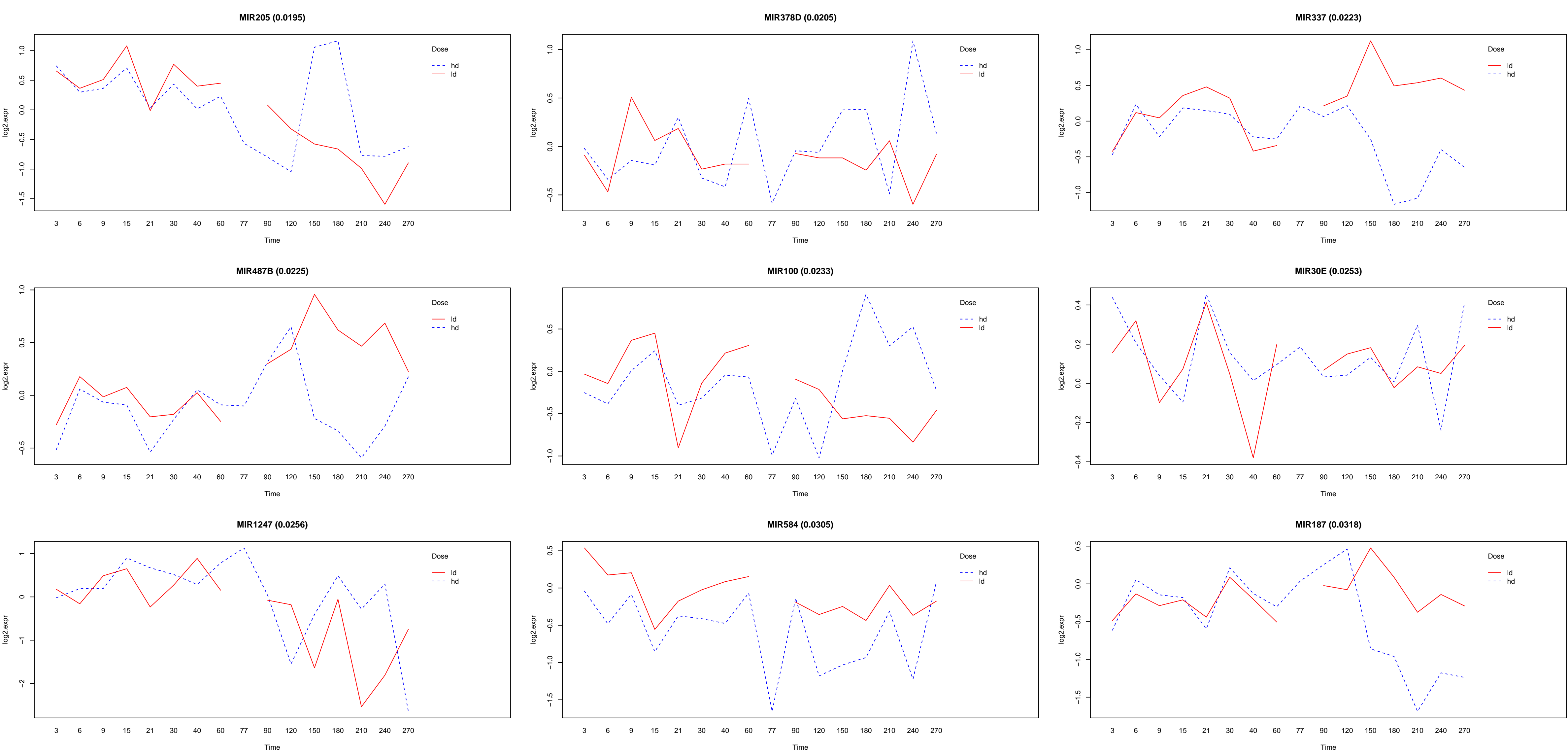

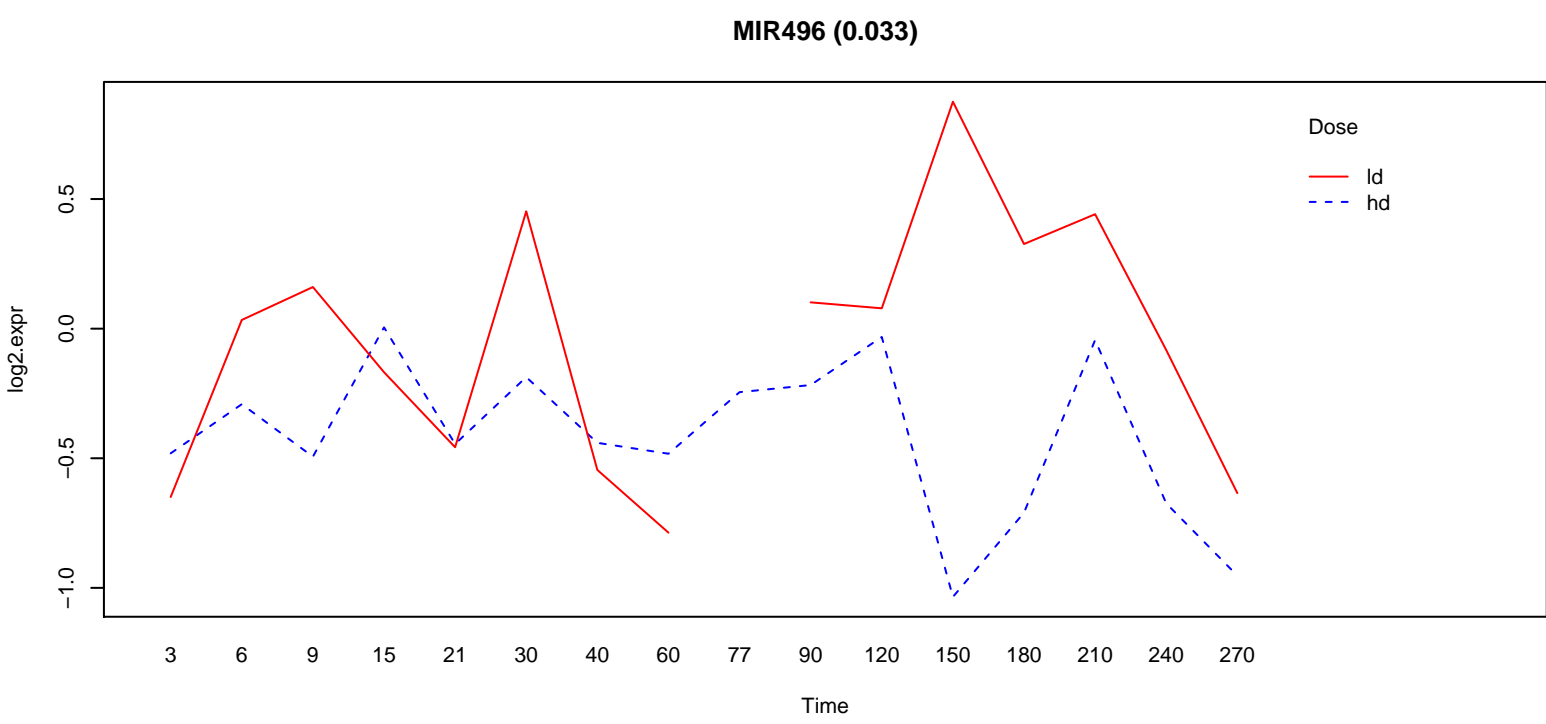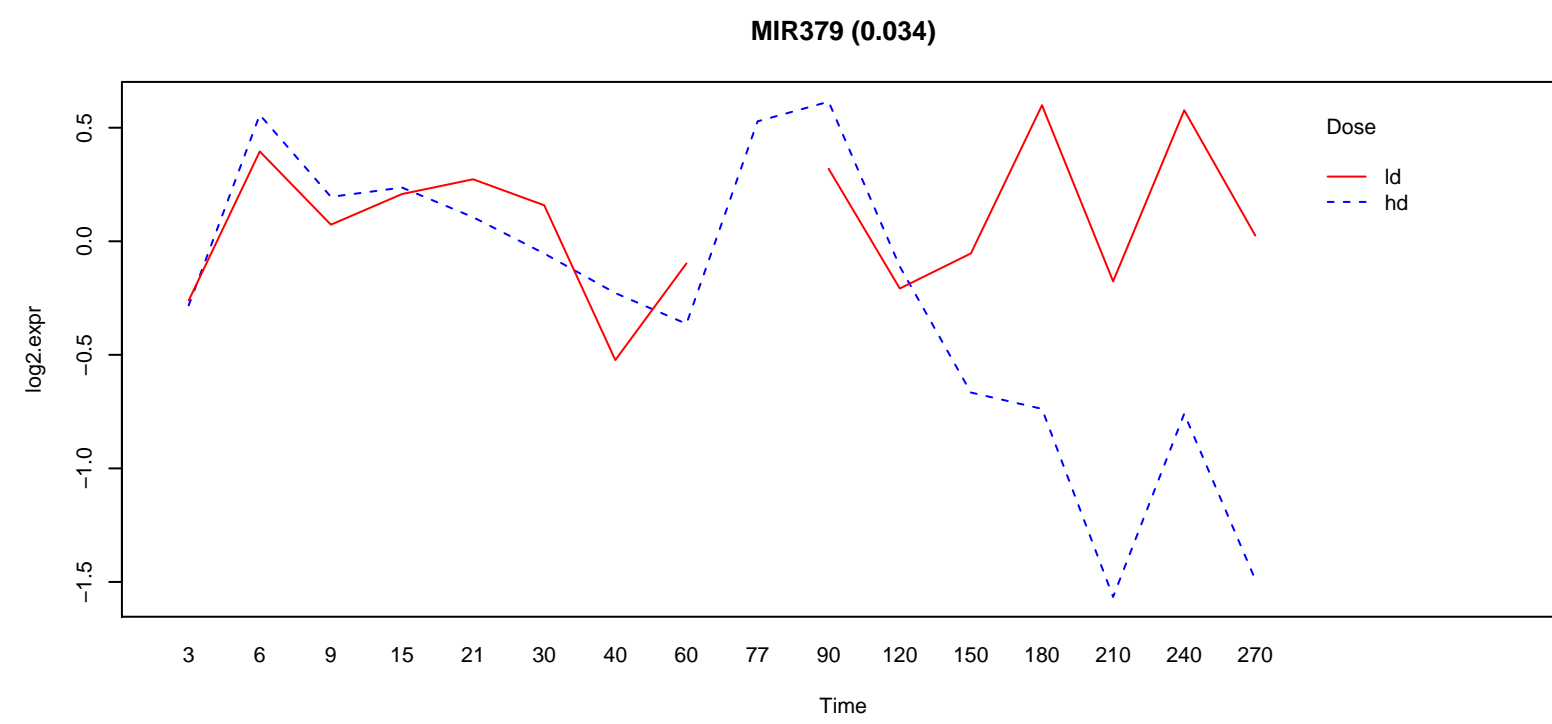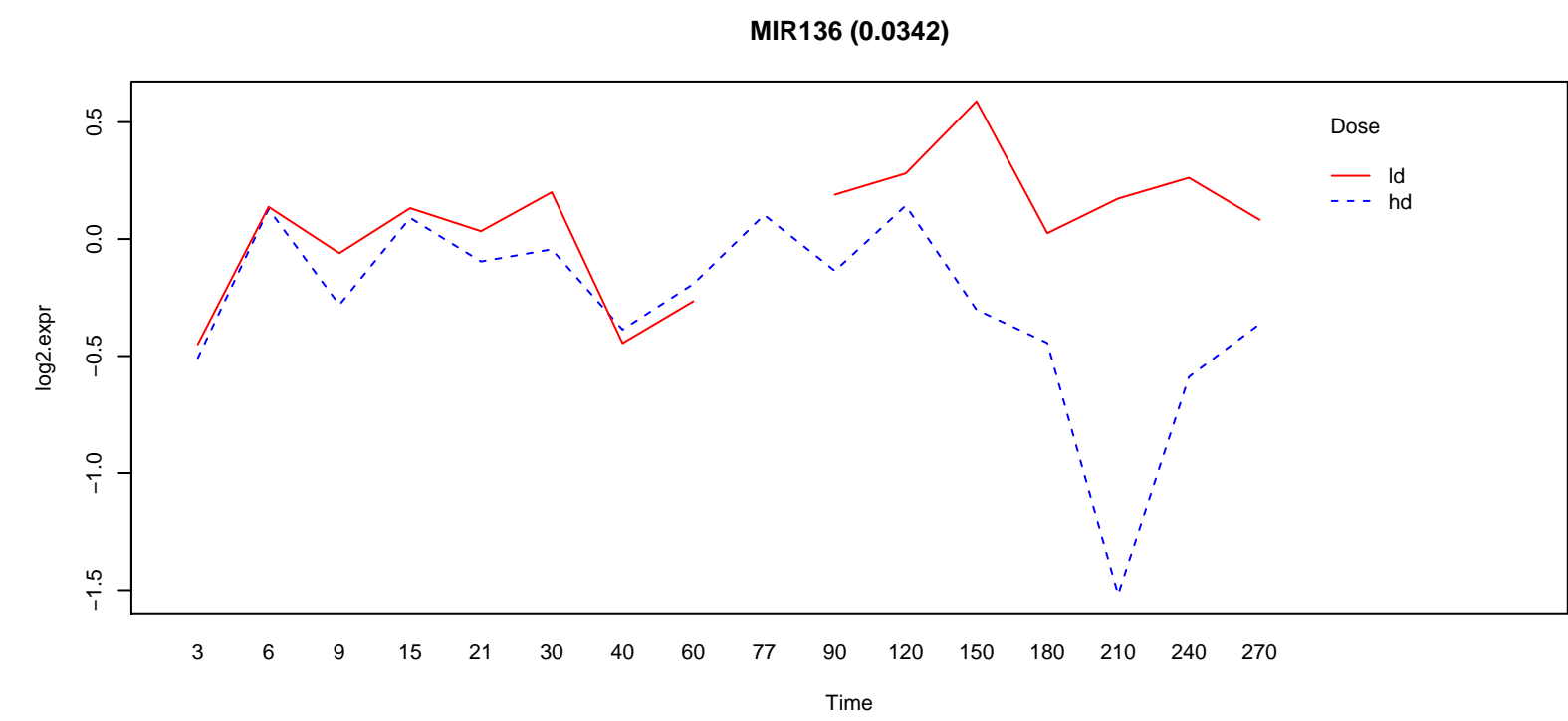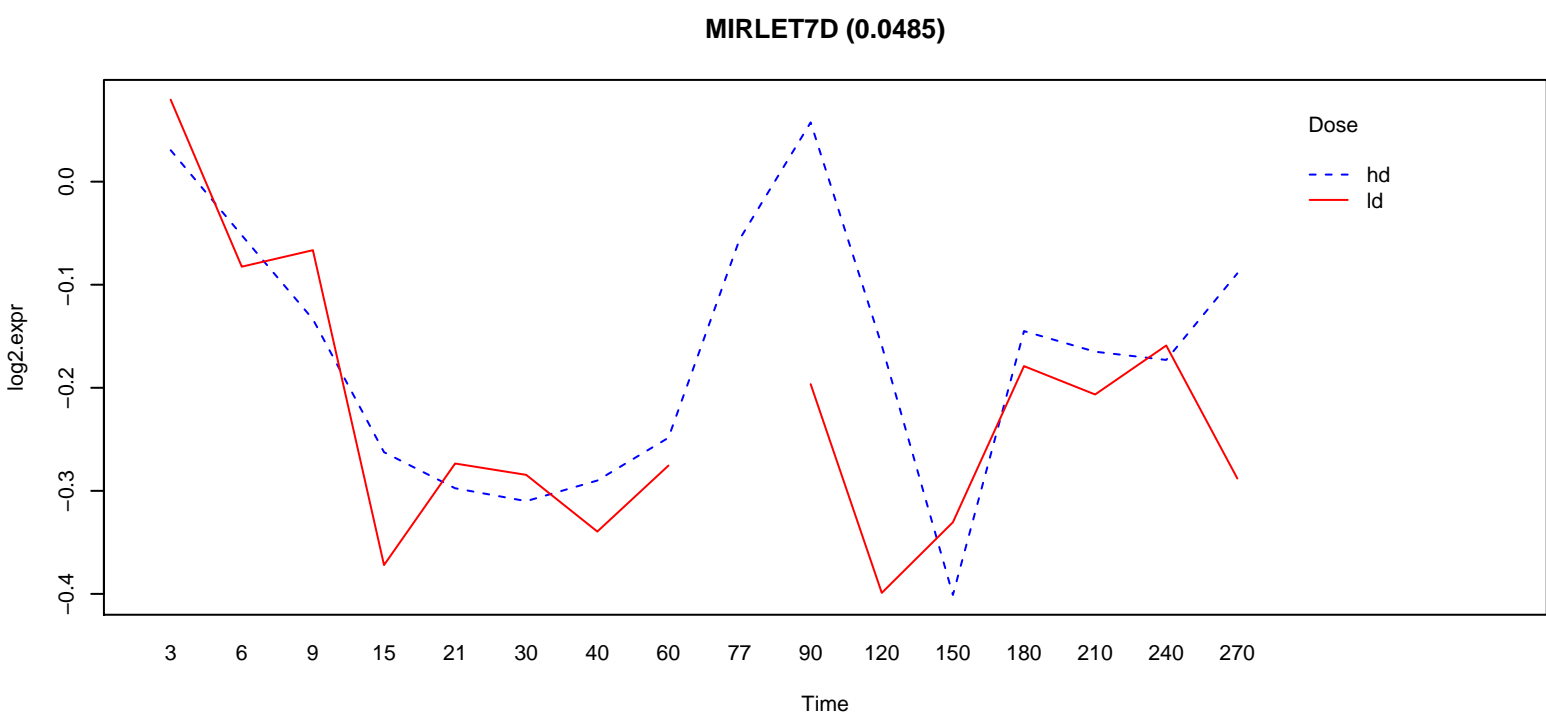

Supplement: Supplementary file 3 — Supplementary Information 3. [file 41598_2022_16316_MOESM3_ESM.pdf]
