# Supplementary material for: Serum RNA biomarkers for predicting survival in non-human primates following thoracic radiation
Source: Sci Rep. 2022 Jul 19;12:12333. doi: 10.1038/s41598-022-16316-x (PMC9296457; doi:10.1038/s41598-022-16316-x)
Supplement: Supplementary file 4 — Supplementary Information 4. [file 41598_2022_16316_MOESM4_ESM.pptx]

## Slide 1
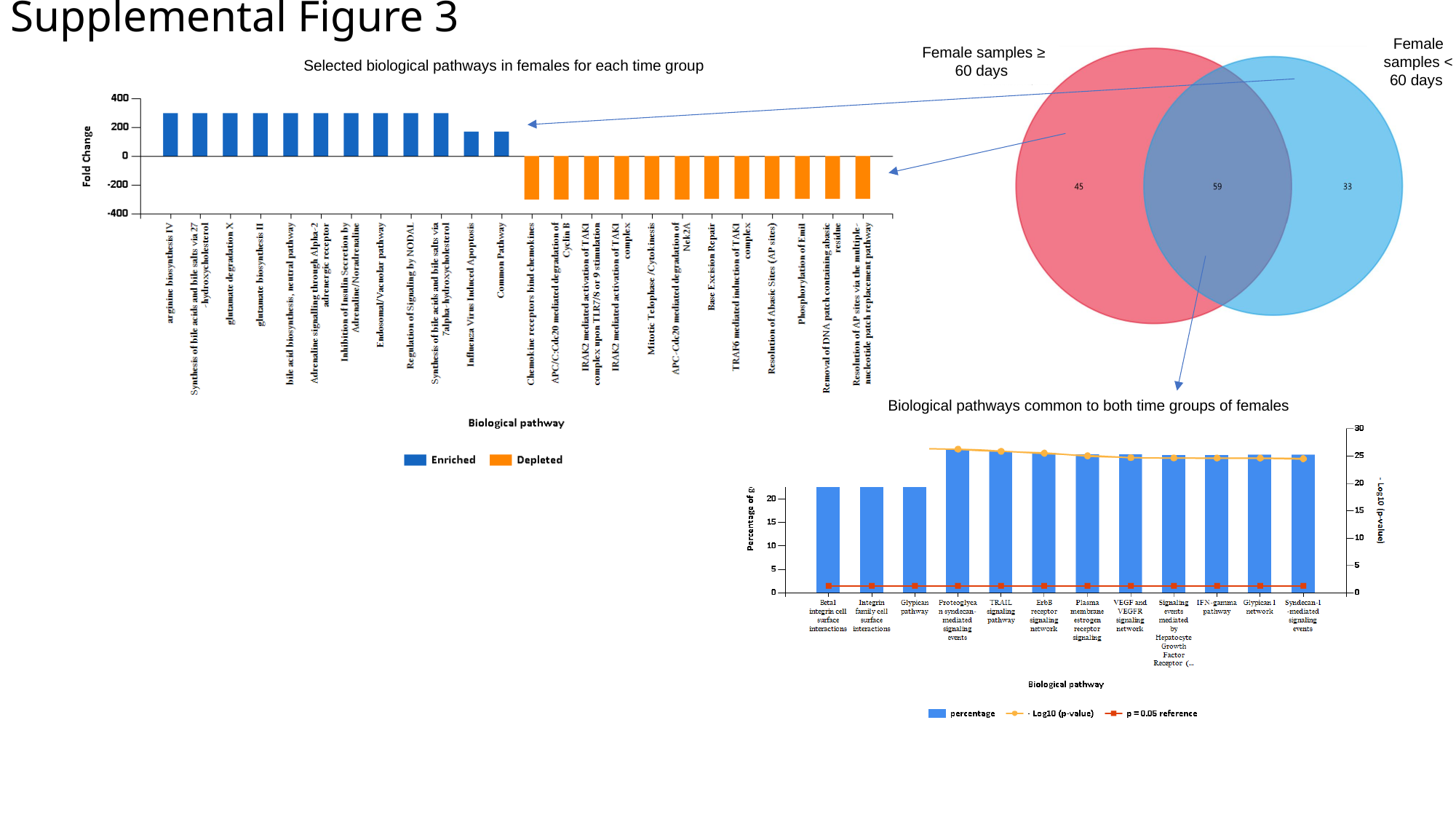

Supplemental Figure 3
Female samples < 60 days
Female samples ≥ 60 days
Selected biological pathways in females for each time group
Biological pathways common to both time groups of females
