# Supplementary material for: Serum RNA biomarkers for predicting survival in non-human primates following thoracic radiation
Source: Sci Rep. 2022 Jul 19;12:12333. doi: 10.1038/s41598-022-16316-x (PMC9296457; doi:10.1038/s41598-022-16316-x)
Supplement: Supplementary file 5 — Supplementary Information 5. [file 41598_2022_16316_MOESM5_ESM.pptx]

## Slide 1
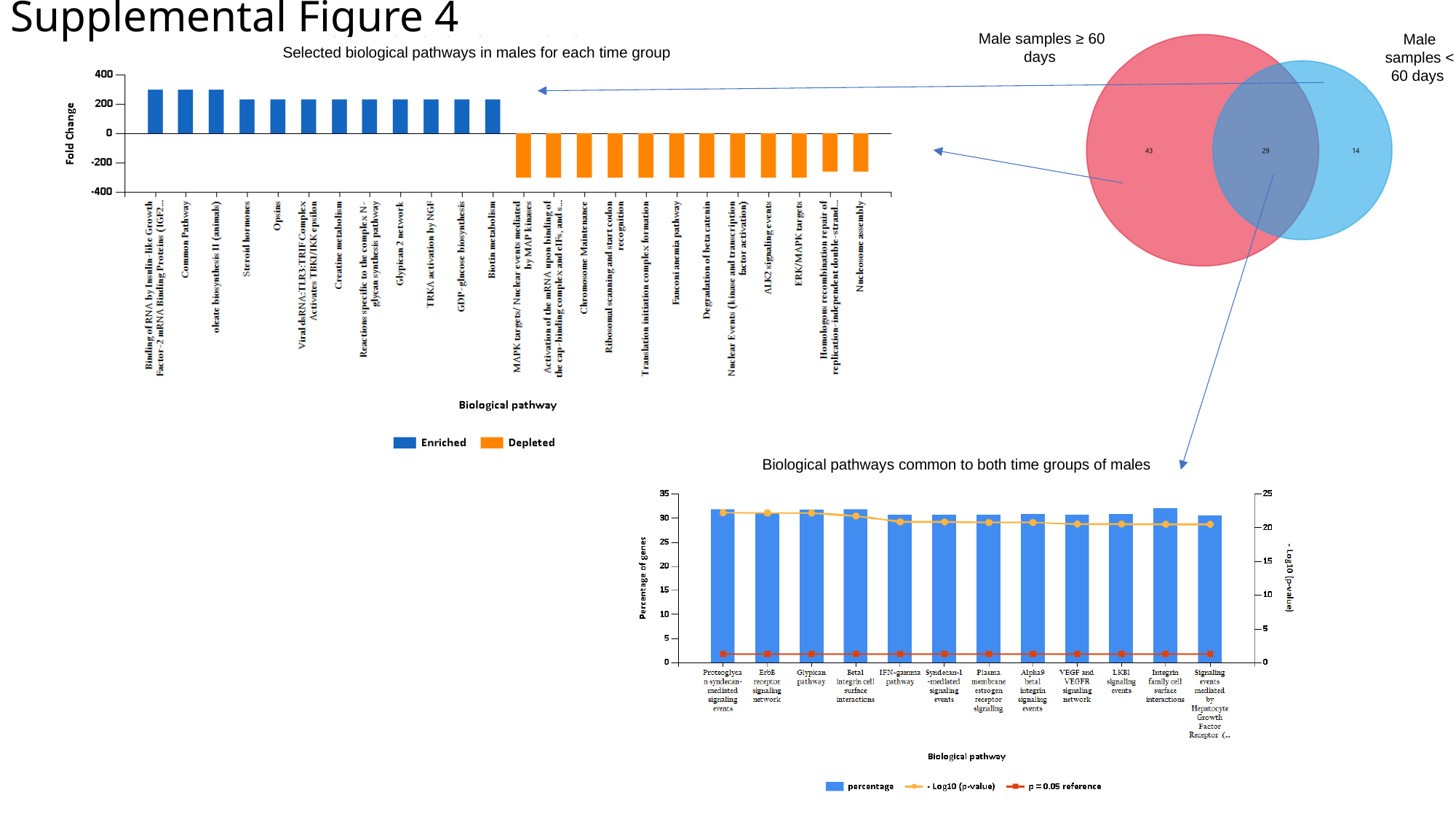

Supplemental Figure 4
Male: LTH vs. GTH
Male samples ≥ 60 days
Male samples < 60 days
Selected biological pathways in males for each time group
Biological pathways common to both time groups of males
