# Supplementary material for: Serum RNA biomarkers for predicting survival in non-human primates following thoracic radiation
Source: Sci Rep. 2022 Jul 19;12:12333. doi: 10.1038/s41598-022-16316-x (PMC9296457; doi:10.1038/s41598-022-16316-x)
Supplement: Supplementary file 7 — Supplementary Information 7. [file 41598_2022_16316_MOESM7_ESM.pptx]

## Slide 1
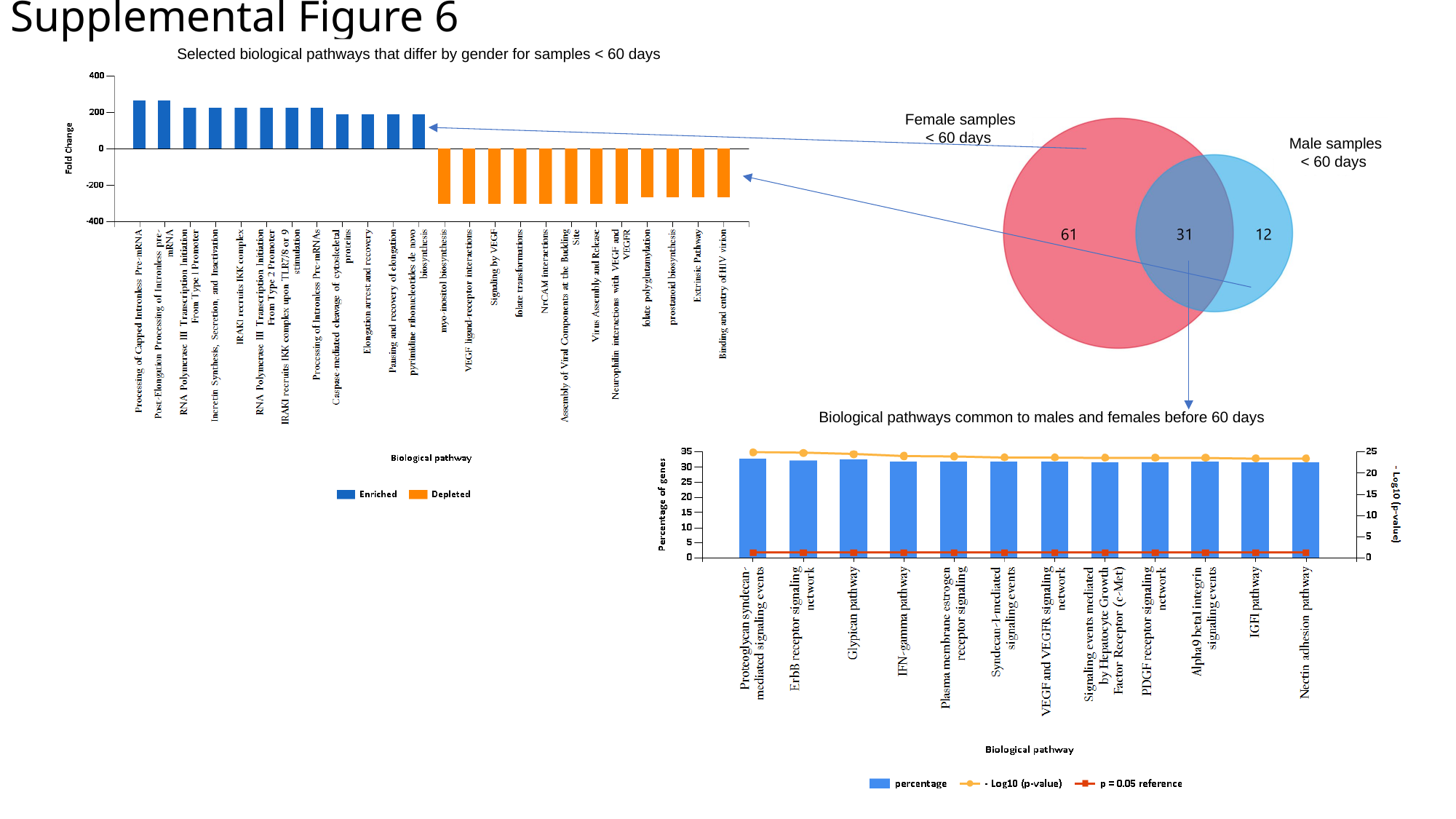

Supplemental Figure 6
Selected biological pathways that differ by gender for samples < 60 days
Female samples < 60 days
Male samples < 60 days
Biological pathways common to males and females before 60 days
